# Uncovering biosynthetic relationships between antifungal nonadrides and octadrides†

**DOI:** 10.1039/d0sc04309e

**Published:** 2020-10-07

**Authors:** Kate M. J. de Mattos-Shipley, Catherine E. Spencer, Claudio Greco, David M. Heard, Daniel E. O'Flynn, Trong T. Dao, Zhongshu Song, Nicholas P. Mulholland, Jason L. Vincent, Thomas J. Simpson, Russell J. Cox, Andrew M. Bailey, Christine L. Willis

**Affiliations:** School of Chemistry, University of Bristol Cantock's Close Bristol BS8 1TS UK Chris.Willis@bristol.ac.uk andy.bailey@bristol.ac.uk kd4495@bristol.ac.uk; School of Biological Sciences, University of Bristol 24 Tyndall Avenue Bristol BS8 1TQ UK; Syngenta, Jealott's Hill International Research Centre Bracknell RG42 6EY UK; Institute for Organic Chemistry and BMWZ, Leibniz University of Hannover Schneiderberg 38 30167 Hannover Germany

## Abstract

Maleidrides are a class of bioactive secondary metabolites unique to filamentous fungi, which contain one or more maleic anhydrides fused to a 7-, 8- or 9- membered carbocycle (named heptadrides, octadrides and nonadrides respectively). Herein structural and biosynthetic studies on the antifungal octadride, zopfiellin, and nonadrides scytalidin, deoxyscytalidin and castaneiolide are described. A combination of genome sequencing, bioinformatic analyses, gene disruptions, biotransformations, isotopic feeding studies, NMR and X-ray crystallography revealed that they share a common biosynthetic pathway, diverging only after the nonadride deoxyscytalidin. 5-Hydroxylation of deoxyscytalidin occurs prior to ring contraction in the zopfiellin pathway of *Diffractella curvata*. In *Scytalidium album*, 6-hydroxylation – confirmed as being catalysed by the α-ketoglutarate dependent oxidoreductase ScyL2 – converts deoxyscytalidin to scytalidin, in the final step in the scytalidin pathway. Feeding scytalidin to a zopfiellin PKS knockout strain led to the production of the nonadride castaneiolide and two novel ring-open maleidrides.

## Abstract 2

Deoxyscytalidin is a common biosynthetic intermediate to the nonadride scytalidin in the fungus *Scytalidium album* and in *Diffractella curvata* gives the octadride zopfiellin.

## Graphical abstract

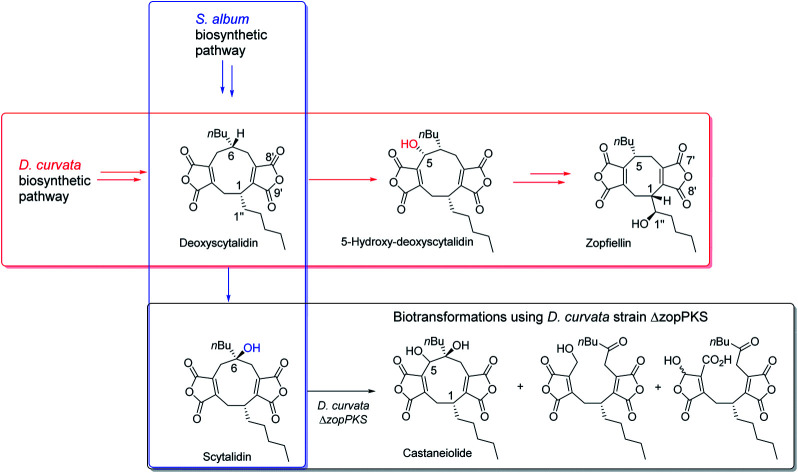

## Introduction

**Fig. 1:**
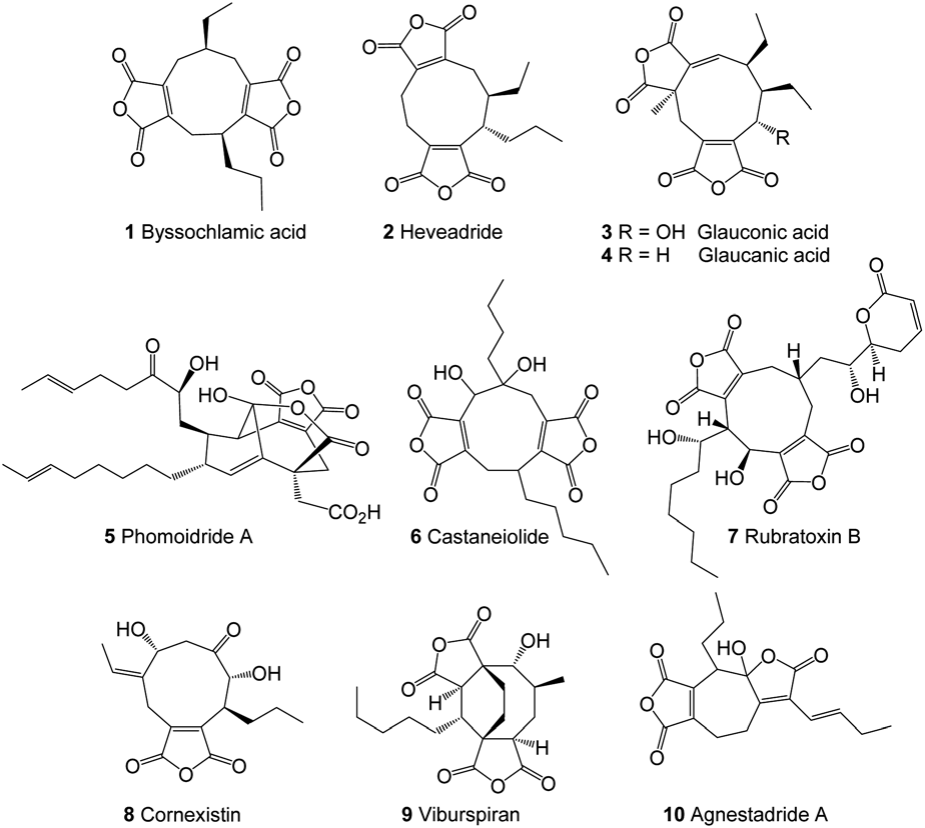
Examples of maleidrides.

Fungal maleidrides are an important family of polyketide-derived secondary metabolites which exhibit a diversity of biological activities including as antifungal^1,2^ and herbicidal^3,4^ agents.^5^ They are characterised by a medium-sized alicyclic ring with one or two fused maleic anhydride moieties. The majority of the reported maleidrides are nonadrides assembled on a 9-membered ring core, and early examples include byssochlamic acid **1**,^6^ heveadride **2**,^7^ glauconic acid **3** and glaucanic acid **4** (Fig. 1).^8,9^ Later studies revealed further nonadrides such as the phomoidrides, *e.g.***5**,^10^ castaneiolide **6**,^11^ rubratoxins, *e.g.***7** ^12^ and cornexistin **8**,^3,13^ the octadride viburspiran **9** and more recently two heptadrides agnestadrides A **10** and B have been isolated from *Byssochlamys fulva*.^14^ In certain cases the relative and absolute stereochemistry of the maleidrides remain unknown, and various details of their biosynthesis are yet to be fully elucidated.

**Scheme 1:**
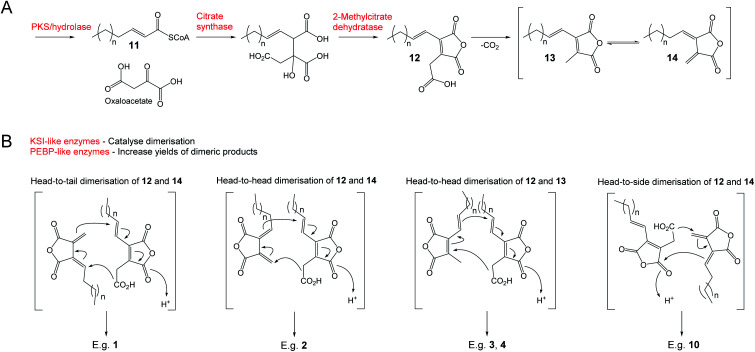
The universal pathway to maleidrides^16^ involves the production of maleic anhydride monomers (A) followed by dimerisation (B) – the mode of which determines the carbon framework of the final compound.

Results of extensive studies on selected nonadrides using isotopic labelling, gene knock outs and heterologous expression experiments have led to the proposal that the biosynthetic pathway begins with the assembly of an unsaturated precursor **11***via* an iterative highly reducing polyketide synthase (hrPKS), where the chain length varies according to the structure of the natural product (Scheme 1). Coupling of **11** with oxaloacetate is catalysed by citrate synthase-like enzymes and is followed by dehydration catalysed by 2-methylcitrate dehydratases to generate the maleidride monomer **12**.^15,16^ Decarboxylation of **12** then gives the tautomeric compounds **13** and **14** (Scheme 1A).^14,17^ The second stage of the biosynthetic pathway, coupling the monomers in various modes and cyclisation to the carbocyclic ring, involves ketosteroid isomerase-like (KSI) and phosphatidylethanolamine binding protein-like (PEBP) enzymes.^16^ The mode of cyclisation determines the carbon framework of the maleidride (Scheme 1B), and finally tailoring modifications decorate these structures leading to the observed diversity of natural products.

**Fig. 2:**
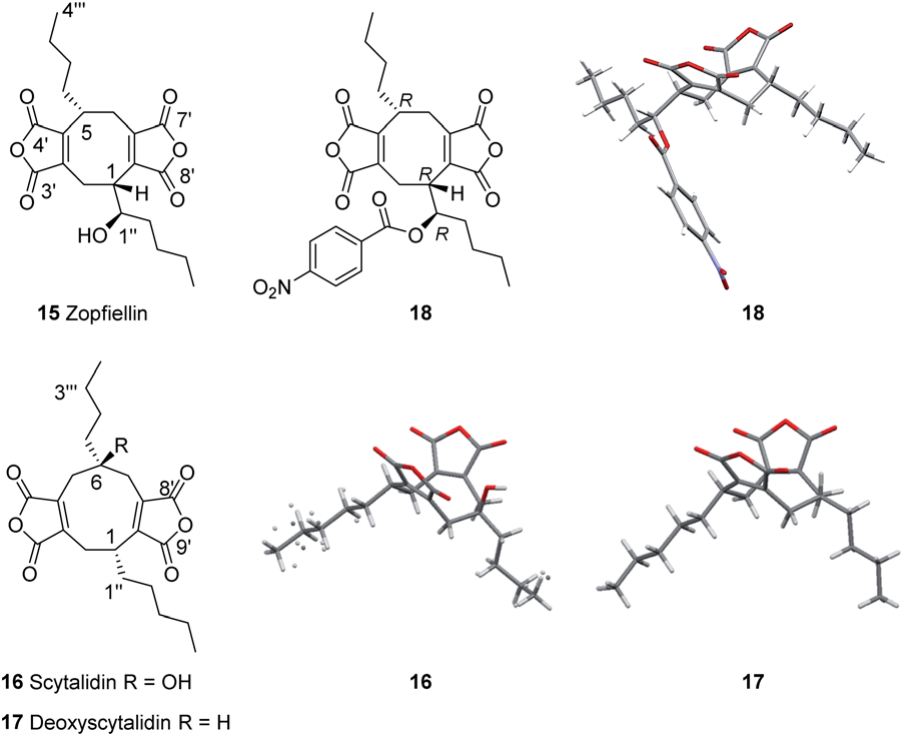
Confirmed structures of the natural products **15**, **16** and **17**.

Octadrides, with 8-membered rings, are less common than nonadrides and include viburspiran **9** ^18^ and zopfiellin **15** ^19^ (Fig. 1 and 2). The biosynthetic origin of the 8-membered ring is unknown (§§The numbering systems used for the maleidrides varies greatly in the literature and shows no consistency. We propose a common more systematic system based on the size of the ring (1–9, 1–8, 1–7 as appropriate) beginning at the carbon alpha to the maleic anhydride ring, which gives the lowest numbers to the side chains. The maleic anhydride carbons would be numbered with a prime, appropriate to the ring numbering, hence 3′4′ and 8′9′ for byssochlamic acid, and 1′′, 2′′, *etc.* for the first side chain, numbering from the ring junction and 1′′′, 2′′′, *etc.* for the second side chain.see footnote). Zopfiellin was first isolated from *Zopfiella curvata* and the structure determined by a combination of spectroscopic methods.^19–21^ The relative and absolute configurations of zopfiellin have been indicated in several publications,^22–24^ but we were unable to find any justification of the configuration of the side chain secondary alcohol. Hence, with our continuing interest in the biosynthesis of fungal natural products, and in particular bioactive maleidrides, our goal was to confirm the structure of zopfiellin **15** and determine the biosynthetic origin of the 8-membered ring, with the longer-term goal of engineering the pathway to new antifungal targets.

Whilst this manuscript was in preparation Minami, Oikawa and co-workers reported investigations into the biosynthesis of zopfiellin using isotopic labelling, heterologous expression of candidate genes in *Aspergillus oryzae*, combined with structure elucidation of minor metabolites. These studies showed that a ring contraction occurs from a nonadride to an octadride.^25^ Herein we describe our recent studies which confirm the absolute and relative configurations of the nonadride scytalidin **16** and octadride zopfiellin **15** and reveal that they share a common biosynthetic precursor, deoxyscytalidin **17** (Fig. 2). Our biosynthetic investigations involved a different approach from those previously reported^25^ and used a combination of gene disruptions in *Diffractella curvata* and *Scytalidium album*, feeding studies to mutant strains and full structure elucidation of known and novel compounds. These studies establish the biosynthetic relationships between zopfiellin **15**, scytalidin **16**, and deoxyscytalidin **17**.

## Results and discussion

The confirmed zopfiellin producer, *Zopfiella curvata* no. 37-3, was not publicly available when these studies began, so *Diffractella curvata* CBS591.74 was obtained and confirmed to produce zopfiellin. Cultures were grown in shake flasks in PDB medium at 25 °C for 14 days and after purification by flash chromatography, gave zopfiellin **15** (80–100 mg L^−1^) as an oil. As full assignment of NMR data had not previously been reported^19,21^ 2D NMR data (COSY, HSQC and HMBC) analysis allowed assignment of the ^1^H and ^13^C NMR spectra (Table S6 and Fig. S31–S36†). The optical rotation, [*α*]_D_ −84.3 (*c* 0.43 MeOH) was consistent with the literature value, [*α*]_D_ −76.8 (*c* 0.42 MeOH).^20^ Zopfiellin was esterified to give the novel *p*-nitrobenzoate **18** and X-ray crystallography revealed the relative configuration of the natural product (Fig. 2). Synthesis and analysis of the (*R*)- and (*S*)-Mosher's ester derivatives of **15** confirmed the absolute stereochemistry of zopfiellin as 1*R*, 5*R*, 1′′*R* (Fig. S81–S83†).

**Fig. 3:**
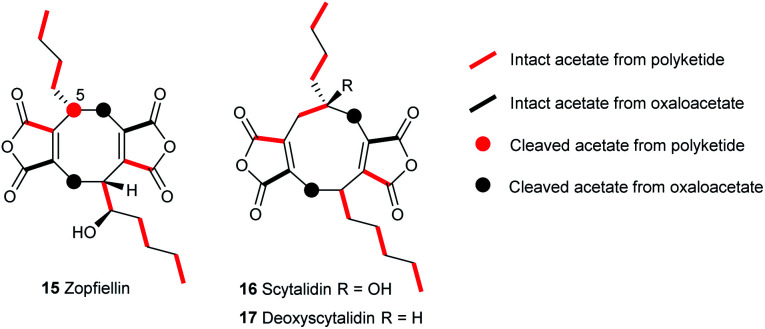
Incorporation patterns in octadrides and nonadrides from feeding studies with [1,2-^13^C_2_]-acetate.

Feeding [1,2-^13^C_2_]-acetate to cultures of *D. curvata* showed mainly intact incorporation of acetate into zopfiellin **15** (Fig. 3). Enhancement of C-5 in the ^13^C NMR spectrum was observed, but there was no coupling to an adjacent ^13^C, indicating that it originates from a cleaved acetate and therefore that a carbon atom is lost during zopfiellin biosynthesis.

A possible biosynthetic precursor to zopfiellin is therefore a nonadride which on rearrangement with the loss of one carbon atom would give the corresponding octadride. Such ring contractions are well precedented in fungal biosynthesis, for example, in the contraction of ring B of the kaurenoids to the gibberellins,^26^ the conversion of the 14-membered polyketide macrodiolide colletotriene to the 13-membered ring of bartanol^27^ and the oxidative ring contractions seen in xenovulene biosynthesis.^28^ Conceivably the known natural products scytalidin **16** ^29^ (C_22_H_28_O_7_), deoxyscytalidin **17** ^30^ (C_22_H_28_O_6_) or castaneiolide **6** ^11^ (C_22_H_28_O_8_) could be precursors. To obtain samples of **16** and **17**, cultures of *S. album* strain UAMH 3620 were grown, and after 14 days compounds **16** and **17** were isolated in similar titres (*ca*. 10 mg L^−1^). X-ray analysis confirmed that the alkyl side chains are *syn* in both metabolites (Fig. 2). The results of feeding studies using [1,2-^13^C_2_] acetate to cultures of *S. album* were in accord with the predicted biosynthetic pathway to nonadrides and that one or both could possibly be biosynthetic precursors of zopfiellin **15** (Fig. 3).

**Fig. 4:**
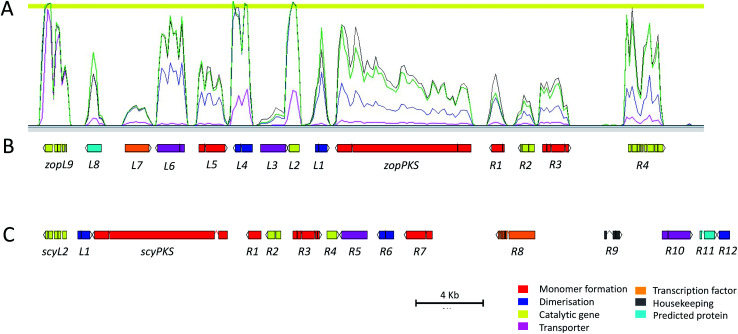
(A) RNAseq data mapped to the zopfiellin BGC showing coregulation of the genes under four different conditions (Fig. S1 and S2†). Pink: GN. Purple: CDB. Green: PDB-day 5. Black: PDB-day 8. (B) The zopfiellin gene cluster, as annotated in Artemis, which contains genes *zopL9* to *zopR4*. (C) The scytalidin gene cluster, as annotated in Artemis, which contains genes *scyL2* to *scyR12*. ‘L’ in the gene names denotes genes to the left, or upstream, of the PKS. ‘R’ denotes genes to the right, or downstream, of the PKS. Putative gene functions are shown in Table 1.

*De novo* genome sequencing was performed for *D. curvata* CBS591.74 and *S. album* UAMH3620, and an initial screen of the genome data, searching for homologues to the byssochlamic acid biosynthetic gene cluster (BGC),^16^ revealed putative maleidride gene clusters within both genomes. The gene clusters were analysed in detail using FGeneSH^31^ to predict coding sequences, and manually annotated in Artemis^32^ to produce full draft BGCs (Fig. 4 and Table 1). Putative gene functions were assigned *via* the identification of homologues in the NCBI database and the detection of conserved domains using InterPro^33^ (Tables S1 and S2†). Transcriptomic analysis for *D. curvata* under a range of zopfiellin **15** production and non-production conditions aided identification of the cluster boundaries by clearly identifying a co-transcribed region that includes genes from *zopL9* to *zopR4* (Fig. 4, S1 and S2†). In addition, detailed analysis of the transcriptomic data confirmed the positions of all introns and exons in the predicted ORFs. However, despite repeated attempts, it was not possible to generate quality RNAseq data for the putative scytalidin gene cluster in *S. album*.

**Table 1:** Predicted functions for genes in the zopfiellin and scytalidin BGCs. Function were determined by searching for homologues within the Swissprot database and identifying conserved protein domains using InterPro.^33^ Only the functions of genes marked ‘*’ were assigned *via* the identification homologues within NCBIs non-redundant database. See Tables S1 and S2 for further details

Gene	Putative function	Homologue	Gene	Putative function	Homologue
*zopL9*	Hydroxylase/Desaturase	DES^34^	*scyL2*	Hydroxylase	AclN^36^
		AsaB^35^			MfR1 ^37^
*zopL8*	Hypothetical protein	—	scyL1*	PEBP	BfL5 ^16^
*zopL7*	Transcription factor (TF)	AlnR^38^	*scyPKS*	hrPKS	Tox1-PKS^39^
*zopL6*	Major facilitator superfamily (MFS) transporter	Itp1 ^40^	*scyR1*	DUF341 hydrolase/esterase	Fub4 ^41^
*zopL5*	2-Methylcitrate dehydratase (2MCDH)	PrpD^42^	*scyR2*	Enoyl CoA hydratase	Ech1 ^43^
zopL4*	Ketosteroid isomerase-like protein (KSI)	BfL6 ^16^	*scyR3*	Citrate synthase	MfR3 ^44^
*zopL3*	Major facilitator superfamily (MFS) transporter	Itp1 ^40^	*scyR4*	Isochorismatase-like hydrolase/amidohydrolase	CSHase^45^
*zopL2*	Isochorismatase-like hydrolase/amidohydrolase	NicF^46^	*scyR5*	MFS transporter	MfM6 ^44^
			scyR6*	KSI	BfL6 ^16^
*zopL1*	Phosphatidylethanolamine-binding protein (PEBP)	Tfl1 ^47^	*scyR7*	2-Methylcitrate dehydratase	PrpD^42^
*zopPKS*	hrPKS	Tox1-PKS^39^	*scyR8*	Transcription factor	AlnR^38^
*zopR1*	DUF341 hydrolase/esterase	Fub4 ^41^	*scyR9*	Histone H2A	H2A.Z ^48^
*zopR2*	Enoyl-CoA hydratase	Ech1 ^43^	*scyR10*	MFS transporter	MfR5 ^44^
*zopR3*	Citrate synthase	CshA^49^	*scyR11*	Hypothetical protein	—
*zopR4*	FAD-dependent oxidoreductase	YanF^50^	scyR12*	PEBP	BfL9 ^16^
		Sol5 ^51,52^			

**Fig. 5:**
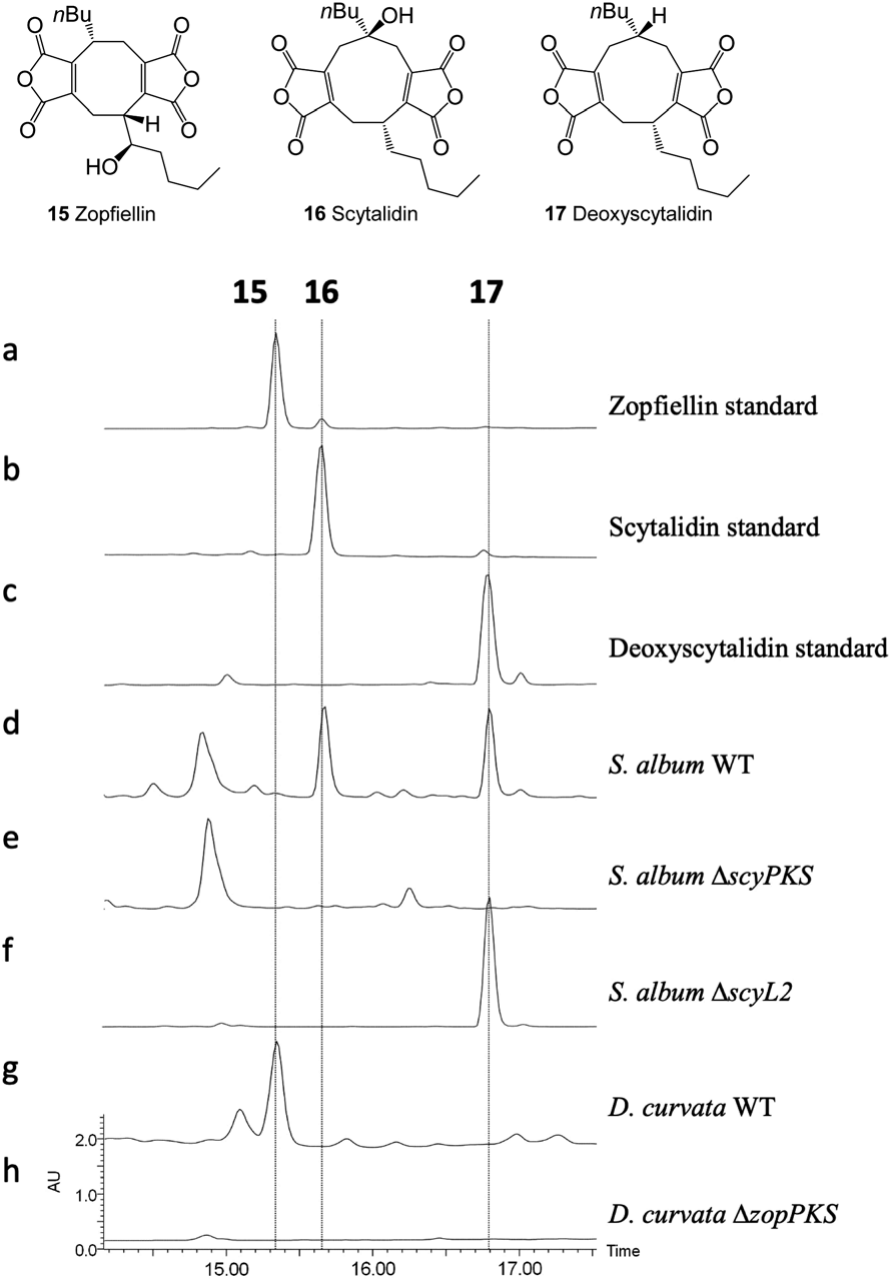
HPLC (DAD) analysis of gene deletion strains for the zopfiellin and scytalidin gene clusters. Disruption of *zopPKS* and *scyPKS* abolished maleidride production in both species, confirming the identity of the zopfiellin and scytalidin BGCs. Disruption of *scyL2* confirmed its role in the final hydroxylation of the scytalidin pathway.

To confirm the identity of the two gene clusters, protoplast-mediated transformation protocols were developed for both fungal species and the PKS genes; *zopPKS* and *scyPKS*, were disrupted using the bipartite gene knock-out approach developed by Nielsen *et al.*^53^ In both cases this led to a total loss of maleidride biosynthesis, with no scytalidin **16** or deoxyscytalidin **17** being present in the crude extracts of the Δ*scyPKS* strains (Fig. 5e), and no zopfiellin **15** being present in the crude extracts of the Δ*zopPKS* strains (Fig. 5h).

**Fig. 6:**
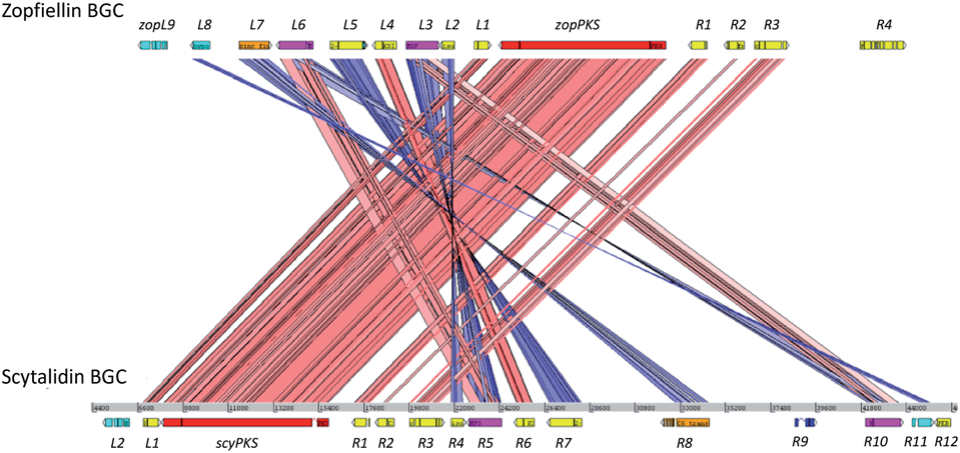
An ACT (Artemis Comparison Tool) comparison of the zopfiellin and scytalidin BGCs identified genes predicted to encode homologous proteins and highlighted the similarity between the two biosynthetic gene clusters.

An ACT (Artemis Comparison Tool)^54^ comparison of the zopfiellin and scytalidin BGCs (Fig. 6) highlighted significant homologies. Both clusters contain genes encoding the essential hrPKS, DUF341 hydrolase, citrate synthase (CS) and 2-methylcitrate dehydratase (2MCDH),^12,13,15,16^ as well as the ‘dimerisation’ genes encoding a ketosteroid-isomerase-like protein (KSI) and a PEBP-like protein.^16^ In the case of the scytalidin BGC a second PEBP gene is present, which has been observed previously in the byssochlamic acid and rubratoxin BGCs.^12,16^ A domain analysis of the two PKS genes identified all of the domains typically present in a highly-reducing PKS: KS, AT, DH, *C*MeT, ER, KR and ACP. Additional genes identified in both clusters encode an enoyl-CoA hydratase, an isochorismatase, a small hypothetical protein (*zopL8* and *scyR11*), a GAL4 type transcription factor and two MFS (major facilitator superfamily) transporters. The enoyl-CoA hydratase encoded by the zopfiellin cluster (*zopR2*) was not identified in the work by Oikawa and colleagues,^25^ but RNAseq data confirmed its annotation.

The proteins encoded by the clusters also have high sequence identity (Table S3†). The KSI and PEBP-like proteins, for example, are far more similar to one another (80.7% and 66.2% identity respectively, Table S3†) than to those identified in other maleidride BGCs (Fig. S4–S7†). In the case of the transporters, ZopL6 and ScyR5 are highly homologous (80.4% identity) and thus may be expected to transport similar compounds. The other two transporters, ZopL3 and ScyR10, are more diverse, sharing only 38.5% identity. All other homologues encoded by the gene clusters share over 50% identity, with many sharing over 80% (Table S3†).

The only proteins which are uniquely encoded by the zopfiellin gene cluster are a putative hydroxylase/desaturase (ZopL9), which shares homology with DES from the gibberellin pathway, and an FAD-dependent oxidoreductase (ZopR4). The scytalidin gene cluster encodes only one unique protein, a putative hydroxylase (ScyL2). ZopL9 and ScyL2 are both predicted to be α-ketoglutarate (αKG) dependent enzymes based on the presence of conserved iron and αKG binding residues (Fig. S10†), but they were not considered to be homologues due to the low level of sequence identity (approximately 25% identity), suggesting that the exact catalytic function is unlikely to be the same. Also located within the scytalidin BGC is a gene (*scyR9*) encoding a highly conserved H2A.F/Z family histone,^55^ but this is unlikely to play a direct role in scytalidin biosynthesis (Table S3†).

**Scheme 2:**
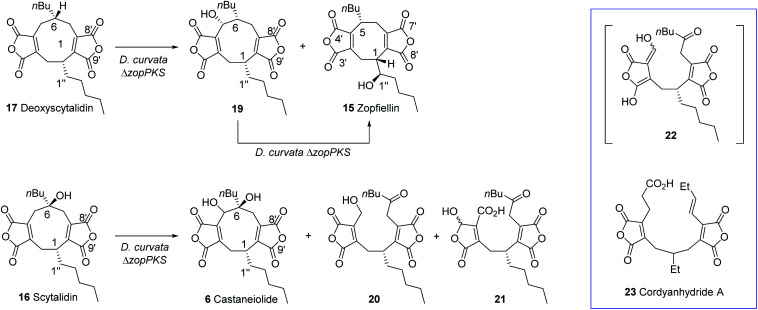
Feeding studies to *D. curvata* strain Δ*zopPKS* identified **17** and **19** as intermediates in zopfiellin biosynthesis and scytalidin was metabolised to novel products.

**Fig. 7:**
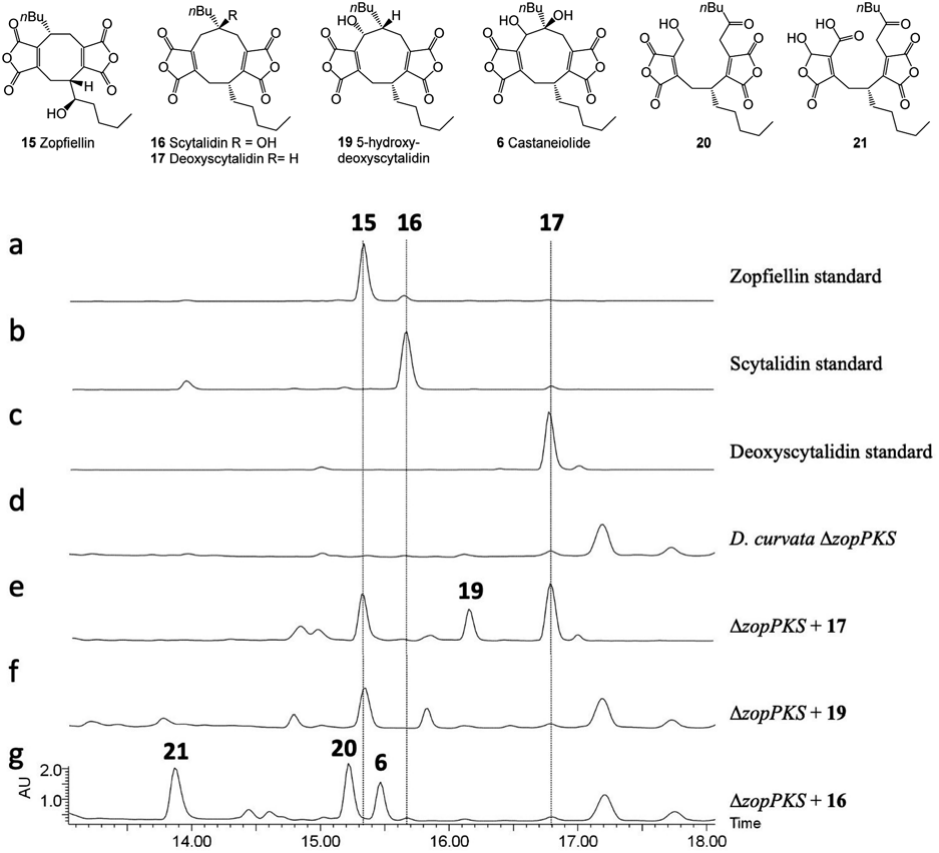
HPLC (DAD) chromatograms of crude extracts. Feeding compounds to *D. curvata* Δ*zopPKS* indicate that deoxyscytalidin **17** and 5-hydroxy-deoxyscytalidin **19** are intermediates in the zopfiellin biosynthetic pathway, whereas scytalidin **16** is not. Feeding of **16** led to the production of castaneiolide **6** and two novel ring-open anhydrides **20** and **21**.

The striking similarity between the two gene clusters (Fig. 6) suggests that zopfiellin **15** and scytalidin **16** have very similar biosynthetic origins, and that either deoxyscytalidin **17** or scytalidin **16** may be precursors of zopfiellin. To test this theory, **16** and **17** were purified from *S. album* cultures and separately fed to *D. curvata* Δ*zopPKS* cultures on days 3 and 5 post-inoculation (5 mg per day per 100 ml of culture). In cultures fed with **17**, zopfiellin production was restored (Scheme 2 and Fig. 7e), demonstrating that deoxyscytalidin **17** is a precursor of zopfiellin. The extent of conversion of deoxyscytalidin to zopfiellin varied between individual feeds, presumably due to varying expression levels for the zopfiellin BGC. In cultures where the conversion was partial, the HPLC trace showed a peak for deoxyscytalidin, as well as a novel compound with a mass of 404 eluting at 16.1 minutes (Fig. 7e). This metabolite was isolated, and the structure confirmed by NMR spectroscopy to be 5-hydroxy-deoxyscytalidin **19** (Table S9 and Fig. S44–S49†). The signal assigned to 5-H (at *δ* 4.90) appeared as a broad singlet and NOE studies were in accord with hydroxylation occurring on the same face as the butyl side chain at C-6. To determine whether **19** is a shunt product or an intermediate on the biosynthetic pathway, it was fed to *D. curvata* Δ*zopPKS* cultures and again this restored zopfiellin biosynthesis (Scheme 2 and Fig. 7f).

When scytalidin **16** was fed to cultures of *D. curvata* Δ*zopPKS*, no zopfiellin was detected, but several additional compounds were present when compared to controls (Fig. 7g). The mixture was purified by HPLC giving the known^11^ nonadride castaneiolide **6** as one of the metabolites, presumably formed by 5-hydroxylation of scytalidin **16** (Scheme 2). In addition, two novel compounds **20** and **21** were isolated (3.2 mg and 0.7 mg respectively) and their structures elucidated by MS and extensive NMR studies (Table S10 and Fig. S50–S63†). The ^13^C NMR spectrum of compound **20** (C_22_H_28_O_8_ from HRMS) showed downfield signals assigned to the two maleic anhydrides, as well as an additional signal at *δ*_C_ 205 ppm assigned to a ketone. The ^1^H-NMR showed characteristic AB signals assigned to the hydroxymethylene. Based on this evidence, and combined with further analysis of 2D NMR data, the new metabolite was assigned as the ring cleaved alcohol **20**. The spectral data of **21** was also in accord with a ring cleaved metabolite with a ketone in the side chain, but in this case the primary alcohol was oxidised to a carboxylic acid and one of the carbonyls of the anhydride reduced to give a mixture of epimers (*δ*_C_ 96.9/97.4 ppm, *δ*_H_ 6.19/6.23 ppm). Such reductions of anhydrides are commonly observed in maleidride biosynthesis.^14,56,57^ We propose that ketone **20** may be formed *via* cleavage of the 5,6-diol **6** to give an intermediate **22**, which would tautomerise to generate ketone **20**. Oxidation of the primary alcohol of **20** to a carboxylic acid and selective reduction of the anhydride would give **21**. Such linear bis(maleic anhydride) structures are not without precedent, for example the fungal metabolite cordyanhydride A **23**.^58^ However, **23** has previously been proposed to arise *via* the linear coupling of two distinct maleic anhydride monomers rather than *via* cleavage of a cyclic intermediate.^15^

Based on the bioinformatic analysis of the BGCs and the identification of deoxyscytalidin **17** as an intermediate to both scytalidin **16** and zopfiellin **15**, it is a reasonable inference that the two biosynthetic pathways proceed in an identical manner to **17**, after which they diverge. Hydroxylation at C-6 would produce scytalidin whereas hydroxylation at C-5, ring contraction and hydroxylation of the side chain would produce zopfiellin.

ScyL2, being the only unique catalytic protein encoded by the scytalidin BGC, was identified as a likely candidate for catalysing the final step in the scytalidin biosynthetic pathway. This is supported by a bioinformatic analysis identifying ScyL2 as a likely α-ketoglutarate dependent hydroxylase (Table S2†), which shares homology with enzymes known to catalyse hydroxylations, such as MfR1 and MfR2 from the squalestatin pathway,^37^ and RbtB and RbtG from the rubratoxin pathway^12^ (Fig. S8–S10†). To confirm this role, *scyL2* knock-out strains of *S. album* were generated. These strains did not produce scytalidin **16**, but accumulated deoxyscytalidin **17** (Fig. 5f), confirming the role of ScyL2.

Recent work by Shiina *et al.*^25^ on the zopfiellin pathway of *Z. curvata*, using heterologous expression as well as *in vitro* assays, has begun to elucidate the later stages of zopfiellin biosynthesis. ZopK, which is equivalent to ZopL9 of the *D. curvata* BGC reported here, was shown to catalyse the oxidative ring contraction in the zopfiellin pathway to produce deoxyzopfiellin **24**.^25^ This proceeds *via* the intermediate **19** (which we now know to be 5-hydroxy-deoxyscytalidin) and is consistent with our identification, *via* feeding studies, of **19** as an intermediate in the zopfiellin pathway. *In vitro* assays using purified recombinant ZopL9 gave results which are entirely consistent with the findings of Shiina *et al.*^25^ In the presence of α-ketoglutarate, ZopL9 was capable of catalysing highly efficient hydroxylation of deoxyscytalidin **17** to give 5-hydroxy-deoxyscytalidin **19** (Fig. S68†). A minor compound was also detected which was purified and identified as deoxyzopfiellin **24**. Repeating the assays using 5-hydroxy-deoxyscytalidin **19** as the substrate also gave rise to deoxyzopfiellin **24** (Fig. S68†), in accord with the proposal that ZopL9 is implicated in the ring contraction in the zopfiellin pathway of *D. curvata*, by first hydroxylating deoxyscytalidin to give **19**, then catalysing the ring contraction to give the octadride core.

**Scheme 3:**
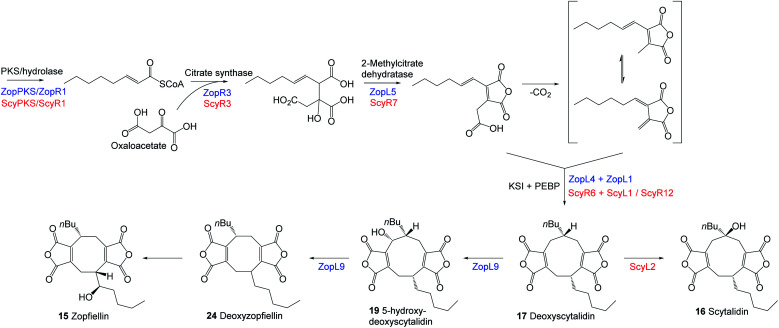
Proposed biosynthetic pathways of the maleidrides deoxyscytalidin **17**, scytalidin **16** and zopfiellin **15**.

Having identified **17**, **19** and **24** as intermediates in the zopfiellin pathway – through both feeding studies and *in vitro* assays – an extract of wild-type *D. curvata* was re-analysed, which revealed the presence of all three compounds as minor metabolites (Fig. S25†). Taken together with the bioinformatic analysis and the discovery that ScyL2 catalyses the final step of the scytalidin pathway, a branching biosynthetic route for the production of deoxyscytalidin, scytalidin and zopfiellin can now be proposed as shown in Scheme 3.

Intriguingly there is a homologue to ZopL9 (PhiK) encoded by the phomoidride BGC (44.2% homology – Fig. S11†). The structure of the phomoidrides, *e.g.***5**, suggests that multiple oxidations must occur during their biosynthesis, but no ring contraction of the nine-membered core occurs in this pathway, and the role of PhiK has not yet been investigated.

## Conclusions

This work categorically establishes the biosynthetic relationship between the nonadride scytalidin **16** and the octadride zopfiellin **15**, *via* the shared nonadride intermediate; deoxyscytalidin **17**. In both pathways, deoxyscytalidin is the substrate for αKG-dependent dioxygenase enzymes. In the scytalidin pathway ScyL2 catalyses a 6-hydroxylation of deoxyscytalidin to produce scytalidin whereas in the zopfiellin pathway ZopL9 catalyses a 5-hydroxylation of deoxyscytalidin and is implicated in a ring contraction to produce the octadride core. Interestingly, when cultures of *D. curvata* Δ*zopPKS* were fed with scytalidin, (possessing a 6-hydroxy group), 5-hydroxylation occurred to give castaneiolide **6** and two novel ring-cleaved maleidrides **20** and **21** were produced. Genes encoding αKG-dependent enzymes have been identified within a further three maleidride BGCs; namely *rbtB*, *rbtE*, *rbtG* and *rbtU* of the rubratoxin cluster, and the currently uncharacterised *phiK* of the phomoidride BGC and *pvL5* of the cornexistin BGC. This highlights the important role these, often multifunctional, enzymes play in generating the structural diversity seen in the maleidride class of natural products. Intriguing questions which remain to be answered regarding the biosynthesis of zopfiellin include the mechanism by which the ring contraction occurs, and the identification of the enzyme responsible for installing the hydroxyl group at C-1′′ of the side chain of zopfiellin. ZopR4, which is uniquely encoded by the zopfiellin BGC and predicted to be a FAD-dependent oxidoreductase, is a candidate for catalysing such a transformation, but this remains to be explored experimentally. Additionally, the roles of the isochorismatase-like enzyme (ZopL2/ScyR4), the enoyl CoA hydratase (ZopR2/ScyR2) and the small hypothetical protein (ZopL8/ScyR11), which are encoded by both BGCs, are currently unknown.

## Conflicts of interest

There are no conflicts to declare.

## Supplementary Material

SC-011-D0SC04309E-s001

SC-011-D0SC04309E-s002
