# Supplementary material for: Uncovering biosynthetic relationships between antifungal nonadrides and octadrides
Source: Chem Sci. 2020 Oct 7;11(42):11570–8. doi: 10.1039/d0sc04309e (PMC8162798; doi:10.1039/d0sc04309e)
Supplement: SC-011-D0SC04309E-s001 [file SC-011-D0SC04309E-s001.pdf]

Electronic Supplementary Material (ESI) for Chemical Science.
This journal is © The Royal Society of Chemistry 2020

Experimental and Supporting Information for:

# Uncovering Biosynthetic Relationships Between Antifungal Nonadrides and Octadrides

Kate M. J. de Mattos-Shipley,[‡*a,b] Catherine E. Spencer,[‡a] Claudio Greco,[a] David M. Heard,[a] Daniel E. O'Flynn,[a] Trong T. Dao,[a] Zhongshu Song,[a] Nicholas P. Mulholland,[c] Jason L. Vincent,[c] Thomas J. Simpson,[a] Russell J. Cox,[d] Andrew M. Bailey*[a] and Christine L. Willis*[a]

a. *School of Chemistry, University of Bristol, Cantock's Close, Bristol, UK, BS8 1TS*
b. *School of Biological Sciences, University of Bristol, 24 Tyndall Avenue, Bristol, BS8 1TQ, UK.*
c. *Syngenta, Jealott's Hill International Research Centre, Bracknell, RG42 6EY, UK*
d. *Institute for Organic Chemistry and BMWZ, Leibniz University of Hannover, Schneiderberg 38, 30167, Hannover, Germany.*

‡ These authors contributed equally
* corresponding authors: Chris.Willis@bristol.ac.uk, Andy.Bailey@bristol.ac.uk, Kate.deMattos-Shipley@bristol.ac.uk

## Table of Contents

**General Procedures** ........ **3**
**NMR** ........ **3**
**Optical rotation data collection** ........ **3**
**HRMS** ........ **3**
**LCMS** ........ **3**
Analytical LCMS ........ 3
Preparative LCMS ........ 3
**Strains** ........ **4**
**Growth and Fermentation Conditions** ........ **4**
**Fungal Nucleic Acid Preparation** ........ **4**
**Genome Sequencing, Transcriptomics and Bioinformatics** ........ **4**
**Gene Disruption Procedures** ........ **13**
**Genetic Characterisation of Knockout Strains** ........ **13**
**Primer Table** ........ **14**
**Transformation Protocols** ........ **15**
*S. cerevisiae* transformation ........ 15
*D. curvata* transformation ........ 15
*S. album* transformation ........ 15
**Metabolite Extraction of *D. curvata*** ........ **16**
**Metabolite Extraction of *S. album*** ........ **16**
**HPLC Data** ........ **17**
**Isolated metabolites** ........ **21**
NMR data for castaneiolide **6** ........ 22
NMR data for zopfiellin **15** ........ 26
NMR data for scytalidin **16** ........ 30
NMR data for deoxyscytalidin **17** ........ 32
NMR data for 5-hydroxy-deoxyscytalidin **19** ........ 36
NMR data for **20** and **21** ........ 40
NMR data for deoxyzopfiellin **24** ........ 48
***In vitro* assays with ZopL9** ........ **50**
**Labelling studies** ........ **52**
*S. album* UAMH 3620 $^{13}$C labelling ........ 52
Zopfiellin $^{13}$C labelling ........ 53
**Feeding Compounds to *Diffractella curvata* Strain Δ*zopPKS*** ........ **56**
**Synthesis** ........ **56**
**X-Ray Crystallography** ........ **62**
**Assignment of zopfiellin stereochemistry** ........ **63**
**References** ........ **65**

## Supplementary Tables

**Table S1**: Predicted functions for genes located within the zopfiellin gene cluster. ....3
**Table S2**: Predicted functions for genes located within the scytalidin gene cluster ....4
**Table S3**: Homologues within the zopfiellin and scytalidin gene clusters. ....5
**Table S4**: Primers used in this study. ....12
**Table S5**: NMR assignment for castaneiolide **6** ([a]500 MHz, [b]125 MHz in acetone-$d_6$) ....20
**Table S6**: NMR assignments for zopfiellin **15** ([a]500 MHz, [b]125 MHz in $CDCl_3$) ....24
**Table S7**: NMR assignments for scytalidin **16** ([a]500 MHz, [b]125 MHz in $CD_2Cl_2$) ....28
**Table S8**: NMR assignments for deoxyscytalidin **17** ([a]500 MHz, [b]125 MHz in $CDCl_3$) ....29
**Table S9**: NMR assignments for 5-hydroxy-deoxyscytalidin **19** ([a]500 MHz, [b]125 MHz in $CDCl_3$) ....34
**Table S10**: NMR assignments for **20** and **21** ([a]500 MHz, [b]125 MHz in $CDCl_3$). ....38
**Table S11**: NMR assignments for deoxyzopfiellin **24** ([a]500 MHz, [b]125 MHz in pyridine-$d_5$).... 46
**Table S12**: Crystal data and structure refinement for **16**, **17** and **18** ....60

## Supplementary Figures

**Figure S1**: RNAseq data mapped to the zopfiellin biosynthetic gene cluster ....3
**Figure S2**: Relative RPKM (Reads Per Kilobase of transcript per Million mapped reads) ....3
**Figure S3**: An ACT comparison of the zopfiellin and scytalidin BCGs....5
**Figure S4**: A phylogenetic analysis of the KSI-like proteins ....6
**Figure S5**: A Percent Identity Matrix for various maleidride KSI-like proteins ....7
**Figure S6**: A phylogenetic analysis of the PEBP-like proteins ....7
**Figure S7**: A Percent Identity Matrix for various maleidride PEBP-like proteins ....7
**Figure S8**: A phylogenetic analysis of various α-ketoglutarate dependant dioxygenases ....8
**Figure S9**: A Percent Identity Matrix for various α-ketoglutarate dependant dioxygenases ....8
**Figure S10**: Alignment of ScyL2 and ZopL9 with various alpha-ketoglutarate dependant dioxygenases....9
**Figure S11**: An alignment of ZopL9 and PhiK ....10
**Figure S12**: The bipartite approach to gene disruptions. ....11
**Figure S13**: PCR used to confirm correct integration for gene disruption....12
**Figure S14**. HPLC analysis of crude extracts from various *D. curvata* liquid cultures ....15
**Figure S15**. Timecourse for the production of zopfiellin **15** ....15
**Figure S16**. LCMS analysis of zopfiellin **15** ....16
**Figure S17**: HPLC analysis of crude extracts from a range of *S. album* UAMH 3620 liquid cultures....16
**Figure S18**: LCMS analysis of scytalidin **16**: A, UV spectrum; B, $ES^-$ spectrum. ....16
**Figure S19**: LCMS analysis of deoxyscytalidin **17**: A, UV spectrum; B, $ES^-$ spectrum. ....17
**Figure S20**: LCMS analysis of 5-hydroxy-deoxyscytalidin **19**: A, UV spectrum; B, $ES^-$ spectrum. ....17
**Figure S21**: LCMS analysis of castaneolide **6**: A, UV spectrum; B, $ES^-$ spectrum. ....17
**Figure S22**: LCMS analysis of compound **20**: A, UV spectrum; B, $ES^-$ spectrum. ....18
**Figure S23**: LCMS analysis of compound **21**: A, UV spectrum; B, $ES^-$ spectrum. ....18
**Figure S24**: LCMS analysis of compound **24**: A, UV spectrum; B, $ES^-$ spectrum. ....18
**Figure S25**: HPLC analysis of a crude extract from a wild-type *D. curvata* PDB liquid culture ....19
**Figure S26**: $^1H$ NMR spectrum (500 MHz, Acetone-$d_6$) of **6**. ....21
**Figure S27**: $^{13}C$ NMR spectrum (125 MHz, Acetone-$d_6$) of **6**. ....21
**Figure S28**: COSY spectrum of 6 (acetone-$d_6$) ....22
**Figure S29**: HMBC spectrum of **6** (acetone-$d_6$) ....22
**Figure S30**: HSQC spectrum of **6** (acetone-$d_6$) ....23
**Figure S31**: $^1H$ NMR spectrum (500 MHz, $CDCl_3$) of **15**. ....25
**Figure S32**: $^{13}C$ NMR spectrum (125 MHz, $CDCl_3$) of **15**. ....25
**Figure S33**: COSY spectrum of **15**. ....26
**Figure S34**: HSQC spectrum of **15**. ....26

**Figure S35**: HMBC spectrum of **15**. ....27
**Figure S36**: Key HMBC correlations for zopfiellin **15** ....27
**Figure S37**: $^1$H NMR spectrum (500 MHz, $CD_2Cl_2$) of **16**. ....29
**Figure S38**: $^{13}$C NMR spectrum (125 MHz, $CD_2Cl_2$) of **16**. ....29
**Figure S39**: $^1$H NMR spectrum (500 MHz, $CDCl_3$) of **17**. ....31
**Figure S40**: $^{13}$C NMR spectrum (125 MHz, $CDCl_3$) of **17**. ....31
**Figure S41**: COSY spectrum of **17**. ....32
**Figure S42:** HSQC spectrum of **17**. ....32
**Figure S43:** HMBC spectrum of **17**. ....33
**Figure S44:** $^1$H NMR spectrum (500 MHz, $CDCl_3$) of **19**. ....35
**Figure S45:** $^{13}$C NMR spectrum (125 MHz, $CDCl_3$) of **19**. ....35
**Figure S46:** COSY spectrum of **19**. ....36
**Figure S47:** HSQC spectrum of **19**. ....36
**Figure S48:** HMBC spectrum of **19**. ....37
**Figure S49**: NOESY spectrum **19**. ....37
**Figure S50**: $^1$H NMR spectrum (500 MHz, $CDCl_3$) of **20**. ....39
**Figure S51:** $^{13}$C NMR spectrum (125 MHz, $CDCl_3$) of **20**. ....39
**Figure S52**: COSY spectrum of **20**. ....40
**Figure S53**: Key COSY correlations of **20**. ....40
**Figure S54**: HSQC spectrum of **20**. ....41
**Figure S55**: HMBC spectrum of **20**. ....41
**Figure S56**: Key HMBC correlations for **20**. ....42
**Figure S57:** $^1$H NMR spectrum (500 MHz, $CDCl_3$) of **21**. ....42
**Figure S58:** $^{13}$C NMR spectrum (125 MHz, $CDCl_3$) of **21**. ....43
**Figure S59:** COSY spectrum of **21**. ....43
**Figure S60**: Key COSY correlations of **21**. ....44
**Figure S61:** HSQC spectrum of **21**. ....44
**Figure S62**: HMBC spectrum of **21**. ....45
**Figure S63**: HMBC correlations of **21**. ....45
**Figure S64**: $^1$H NMR spectrum (500 MHz, $CDCl_3$) of **24**. ....46
**Figure S65**: $^{13}$C NMR spectrum (125 MHz, $CDCl_3$) of **24**. ....47
**Figure S66**: HSQC spectrum of **24**. ....47
**Figure S67**: *zopL9* codon optimised for expression in *E. coli* ....48
**Figure S68**: *In vitro* assays using purified ZopL9 ....49
**Figure S69**: $^{13}$C NMR of unlabelled and labelled deoxyscytalidin **17** from [1,2-$^{13}C_2$] acetate (125 MHz, $CDCl_3$)...50
**Figure S70**: $^{13}$C NMR of unlabelled and labelled deoxyscytalidin **17** from [1,2-$^{13}C_2$] acetate (125 MHz, $CDCl_3$)...51
**Figure S71**: $^{13}$C NMR of unlabelled and labelled zopfiellin **15** from [1,2-$^{13}C_2$] acetate (125 MHz, $CDCl_3$) ....52
**Figure S72:** $^{13}$C NMR of unlabelled and labelled zopfiellin **15** from [1,2-$^{13}C_2$] acetate (125 MHz, $CDCl_3$) ....52
**Figure S73**: $^{13}$C NMR of unlabelled and labelled zopfiellin **15** from [1,2-$^{13}C_2$] acetate (125 MHz, $CDCl_3$) ....53
**Figure S74**: $^{13}$C NMR of unlabelled and labelled zopfiellin **15** from [1,2-$^{13}C_2$] acetate (125 MHz, $CDCl_3$) ....53
**Figure S75**: $^1$H NMR spectrum (400 MHz, $CDCl_3$) of **18**. ....55
**Figure S76:** $^{13}$C NMR spectrum (100 MHz, $CDCl_3$) of **18**. ....55
**Figure S77:** $^1$H NMR spectrum (100 MHz, $CDCl_3$) of ***R*-MTPA-zopfiellin** ....57
**Figure S78**: $^{13}$C NMR spectrum (100 MHz, $CDCl_3$) of ***R*-MTPA-zopfiellin** ....57
**Figure S79**: $^1$H NMR spectrum (100 MHz, $CDCl_3$) of ***S*-MTPA-zopfiellin** ....59
**Figure S80**: $^{13}$C NMR spectrum (100 MHz, $CDCl_3$) of ***S*-MTPA-zopfiellin** ....59
**Figure S81:** Comparison of $^1$H NMR (400 MHz, $CDCl_3$) of (S)-MTPA-zopfiellin and (R)-MTPA-zopfiellin ....61
**Figure S82**: Representation of the spectroscopically dominant conformation in Mosher's ester analysis. ....62
**Figure S83**: Mosher's ester analysis results viewed in the syn-co-planar conformation model. ....62

## General Procedures

Molecular biology kits were used according to manufacturer's protocols. PCR was carried out using KAPA-Hifi (Roche) or BioMix Red (Bioline). Restriction endonucleases were purchased from Thermo Fisher Scientific.

## NMR

Instruments used; Varian 400-MR (400MHz), Varian VNMRS500 (500MHz), Bruker 500 Cryo (500MHz) or Varian VNMRS600 Cryo (600MHz). Chemical shifts (δ) quoted in parts per million (ppm) and coupling constants (*J*) in Hertz (Hz), rounded to 0.5 Hz intervals. Two-dimensional NMR techniques (HSQC, COSY, HMBC) were used routinely for the assignment of structures. Use of NOESY and TOCSY techniques is indicated as appropriate, and the identified correlations tabulated (Tables S5-10) Residual solvent peaks were used as the internal reference for proton and carbon chemical shifts.

## Optical rotation data collection

Specific rotations ([a]DT) were measured on a Bellingham and Stanley Ltd. ADP220 polarimeter and are quoted in (° ml) (g dm)$^{-1}$.

## HRMS

(1) Bruker Daltonics micrOTOF II, (2) Bruker Daltonics Apex IV FT-ICR instruments.

## LCMS

### Analytical LCMS

All crude extracts were prepared to a concentration of 10 mg/ml in HPLC grade acetonitrile and placed in LCMS vials. 20 µl of the extracts were injected and analysed using Waters 2445 SFO HPLC; Waters 2767 autosampler, Phenomenex Kinetex column (2.6 µ, C18, 100 Å, 4.6 × 100 mm) equipped with a Phenomenex Security Guard precolumn (Luna C5 300 Å) eluted at 1 mL/min. Detection was achieved by Waters 2998 diode array detector between 200 and 400 nm; Waters Quattro Micro ESI mass spectrometer in ES+ and ES- modes between 100 *m/z* and 1000 *m/z*; Waters 2424 ELS detector. Solvents were: A, HPLC grade $H_2O$ containing 0.05% formic acid; B, HPLC grade MeOH containing 0.045% formic acid; and C, HPLC grade $CH_3CN$ containing 0.045% formic acid. Samples were run on a 5 – 95 % $CH_3CN$ gradient over 20 minutes comprising: 0 min, 95 % A; 1 min, 95% A; 2 min, 60 % A; 15 min, 5% A; 17 min, 5% A; 18 min, 95% A; 20 min, 95% A; flow rate 1 mL·min-1.

### Preparative LCMS

Compounds were purified using a Waters time-directed autopurification system compromising Waters 2767 autosampler, Waters 2545 pump system, Phenomenex Kinetex column (5 µ, $C_{18}$, 100 Å, 250 × 21.20 mm) equipped with Phenomenex Security Guard precolumn (Luna $C_5$ 300 Å) eluted at 16 mL/min. Solvents were: A, HPLC grade $H_2O$ containing 0.05% formic acid; B, HPLC grade MeOH containing 0.045% formic acid; and C, HPLC grade $CH_3CN$ containing 0.045% formic acid. The post column was split (100:1) with the minority flow made up with HPLC grade MeOH containing formic acid 0.045% to 1 mL/min for simultaneous analysis by Waters 2998 diode array detector between 200 and 400 nm; Waters Quattro Micro ESI mass spectrometer in $ES^+$ and $ES^-$ modes between 100 *m/z* and 1000 *m/z*; Waters 2424 ELS detector. Metabolites were collected into glass test tubes. Combined samples were evaporated under $N_2$ gas, weighed and dissolved in a suitable solvent for future applications.

## Strains

*Diffractella curvata* CBS 591.74 was obtained from the CBS collection; *Scytalidium album* strains UAMH 3611 and UAMH 3620 from the University of Alberta Mold Herbarium and culture collection; *Saccharomyces cerevisiae* (Stratagene) strain YPH499 was used for plasmid assembly by yeast homologous recombination. Escherichia coli strain TOP10 (Invitrogen) was used as a host for all plasmids.

## Growth and Fermentation Conditions

*D. curvata* was maintained on PDA at 25 °C. Agar plugs were used to inoculate 100 mL PDB in 500 mL non-baffled Erlenmeyer flasks and grown at 25 °C with shaking at 200 rpm. After growing for 1 week, the seed culture was homogenised and used to inoculate flasks containing fresh PDB. For metabolite production the cultures were grown for 8 days. The homogenised *D. curvata* seed culture was also used to inoculated static rice cultures (50 g rice and 50 ml water autoclaved in 500 mL Erlenmeyer flasks) which were grown for 21 – 28 days at 25 °C.

*S. album* was maintained on MEA at 25 °C. Agar plugs were used to inoculate 100 mL GN in 500 mL non-baffled Erlenmeyer flasks and grown at 25 °C with shaking at 200 rpm. After growing for 1 week, the seed culture was homogenised and used to inoculate flasks containing MEB. For metabolite production the cultures were grown for 12 days. The homogenised *S. album* seed culture was also used to inoculate static rice- or oat-based cultures (50 g rice/oats and 50 ml of water autoclaved in 500 mL Erlenmeyer flasks) which were grown for 21 – 28 days at 25 °C.

## Fungal Nucleic Acid Preparation

Fungi were grown in an appropriate liquid medium, freeze dried and then ground under liquid nitrogen. Genomic DNA was prepared using the GenElute Plant Genomic DNA miniprep kit (Sigma). RNA was prepared using the RNeasy Plant Mini Kit (Qiagen) and DNA contamination was removed using the Qiagen RNase-free DNase set.

## Genome Sequencing, Transcriptomics and Bioinformatics

Genome sequencing and assembly was conducted by the Cambridge University DNA Sequencing Facility. *D. curvata* was sequenced using Nextera shotgun sequencing on a MiSeq platform and an assembly was generated using Newbler v2.9[1] to give a genome of *ca* 44.79 Mb, contained on 279 scaffolds with an $N_{50}$ of 319 Kb. *S. album* was sequenced using a combination of Nextera mate-pair and shotgun sequencing using a MiSeq platform and assembled using Newbler v2.9[1] to give a genome of 47.6 Mb over 19 scaffolds with a $N_{50}$ of *ca* 8.29 Mb. An initial screen of the genome data for *D. curvata* and *S. album*, searching for homologues to the *B. fulva* byssochlamic acid gene cluster, revealed putative maleidride gene clusters within both genomes. In the *D. curvata* genome this gene cluster is located on scaffold 92, position 41603 – 78875 nt. The *S. album* maleidride biosynthetic gene cluster (BGC) is located on scaffold 3, position 3125884 – 3167631 nt.

RNA sequencing was also conducted by the Cambridge University DNA Sequencing Facility. Paired-end Illumina sequencing was conducted after TruSeq RNAseq library preparation. The sequencing reads were mapped to the genome assembly using Tophat.[2]

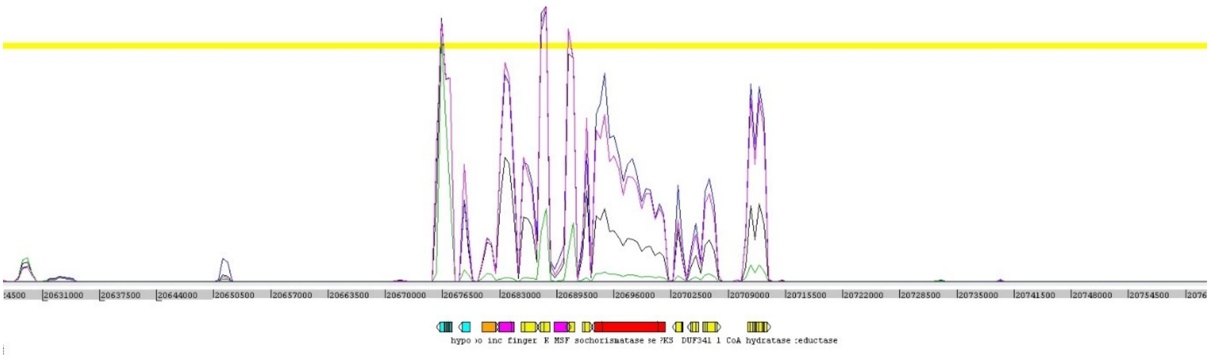

**Figure S1**: RNAseq data generated from four different culture conditions, mapped to the zopfiellin biosynthetic gene cluster. Clear coregulation of the cluster can be seen, within a relatively un-transcribed region of the genome. See Figure S2 for RPKM data.

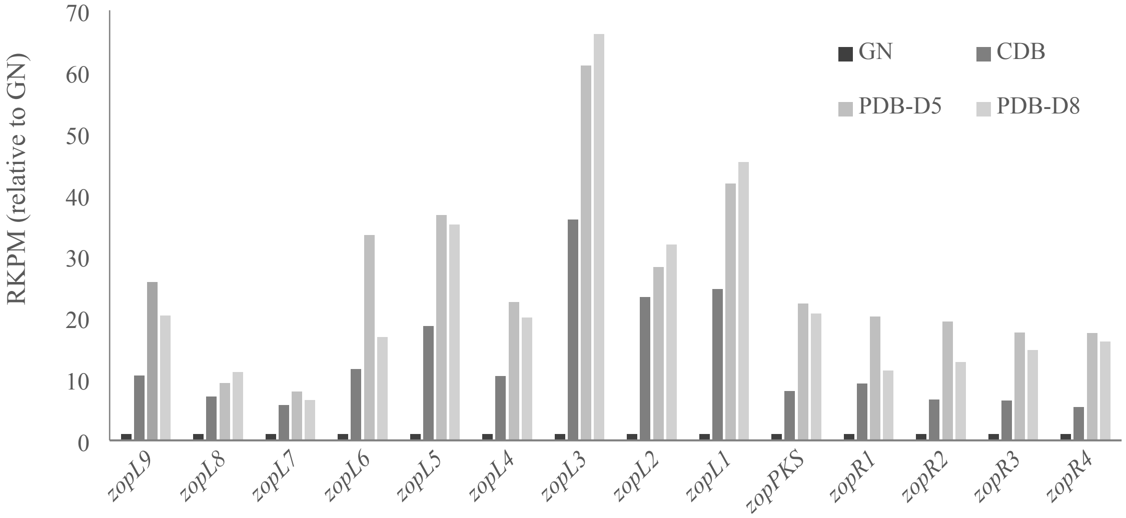


**Figure S2**: Relative RPKM (Reads Per Kilobase of transcript per Million mapped reads) values demonstrating clear co-regulation within the zopfiellin BGC under non-production (GN), medium yielding (GN), and high yielding (PDB days 5 and 8 post-inoculation) production conditions.

**Table S1**: Predicted functions for genes located within the zopfiellin gene cluster. Homologues were identified by searching the Swissprot database using each predicted protein sequence as a query (BLASTp). Domains were identified using InterPro. The domain identifiers for zopPKS are: KS; IPR020841. AT; IPR020801. DH; IPR020807. CMeT*; IPR029063. ER; IPR020843. KR; IPR013968. ACP; IPR020806. * denotes instances where homologues were identified within the NCBI nr (nonredundant), rather than Swissprot, database.

| **Gene** | **Putative function** | **Homologue** | **Identity (z/s)** | **E value (z/s)** | **Query coverage (z/s)** | **Score (z/s)** | **Domains** |
|---|---|---|---|---|---|---|---|
| *zopL9* | Hydroxylase/Desaturase | GA4[3] | 29% | $3e^{-22}$ | 82% | 97.4 | - |
| | | asaB[4] | 36% | $2e^{-14}$ | 40% | 73.9 | |
| *zopL8* | Hypothetical protein | - | - | - | - | - | - |
| *zopL7* | Transcription factor (TF) | alnR[5] | 24.4% | $4e^{-23}$ | 95% | 104 | Zn(2)-C6 fungal-type DNA-binding domain (IPR001138) |
| *zopL6* | Major facilitator superfamily (MFS) transporter | Itp1[6] | 35% | $3e^{-8}$ | 91% | 270 | Major facilitator superfamily domain (IPR020846) |
| *zopL5* | 2-methylcitrate dehydratase (2MCDH) | PrpD[7] | 45% | $3e^{-140}$ | 97% | 415 | 2-methylcitrate dehydratase PrpD (IPR012705) |

| | | | | | | | |
|---|---|---|---|---|---|---|---|
| *zopL4* | Ketosteroid isomerase-like protein (KI) | BfL6[8]* | 61% | $2e^{-60}$ | 54% | 201 | NTF2 (IPR002075) |
| *zopL3* | Major facilitator superfamily (MFS) transporter | Itp1[6] | 37% | $2e^{-85}$ | 94% | 274 | Major facilitator superfamily domain (IPR020846) |
| *zopL2* | Isochorismatase-like hydrolase / amidohydrolase | NicR[9] | 29% | $1e^{-16}$ | 98% | 77 | Isochorismatase-like (IPR000868) |
| *zopL1* | Phosphatidylethanolamine-binding protein (PEBP) | Tfl1[10] | 31% | $2e^{-12}$ | 78% | 65.9 | Phosphatidylethanolamine -binding protein (IPR008914) |
| *zopPKS* | hrPKS | Tox1-PKS[11] | 45% | 0.0 | 98% | 2125 | KS, AT, DH, *C*MeT, ER, KR, ACP. See caption. |
| *zopR1* | DUF341 family hydrolase / esterase | Fub4[12] | 33% | $4e^{-15}$ | 87% | 75.1 | Serine hydrolase FSH (IPR005645) |
| *zopR2* | Enoyl-CoA isomerase | Ech1[13] | 28% | $7e^{-7}$ | 48% | 52.4 | Enoyl-CoA hydratase/isomerase (IPR001753) |
| *zopR3* | Citrate synthase (CS) | CshA[14] | 36% | $4e^{-78}$ | 96% | 254 | Citrate synthase (IPR002020) |
| *zopR4* | FAD-dependent oxidoreductase | YanF[15] | 28% | $3e^{-29}$ | 84% | 124 | FAD-binding domain, PCMH-type (IPR016166) |
| | | sol5[16, 17] | 28% | $1e^{-28}$ | 93% | 121 | |

**Table S2**: Predicted functions for genes located within the scytalidin gene cluster. Homologues were identified by searching the Swissprot database using each predicted protein sequence as a query (BLASTp). Domains were identified using InterPro. The domain identifiers for scyPKS are: KS; IPR020841. AT; IPR020801. DH; IPR020807. CMeT*; IPR029063. ER; IPR020843. KR; IPR013968. ACP; IPR020806. * denotes instances where homologues were identified within the NCBI nr (nonredundant) database rather than the Swissprot database.

| Gene | Putative function | Homologue | Identity (z/s) | E value (z/s) | Query coverage (z/s) | Score (z/s) | Domains |
|---|---|---|---|---|---|---|---|
| *scyL2* | Hydroxylase | aclN[18] | 33.8% | $8e^{-22}$ | 46% | 94.4 | - |
| | | mfR1[19] | 25% | $1e^{-14}$ | 85% | 75.9 | |
| *scyL1* | Phosphatidylethanolamine -binding protein (PEBP) | bfL5[8]* | 41.3% | $2e^{-33}$ | 85% | 129 | Phosphatidylethanolamine -binding protein (IPR008914) |
| *scyPKS* | hrPKS | Tox1-PKS[11] | 43.2% | 0.0 | 98% | 1845 | KS, AT, DH, CMeT, ER, KR, ACP. |
| *scyR1* | DUF341 Hydrolase | Fub4[12] | 25.5% | $2e^{-12}$ | 94% | 67 | Serine hydrolase FSH (IPR005645) |
| *scyR2* | Enoyl CoA Hydratase / Isomerase | Ech1[13] | 26.7% | $3e^{-17}$ | 95% | 82 | Enoyl-CoA hydratase/isomerase (IPR001753) |
| *scyR3* | Citrate Synthase | mfR3[20] | 38.3% | $8e^{-92}$ | 94% | 288 | Citrate synthase (IPR002020) |
| *scyR4* | Isochorismatase-like hydrolase / amidohydrolase | CSHase[21] | 28.3% | $3e^{-13}$ | 94% | 69.3 | Isochorismatase-like (IPR000868) |
| *scyR5* | MFS Transporter | mfM6[20] | 40.6% | $2e^{-135}$ | 98% | 405 | Major facilitator superfamily domain (IPR020846) |
| *scyR6* | Ketosteroid-like protein (KSI) | bfL6[8]* | 51.1% | $9e^{-62}$ | 71% | 203 | NTF2 (IPR002075) |
| *scyR7* | 2-Methylcitrate Dehydratase | prpD[7] | 44.2% | $8e^{-132}$ | 97% | 393 | 2-methylcitrate dehydratase PrpD (IPR012705) |
| *scyR8* | Transcription factor | alnR[5] | 26.7% | $4e^{-12}$ | 38% | 72 | Zn(2)-C6 fungal-type DNA- |

| | | | | | | | |
|---|---|---|---|---|---|---|---|
| | | | | | | | binding domain (IPR001138) |
| *scyR9* | Histone H2A | H2A.Z[22] | 95.1% | $2e^{-81}$ | 86% | 239 | Histone H2A (IPR002119) |
| *scyR10* | MFS Transporter | mfR5[20] | 42% | $1e^{-130}$ | 88% | 394 | Major facilitator superfamily domain (IPR020846) |
| *scyR11* | Hypothetical Protein | - | - | - | - | - | - |
| *scyR12* | Phosphatidylethanolamine -binding protein (PEBP) | bfL9[8]* | 44.5% | $1e^{-54}$ | 99% | 172 | Phosphatidylethanolamine -binding protein (IPR008914) |

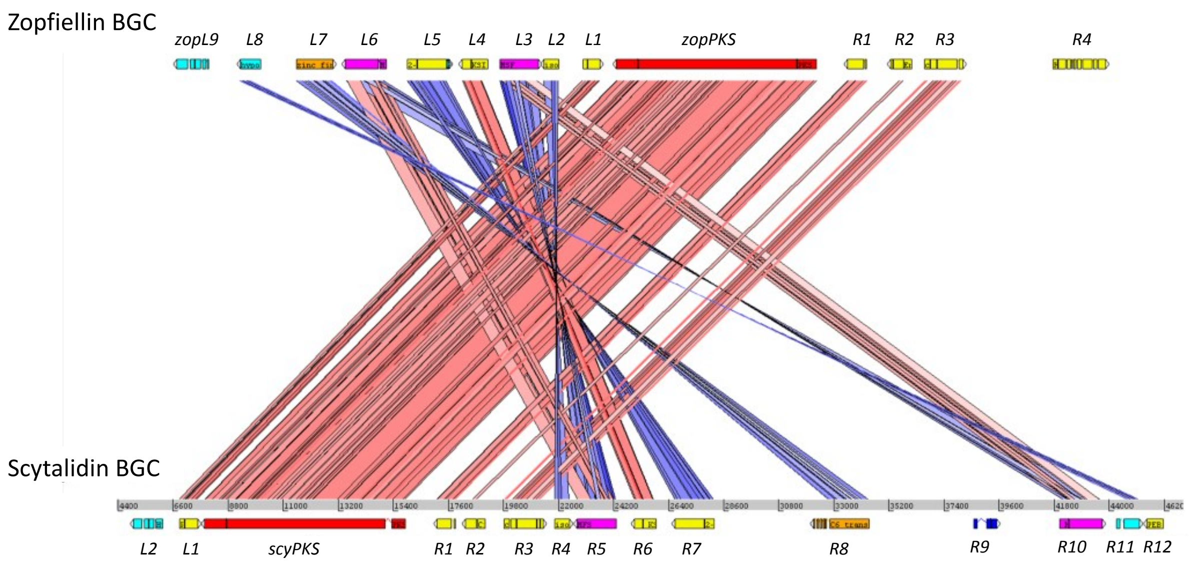


**Figure S3**: An ACT comparison of the zopfiellin and scytalidin BCGs identified likely homologous genes and highlighted the similarity between the two biosynthetic gene clusters.

**Table S3**: Homologues within the zopfiellin and scytalidin gene clusters.

| Zopfiellin BCG | Scytalidin BCG | Putative function | % identity (protein) |
|---|---|---|---|
| zopL9 | - | Hydroxylase / desaturase | - |
| zopL8 | scyR11 | Hypothetical (incl. Dc_L8) | 53.5 |
| zopL7 | scyR8 | Transcription factor | 53.1 |
| zopL6 | scyR5 | MFS transporter | 80.4 |
| zopL5 | scyR7 | 2-methylcitrate dehydratase | 81.4 |
| zopL4 | scyR6 | KSI | 80.7 |
| zopL3 | scyR10 | MFS transporter | 38.5 |
| zopL2 | scyR4 | Isochorismatase | 70.3 |
| zopL1 | scyL1 | PEBP | 66.2 |
| zopPKS | scyPKS | PKS | 81.2 |
| zopR1 | scyR1 | DUF341 hydrolase | 73.1 |
| zopR2 | scyR2 | Enoyl-coA hydratase family | 53.5 |
| zopR3 | scyR3 | Citrate synthase | 54 |
| zopR4 | - | FAD-dependant oxidoreductase | - |
| - | scyL2 | Putative hydroxylase | - |
| - | scyR9 | Histone H2A* | - |

| | | |
|---|---|---|
| scyR12 | PEBP | - |

* ScyR9 belongs to the highly conserved H2A.F/Z family of histone H2A variants, which have been shown to play a role in transcriptional regulation and genomic stability.[23] As this protein is very highly conserved, it a reasonable to conclude that scyR9 plays no direct role in the biosynthesis of scytalidin, though it may indirectly impact production via a role in chromatin remodelling and transcriptional activation.

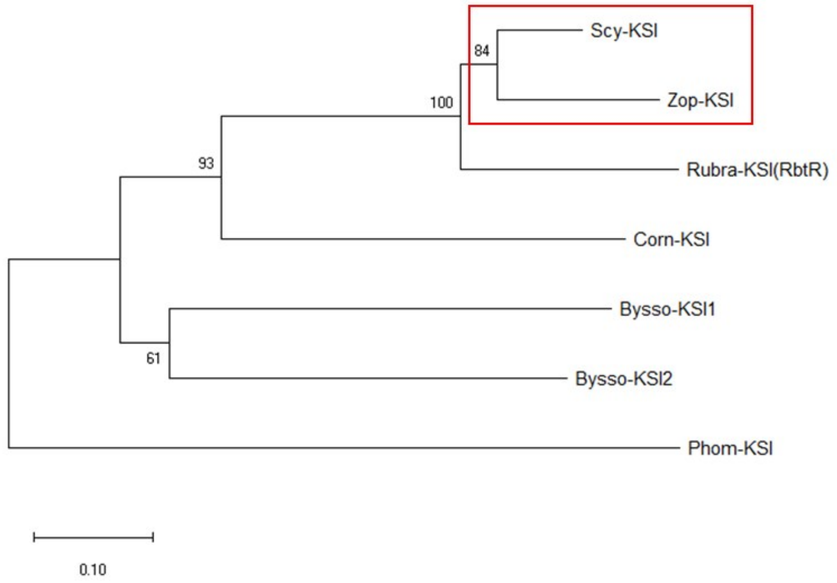


**Figure S4**: A phylogenetic analysis of the KSI-like proteins within published maleidride gene clusters places the scytalidin and zopfiellin KSIs within the same clade, demonstrating their relative similarity. The tree was produced in MEGA X[24] using the neighbour-joining method.[25]

| | | | | | | | |
|---|---|---|---|---|---|---|---|
| 1: Phom-KSI | 100.00 | 36.07 | 39.61 | 35.27 | 33.33 | 33.33 | 31.96 |
| 2: Bysso-KSI1 | 36.07 | 100.00 | 49.12 | 46.89 | 38.86 | 43.42 | 42.98 |
| 3: Bysso-KSI2 | 39.61 | 49.12 | 100.00 | 43.42 | 43.98 | 46.05 | 45.58 |
| 4: Corn-KSI | 35.27 | 46.89 | 43.42 | 100.00 | 51.05 | 52.10 | 52.94 |
| 5: Rubra-KSI | 33.33 | 38.86 | 43.98 | 51.05 | 100.00 | 76.74 | 69.85 |
| 6: Scy-KSI | 33.33 | 43.42 | 46.05 | 52.10 | 76.74 | 100.00 | 80.69 |
| 7: Zop-KSI | 31.96 | 42.98 | 45.58 | 52.94 | 69.85 | 80.69 | 100.00 |

**Figure S5**: A Percent Identity Matrix for various maleidride KSI-like proteins highlights the uniquely high homology between the KSI-like proteins encoded by genes in the zopfiellin and scytalidin BGCs, which share 80.69 % protein identity. Produced using Clustal Omega.

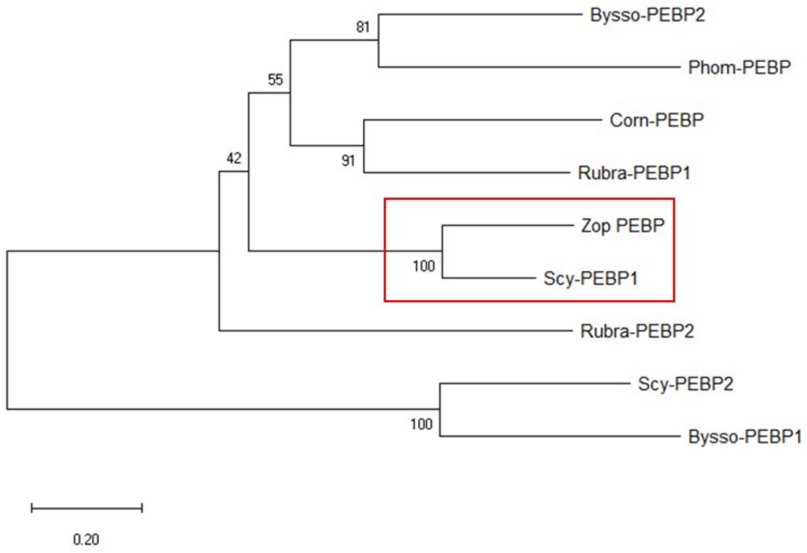


**Figure S6**: A phylogenetic analysis of the PEBP-like proteins within published maleidride gene clusters places the scytalidin and zopfiellin KSIs within the same clade, demonstrating their relative similarity. The tree was produced in MEGA X[24] using the neighbour-joining method.[25]

| | | | | | | | | | |
|---|---|---|---|---|---|---|---|---|---|
| 1: Scy-PEBP2 | 100.00 | 45.59 | 22.70 | 21.74 | 19.88 | 18.18 | 25.00 | 15.53 | 15.98 |
| 2: Bysso-PEBP1 | 45.59 | 100.00 | 20.69 | 19.63 | 19.02 | 18.93 | 18.32 | 14.81 | 16.57 |
| 3: Rubra-PEBP2 | 22.70 | 20.69 | 100.00 | 27.65 | 28.24 | 24.85 | 28.77 | 26.99 | 33.13 |
| 4: Zop_PEBP | 21.74 | 19.63 | 27.65 | 100.00 | 66.18 | 25.87 | 30.19 | 32.43 | 31.38 |
| 5: Scy-PEBP1 | 19.88 | 19.02 | 28.24 | 66.18 | 100.00 | 33.50 | 32.54 | 30.26 | 32.83 |
| 6: Phom-PEBP | 18.18 | 18.93 | 24.85 | 25.87 | 33.50 | 100.00 | 36.53 | 25.91 | 34.36 |
| 7: Bysso-PEBP2 | 25.00 | 18.32 | 28.77 | 30.19 | 32.54 | 36.53 | 100.00 | 39.78 | 41.57 |
| 8: Corn-PEBP | 15.53 | 14.81 | 26.99 | 32.43 | 30.26 | 25.91 | 39.78 | 100.00 | 43.06 |
| 9: Rubra-PEBP1 | 15.98 | 16.57 | 33.13 | 31.38 | 32.83 | 34.36 | 41.57 | 43.06 | 100.00 |

**Figure S7**: A Percent Identity Matrix for various maleidride PEBP-like proteins highlights the uniquely high homology between the PEBP-like proteins encoded by genes in the zopfiellin and scytalidin BGCs, which share 66.18 % protein identity. Produced using Clustal Omega.

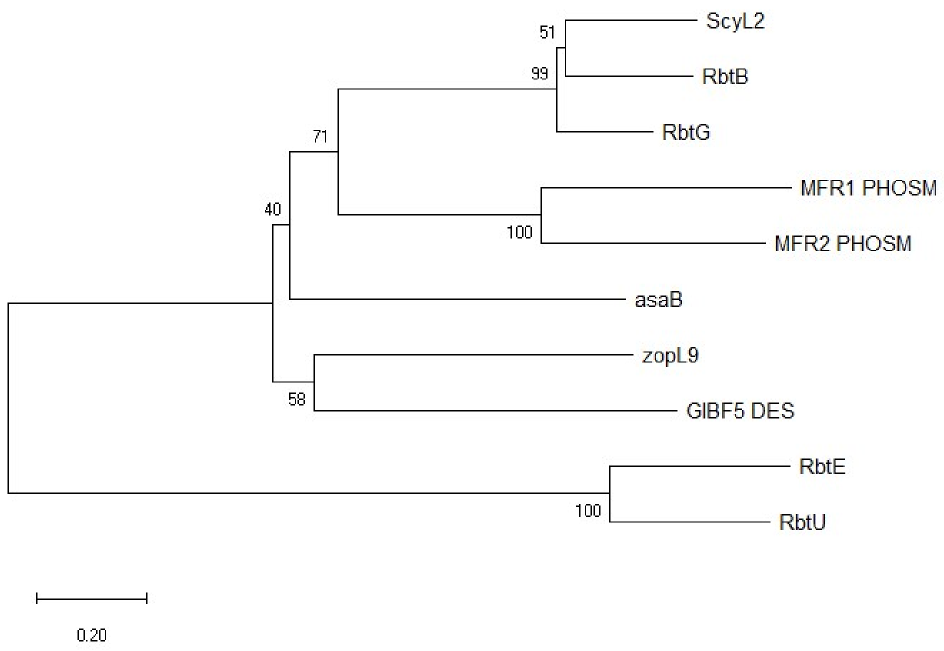


**Figure S8**: A phylogenetic analysis of various α-ketoglutarate dependant dioxygenases, demonstrating that ScyL2 clusters with RbtG and RbtB of the rubratoxin pathway, whereas zopL9 is most similar to DES, the desaturase from the gibberellin pathway.

| | | | | | | | |
|---|---|---|---|---|---|---|---|
| 1: MFR1_PHOSM | 100.00 | 41.38 | 24.81 | 25.38 | 26.62 | 19.92 | 19.41 |
| 2: MFR2_PHOSM | 41.38 | 100.00 | 24.23 | 23.28 | 22.99 | 23.83 | 22.39 |
| 3: ScyL2 | 24.81 | 24.23 | 100.00 | **63.79** | **61.17** | 25.10 | 24.91 |
| 4: RbtG | 25.38 | 23.28 | 63.79 | 100.00 | 65.75 | 29.23 | 26.49 |
| 5: RbtB | 26.62 | 22.99 | 61.17 | 65.75 | 100.00 | 27.03 | 27.44 |
| 6: zopL9 | 19.92 | 23.83 | 25.10 | 29.23 | 27.03 | 100.00 | **31.87** |
| 7: DES | 19.41 | 22.39 | 24.91 | 26.49 | 27.44 | 31.87 | 100.00 |

**Figure S9**: A Percent Identity Matrix for various α-ketoglutarate dependant dioxygenases, demonstrating that ScyL2 shares significant homology with RbtG and RbtB of the rubratoxin pathway, whereas ZopL9 is more unique, sharing most homology, albeit relatively low, with DES, the desaturase from the gibberellin pathway. Produced using Clustal Omega.

```
MFR1_PHOSM      -----MATAILPSTSGVIGLWDGTT--------DGKE----GFMDYA----NGDTNVKQP       39
MFR2_PHOSM      ---MATATTTLHSTTGTVYVADGTT--------DGKV----GYYNHT----DDSTNVI-R       40
ScyL2           ---MATETVTIH--QPKDALAKFTYLEWHDHYRTERPFQALDILHNAVDKREGNVSFKE-       54
RbtG            ---MATAKITTQ--HTYDIPAQLTYLEWHDHYETEKPFMVIRYPDDPPEMTGGNVTFKE-       54
RbtB            MATTTTLATTSK--NGGDVPAKLTYIEWHDHYETEEPHFVLNTPDDPPDAYAGNVTFKE-       57
zopL9           ---MATA-TVTTAP--TVVRTTADY---YD----APPVLKIH--TYTRESYEEQFGNKSV       45
DES.            ---MPHKDNLLESPVGKSVTATIAY---HS----G-PALPTS--PIAGVT-----TLQDC       42

MFR1_PHOSM      KEYEIQVHDIRKLDPQPTLLKNGYELVDIPTVVTDEQFIESGKSDEGNAYIKDVYFAECK       99
MFR2_PHOSM      KPIPIEVEDARTLSKSPTTKAEGYQLVNFHTKIPEEHFLNSK-LPENKELIEEVYFDECR       99
ScyL2           -GGKEVVHDVRGHEQDFTLDKHGFLFANAPTSLS-----PSDFQD--DEKIKEKYLPECE       106
RbtG            -GEEETIHDIRGHEDDFTLDGNGFLFTHAPTSLA-----PSDFLD--DEKIKTKYLPECE       106
RbtB            -GEEEIIHDIRGHEDKFTLDKQGFVFTKAPTSLS-----PSEFLD--EEKIKEKYLPECE       109
zopL9           IHHPINLKDIR--AANINLQNNGFQLIKLQSKLTNP----DDYLD--EETVKRVYIPELA       97
DES.            TQQAVAVTDIRPSVSSFTLDGNGFQVVKHTSAVGSPPYDHSSWTD--PVVRKEVYDPEII       100
                      : * *    . .   .*: . .  : :       .          :  *  *

MFR1_PHOSM      RIIEEVSGGVDLIIPVSFRMREQKGEKEST---------------------------TKK       132
MFR2_PHOSM      RLVQEVTGAAEA-YPYVYRVRNQEQNAKES------------------------------N       129
ScyL2           TYLKQYFDNVDQVHIIHYRVRCTN-SS---------------------------------       132
RbtG            AYLKGLLD-ADQVIIFHYRVRNTITSD---------------------------------       132
RbtB            KYYREYFKGIDEVVFIHYRARNSITAD---------------------------------       136
zopL9           EAVKKL-TGATEVRVLNPKVRDSS---------------------------------TEK       123
DES             ELAKSL-TGAKKVMILLASSRNVPFKEPELAPPYPMPGKSSSGSKEREAIPANELPTTRA       159
                   .                *

MFR1_PHOSM      ---LGNIESRYAPRPVAHLDRDTPTAITVLEE--------------------TVGKEKAQ       169
MFR2_PHOSM      ---KSNFH--TDFVPIVHVDRDDVTAPQRLRA--------------------SLGAEKAD       164
ScyL2           -----DPNSPTGPAKVVHVDQSGPHVTERIH---------------------KAFPDCAD       166
RbtG            -----DPYSDTGPARSAHVDLSSQTIRERIR---------------------NRYPDRAD       166
RbtB            -----DHNSPTGPARVAHVDLSGPEINARIR---------------------KAFPDRAD       170
zopL9           DGFENNWGKNNGAVRRIHIDLAPGGVEEALY---------------------PIFGEEYM       162
DES.            KGFQ--KGEEEGPVRKPHKDWGPSGAWNTLRNWSQELIDEAGDIIKAGDEAAKLPGGRAK       217
                                 * *         :

MFR1_PHOSM      ELLSKHKRWAQVNVWRPIGNPATMWPLCFLNHDRIPTWNYDTHVGHVWSLNDPRVSDRGQ       229
MFR2_PHOSM      MLLSKYKSYGSINVWRPVKNMVQKWPLMLVDHKSIEDWDYSTHMFTLHSSNDERVATRGA       224
ScyL2           --FLLRGHVRLINLWRPINGPIQNWPLAVCDANSLPEENLIE----TD---RIRKAQKGN       217
RbtG            --FLLSGRVRLINLWRPINGPIQNWPLAVCDGNTLPEKNLVL----TE---RIRTRDKAI       217
RbtB            --FILRGRVRLVNLWRPINGPLQNWPLCVADCNSIQEKHLVA----TK---RIRKTHQAV       221
zopL9           --KSIAGRWRLINAWKPTR-PVERDPLAVCDRVPD--EDLVP----LQ---RVVPGK-AL       209
DES.            --NYQGRRWALYTTWRPLK-TVKRDPMAYVDYWTADEEDGVS----FW---RNPPGVHGT       267
                            . *:*        *:   :       .                   .

MFR1_PHOSM      KTY-DCVVKHDDRYDYHYVSDLRPEECLVFCSFDSIP-------KYAMPHSAFWDNNVPA       281
MFR2_PHOSM      KEH-ETILTHDKRYRYIYASDMTPEEAWLFFAFHSDP-------ALGIPHGAFWDDSTKE       276
ScyL2           ----TRFVIQAPSMKWYYQSKMEDNTLLVFKSYES---QDG--VAKYASHCSFPLPTAGP       268
RbtG            ----ARFVVHSPAMKWYYQSGMEDGTLLVLKNYDSHAEEGG--VARYSAHCSFPLPTAGP       271
RbtB            ----TRLVVHEPTLKWYYQSGMEDDTLLVLKNYDS---EDG--VAKYVPHCSFSLPTATA       272
zopL9           MEQRYHLKTGKKDHDWYYASNQQPDEVLLFTQYSDFP---NRNTADRVPHVSVKLPGQ-E       265
DES.            FESDVLLTKANPKHKWYWISDQTPDEVLLMKIMDTESEKDGSEIAGGVHHCSFHLPGT-E       326
                      .        : : *        ::                   * :.

MFR1_PHOSM      DAPNRRSIEVRSLVFF-------------------------   297
MFR2_PHOSM      EALTRCSIEVRIWVFFD------------------------   293
ScyL2           MTPPRESIELRAFVFTYPRDEST------------------   291
RbtG            DTPPRESVEVRAVVLNYPRDDPSLVEPKPAISNVDSASITV   312
RbtB            STPPRESIEVRAFLFNYPRNEKSV-----------------   296
zopL9           DKPRRTSVDARCLVVW-------------------------   281
DES.            KEEVRESIETKFIAFW-------------------------   342
                    * *:: :   .
```

**Figure S10**: Alignment of ScyL2 and ZopL9 with various characterised alpha-ketoglutarate dependant dioxygenases. Although low overall homology is observed, all sequences contain the conserved residues thought to bind iron and α-ketoglutarate. The H-X-D/E-$X_n$-H motif is thought to be involved in binding $Fe^{2+}$ and an R-X-S motif that has been shown to bind 2-oxoglutarate.[26, 27]

```
zopL9       MATATVTTAPTVVRTTADYYDAPPVLKIHTYTRESYEEQFGNKSVIHHPINLKDIRAANI     60
phiK        -MPHSVEAEQGVVEAFLEYYAPNSDG--SLPDANDLEVQYGRKNLDLKSVKVGDMRSRDF     57
                :* :   **.:  :**             :. * *:*.*.:  : ::: *:*: ::

zopL9       NLQNNGFQLIKLQSKLTNPDDYLDEETVKRVYIPELAEAVKKLTGATEVRVLNPKVRDSS     120
phiK        KLEEDGFTLMKHESAM---TDFTDREKVKKEYYPEVAEAIKKHTGASKVICFNHNVRSTA     114
            :*:::** *:* :* :    *: *.*.**: * **:***:** ***::*  :* :**.::

zopL9       TEKDGFENNWGKNNGAVRRIHIDLAPGGVEEALYPIFGEEYMKSIAGRWRLINAWKPTRP     180
phiK        LPGLDLQKQKVDHVGPMRRVHIDVAPGGVSEAVTKRAGEEFMSKFQGRWKIMNAWKPIRT     174
                .::::  .: * :**:***:*****.**:    ***:*..: ***:::***** *

zopL9       VERDPLAV--CDRVPDEDLVPLQRVVPGKALMEQRYHLKTGKKDHDWYYASNQQPDEVLL     238
phiK        VERDPLGVAVAASVPDEDLINLKRYRPDGSLSESRYAVKAGE-GHQWYYVPQQRTDEMIL     233
            ******.*  .  ******: *:*  *. :* *.** :*:*: .*:***. :*: **::*

zopL9       FTQYSDFPNRNTADRVPHVSVKLPGQEDKPRRTSVDARCLVVW     281
phiK        FTQYSDNPNRGIADRVAHCAFILPGTEDKPVRESVEVRALVVF     276
            ****** ***. **** * :. *** **** * **:.*.***:
```

**Figure S11**: An alignment of ZopL9 and PhiK – an uncharacterised protein encoded by the phomoidride gene cluster[28] – which have 44.16 % sequence identity. The $Fe^{2+}$ binding H-X-D/E-$X_n$-H motif and the 2-oxoglutarate binding R-X-S motif are shown in red.

## Gene Disruption Procedures

Genes were knocked out using the bipartite method.[29] This approach involves splitting the selectable marker into two overlapping fragments, each of which are fused to regions homologous to the region to be targeted. (with a ~500 bp overlap). The splitting of the selectable marker means that homologous recombination is required to reconstruct the cassette and allow selection, which in turn improves the frequency of integration via homologous recombination, and thus gene disruption.  A cassette containing the HygR gene for hygromycin resistance (accession number CAA83647) with the *gpdA* promoter and *trpC* terminator of *A. nidulans*, was used as the resistance marker in the transformations of both *S. album* and *D. curvata*. Knockout cassettes were constructed using homologous recombination in yeast using the plasmid pE-YA. These were then used as templates to produce the two bipartite fragments. The general approach is outlined in Figure S12.

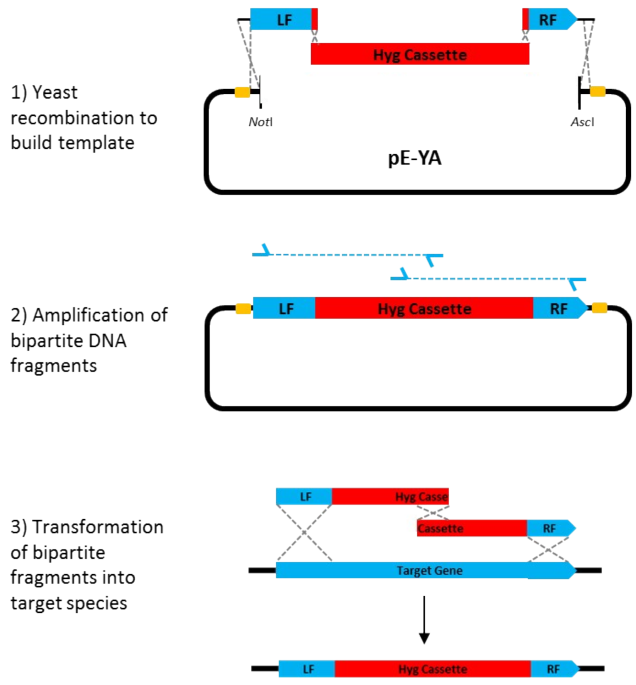


**Figure S12**: The bipartite approach to gene disruptions.

Knock-out cassettes were constructed for *zopPKS*, *scyPKS* and *scyL2*. The primer pair HygR1-F/HygR4-R was used to amplify the hygromycin cassette which formed the centre of the knock-out construct. The left and right flanking regions were amplified from either *D. curvata* or *S. album* genomic DNA using the following primer pairs (See Table S4 for primer sequences):

ZopPKS-LHF/ ZopPKS-LHR and ZopPKS-RHF/ZopPKSRHR for the left and right flanking regions of *zopPKS*.
ScyPKS-LHF/ ScyPKS-LHR and ScyPKS-RHF/ScyPKSRHR for the left and right flanking regions of *scyPKS*.
ScyL2-LHF/ ScyL2-LHR and ScyL2-RHF/ScyL2RHR for the left and right flanking regions of *ScyL2*.

To amplify the required fragments for the transformation, the forward primer for the left flanking region (ZopPKS-LHF, ScyPKS-LHF or ScyL2-LHF) was combined with the primer HygR2-R, and HygR3-F was combined with the reverse primer for the right flanking region (ZopPKS-LHR, ScyPKS-LHR or ScyL2-LHR).

## Genetic Characterisation of Knockout Strains

Agar plugs of transformants were used to inoculate 100 mL PDB (*D. curvata*) or MEB (*S. album*). Transformants were cultured at 25 °C with 200 rpm shaking for 3-5 days. The mycelia were freeze dried and then gDNA was

extracted. PCR was used to test the integration of the knock-out constructs using primers designed within the resistance cassette and outside the homologous flanking regions (Figure S13). To check for genetic purity of the transformants, primer LH-check was combined with the 'purity' primer for the specific gene; e.g. ZopPKS-LH-check / ZopPKS-purity (See Table S4 for primer sequences). Transformants that were shown to be correct by PCR were then subjected to chemical analysis.

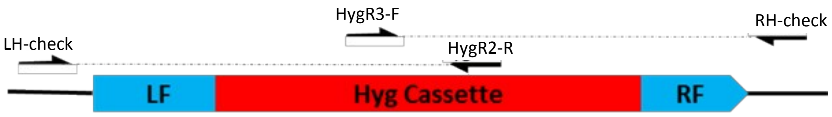


**Figure S13**: PCR used to confirm correct integration for gene disruption

## Primer Table

**Table S4**: Primers used in this study. Compound primers, with tails (in bold) were designed to allow for yeast recombination. All primers were synthesized by Sigma.

| Primer name | Sequence (5'-3') | Notes |
|---|---|---|
| HygR1-F | CATGATGGGGATCCTCTAGTG | Primers for the amplification of the split hygromycin resistance cassette. |
| HygR2-R | CTCCAACAATGTCCTGACG | |
| HygR3-F | CTGTCGAGAAGTTTCTGATCG | |
| HygR4-R | CACATCTCCACTCGACCTG | |
| ZopPKS-LHF | **TAATGCCAACTTTGTACAAAAAAGCAGGCT**<br>CCTTTAAGGAAACTAAACCG | Zopfiellin PKS knockout primers. |
| ZopPKS-LHR | **CGAAAGATCCACTAGAGGATCCCCATCATG**<br>TGATCACGGCGCTAAGAA | |
| ZopPKS-RHF | **AGCGCCCACTCCACATCTCCACTCGACCTG**<br>ACATAACTAGTCTCAAGGGG | |
| ZopPKS-RHR | **AATGCCAACTTTGTACAAGAAAGCTGGGT**<br>CTAAGTCTACCTTCTGGGTC | |
| SaPKS-LHF | **TAATGCCAACTTTGTACAAAAAAGCAGGCT**<br>CATGAGGAAGAAGTCCAT | Scytalidin PKS knockout primers. |
| SaPKS-LHR | **CGAAAGATCCACTAGAGGATCCCCATCAT**<br>**G**GAACTTTGCCAACTCATC | |
| SaPKS-RHF | **AGCGCCCACTCCACATCTCCACTCGACCTG**<br>CTCAATGTCTGTGCTTGT | |
| SaPKS-RHR | **AATGCCAACTTTGTACAAGAAAGCTGGGT**<br>CTTAGTTGCTCTCCATCTC | |
| SaL2-LHF | **TAATGCCAACTTTGTACAAAAAAGCAGGCT**<br>GTCAGCACTTAGACAAACGA | ScyL2 knockout primers. |
| SaL2_LHR | **CGAAAGATCCACTAGAGGATCCCCATCATG**<br>GTCTATGGCGTCCTATCAAC | |
| SaL2-RHF | **AGCGCCCACTCCACATCTCCACTCGACCTG**<br>CATCTCACCTGAATGGGTA | |
| SaL2-RHR | **AATGCCAACTTTGTACAAGAAAGCTGGGT**<br>GTGGACTCGAGGATTCTG | |
| ZopPKS-LH-check | GAGGAGATGTATGCTGGCA | Primers to test integration of knockout constructs and purity. |
| ZopPKS-RH-check | AGACCTCCACCATGGCTC | |
| ZopPKS-purity | GCTAAGTGCTAAGATTGCGC | |
| SaPKS-LH-check | GTAACAACGAGGAACGCTTA | |
| SaPKS-RH-check | CCGATCGACACAAATACAT | |
| SaPKS-purity | CCAGAGATACCAGTAAGAAC | |
| ScyL2-LH-check | CGATAAGGAGGGGTTGAC | |
| ScyL2-RH-check | GGGCAACGAGTTGTTTAGAT | |
| ScyL2-purity | GCTCTGAAACATATTGTGCC | |

## Transformation Protocols

### *S. cerevisiae* transformation

Yeast homologous recombination was used to build knock-out constructs and was carried out as previously described.[30] An overnight culture of *S. cerevisiae* strain YPH499 (10 mL YPAD: 1% (w/v)yeast extract, 2% (w/v) bactotryptone, 2% (w/v) glucose, 0.04% (w/v) adenine sulphate, at 28 °C shaking at 200 rpm) was added to 40 mL of YPAD and incubated for 5 hours (28 °C shaking at 200 rpm). Cells were harvested by centrifugation; 3000 x g for 5 minutes, the supernatant discarded, and cells washed with 10 mL H2O. The pellet was resuspended in 1 mL 0.1 M LiOAc and transferred to a 1.5 mL microcentrifuge tube. Cells were pelleted at 14,000 x g and resuspended in 400 µL 0.1 M LiOAc. 50 µL per transformation was transferred to a new microcentrifuge tube and pelleted as before. After discarding the supernatant, 240 µl of PEG solution (50% (w/v) polyethylene glycol 3350), 36 µl 1 M LiOAc, 50 µl SS-DNA (denatured salmon testis DNA, 2 mg/ml in TE buffer) and up to 34 µl of DNA were added to the cells in order. Approximately 0.1 µg of each DNA fragment was added, with linear DNA fragments to be joined containing at least 30 bp overlap. Cells were resuspended in the transformation mixture by vortexing, and incubated at 30 °C for 30 min and then 42 °C for 30 min. The cells were pelleted at 6000 rpm for 15sec then gently resuspended in 1 ml of sterile H2O. 200 µl aliquots were spread on SM-URA plates (0.17% (w/v) yeast nitrogen base, 0.5% (w/v) ammonium sulphate, 2% (w/v) glucose, 0.077% (w/v) complete supplement mixture minus uracil(Q-biogene), 1.5% (w/v) agar) and incubated at 28 °C for 3-4 days until colonies appeared.

### *D. curvata* transformation

A seed culture of *D. curvata* grown in PDB was homogenised and used to inoculate a 500 mL non-baffled flask containing 100 ml of PDB. After 2 days the culture was transferred into two 50 ml centrifuge tubes and centrifuged at 6500 x g for 10 min. The supernatant was discarded and then the hyphae were washed with water followed by 0.8 M NaCl. Filter sterilized protoplasting solution (20 mL of 20 mg/ mL Trichoderma lysing enzyme, 5mg/ mL driselase in 0.8 M NaCl) was added to the mycelia and gently mixed for 3 h. To release the protoplasts from the hyphae the mixture was pipetted gently and then filtered through a sterile miracloth. The protoplasts were centrifuged at 3000 x g for 5 min and then washed with solution 1 (0.8 M NaCl, 10 mM $CaCl_2$, 50 mM Tris-HCl pH 7.5). The supernatant was discarded and then the protoplasts were resuspended in solution 1 (300 µl). For each transformation 100 µl was transferred into a 10 ml tube. 10 µl of DNA (bipartite fragments for gene disruption) was added to each tube, gently mixed and incubated on ice for 10 min. 1 ml of solution 2 (0.8 M NaCl, 10mM $CaCl_2$, 50 mM Tris-HCl pH 7.5, 1M PEG 3000) was added to each tube, mixed gently, and incubated at RT for 20 min. Molten PDB (50 °C) containing 0.8 % agar and 1 M sorbitol was added, gently mixed and then poured onto PDA plates which contained 1 M sorbitol. The plates were incubated at 25 °C for 4 days before an overlay containing 0.8 % PDA and hygromycin B (50 µg/ mL) was added. Colonies were then transferred to fresh PDA plates containing hygromycin B. Transformants were sub-cultured onto hygromycin plates a further two times.

### *S. album* transformation

A seed culture of *S. album* grown in GN was homogenised and used to inoculate a 500 mL non-baffled flask containing 100 ml of GN. After 1 day shaking the culture was transferred into two 50 ml centrifuge tubes and centrifuged at 6500 x g for 10 min. The supernatant was discarded and then the hyphae were washed with water followed by 0.8 M NaCl. Filter sterilized protoplasting solution (20 mL of 20 mg/ mL Trichoderma lysing enzyme, 5mg/ mL driselase in 0.8 M NaCl) was added to the mycelia and gently mixed for 2 h. To release the protoplasts from the hyphae the mixture was pipetted gently and then filtered through a sterile miracloth. The protoplasts were centrifuged at 3000 x g for 5 min and then washed with solution 1 (0.8 M NaCl, 10 mM $CaCl_2$, 50 mM Tris-HCl pH 7.5). The supernatant was discarded and then the protoplasts were resuspended in solution 1 (300 µl). For each transformation 100 µl was transferred into a 10 ml tube. 10 µl of DNA (bipartite fragments for gene

disruption) was added to each tube, gently mixed and incubated on ice for 10 min. 1 ml of solution 2 (0.8 M NaCl, 10mM $CaCl_2$, 50 mM Tris-HCl pH 7.5, 1M PEG 3000) was added to each tube and incubated at RT for 20 min. Molten MEA containing 0.8 % agar and 1 M sorbitol was added, gently mixed and then poured onto MEA plates which contained 1 M sorbitol. The plates were incubated at 25 °C for 2 days before an overlay containing 0.8 % MEA and hygromycin B (50 µg/ mL) was added. Colonies were then transferred to fresh MEA plates containing hygromycin B. Transformants were sub-cultured onto hygromycin plates a further two times.

## Metabolite Extraction of *D. curvata*

For liquid cultures, the cultures were extracted after 8 days. To extract the cultures were acidified to pH 3 using 2 M HCl and the mycelia and broth were blended. The mycelia were removed by filtering and an equal volume of EtOAc was added to the broth. The aqueous layer was washed a further two times with EtOAc. The organic extracts were combined, dried with $MgSO_4$ and concentrated under vacuum. For *D. curvata* grown on rice, after 21 – 28 days rice was soaked in EtOAc (500 mL), blended and acidified using HCl. Water was added and then the organic and aqueous layers separated. The aqueous layer was washed a further two times with EtOAc. The combined organic layers were dried with $MgSO_4$ and then concentrated under reduced pressure. The crude organic extract was dissolved in acetonitrile and defatted with hexane.

## Metabolite Extraction of *S. album*

For liquid cultures, the culture was extracted after 12 days by acidifying and then blending. The broth was extracted using an equal volume of EtOAc. The combined organic extracts were dried with $MgSO_4$ and concentrated under reduced pressure. For *S. album* grown on rice or oats, after 21 – 28 days, the rice or oats were soaked in EtOAc (500 mL), blended and acidified to pH 3 using HCl. Water was added and the organic and aqueous layers separated. The aqueous layer was washed a further two times with EtOAc. The combined organic layers were dried with $MgSO_4$ and then concentrated under reduced pressure. The crude organic extract was dissolved in acetonitrile and defatted by washing with hexane.

# HPLC Data

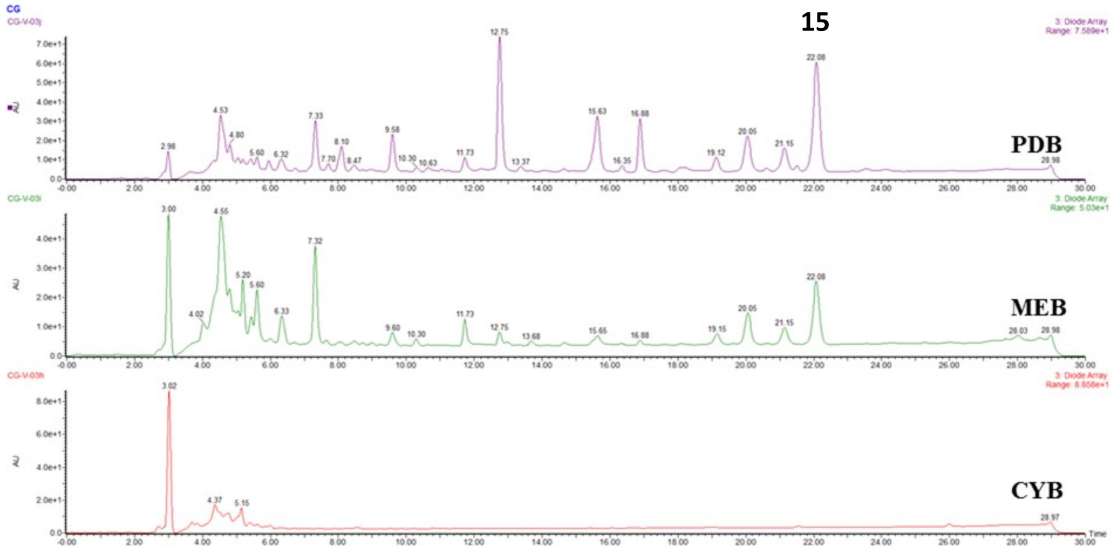


**Figure S14**. HPLC analysis of crude extracts from various *D. curvata* liquid cultures, analysed with an HLPC-UV/MS/ELSD system (gradient 50-90% $CH_3CN:H_2O$). Zopfiellin **15** can be seen eluting at 22.1 minutes.

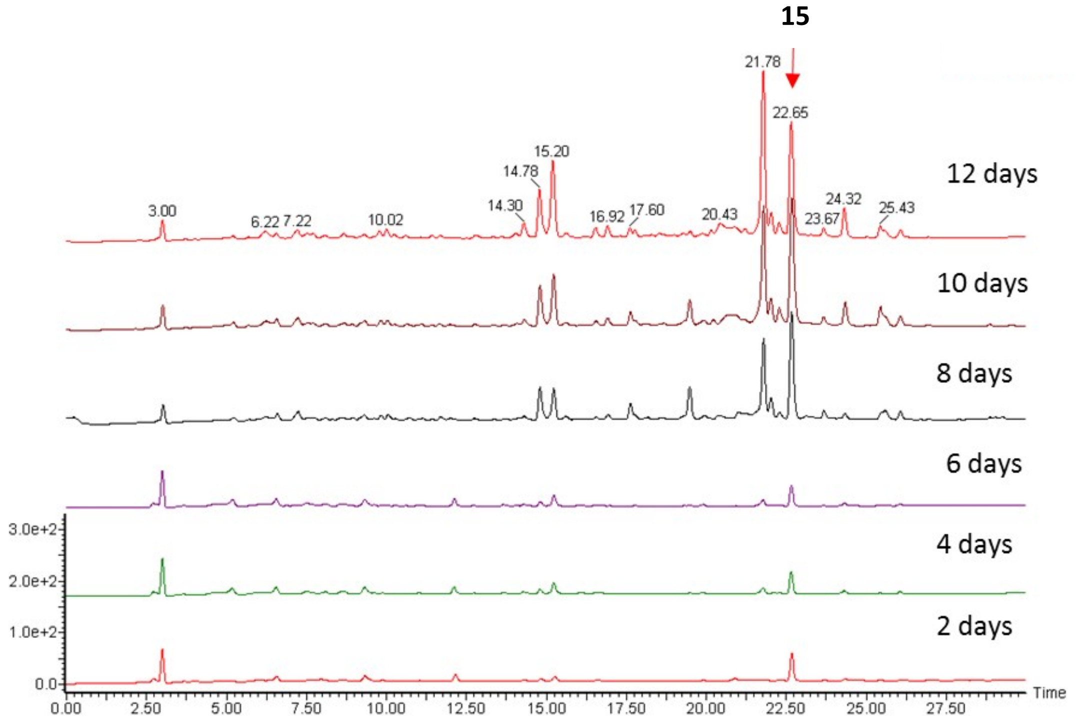


**Figure S15**. Timecourse for the production of zopfiellin **15** (22.7 min) in PDB, analysed by LCMS. Low levels of zopfiellin may be present at early time points due to the seed culture used, but a clear increase in zopfiellin in the cultures was seen by 8 days.

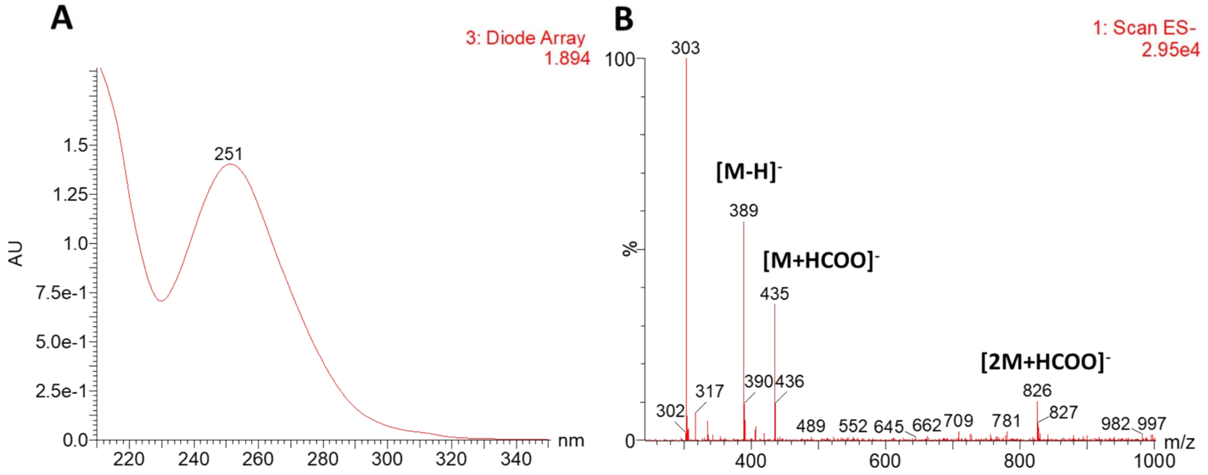


**Figure S16**. LCMS analysis of zopfiellin **15** eluting at 22.1 min (Figure S15): A, UV spectrum; B, ES$^-$ spectrum.

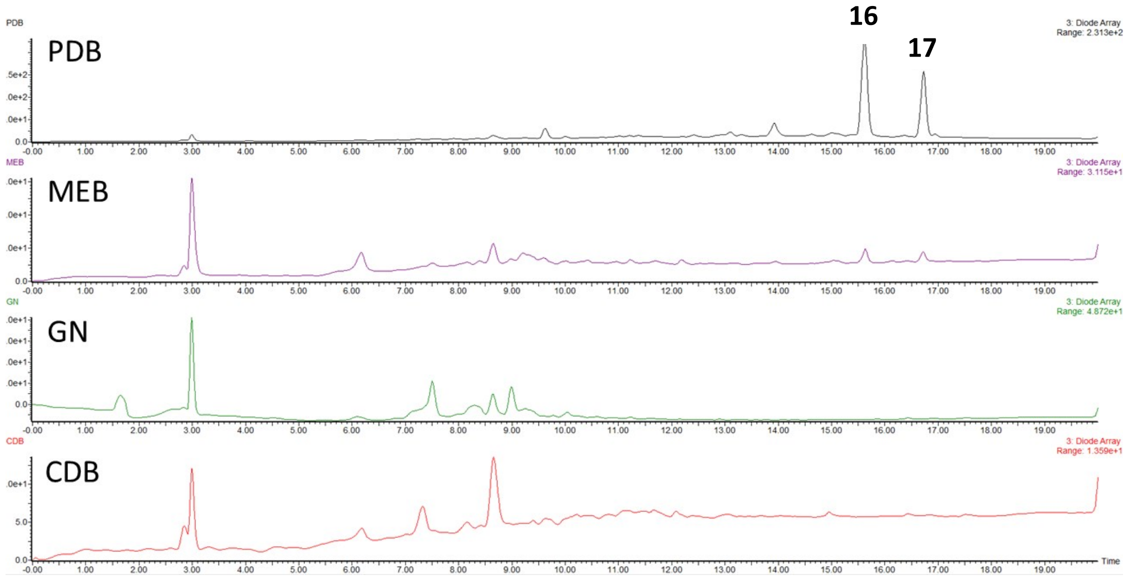


**Figure S17**: HPLC analysis of crude extracts from a range of *S. album* UAMH 3620 liquid cultures, analysed with an HLPC-UV/MS/ELSD system (gradient 5-95% $CH_3CN$:$H_2O$). Scytalidin **16** and deoxyscytalidin **17** can be seen eluting at 15.6 and 16.7 minutes in PDB and MEB crude extracts.

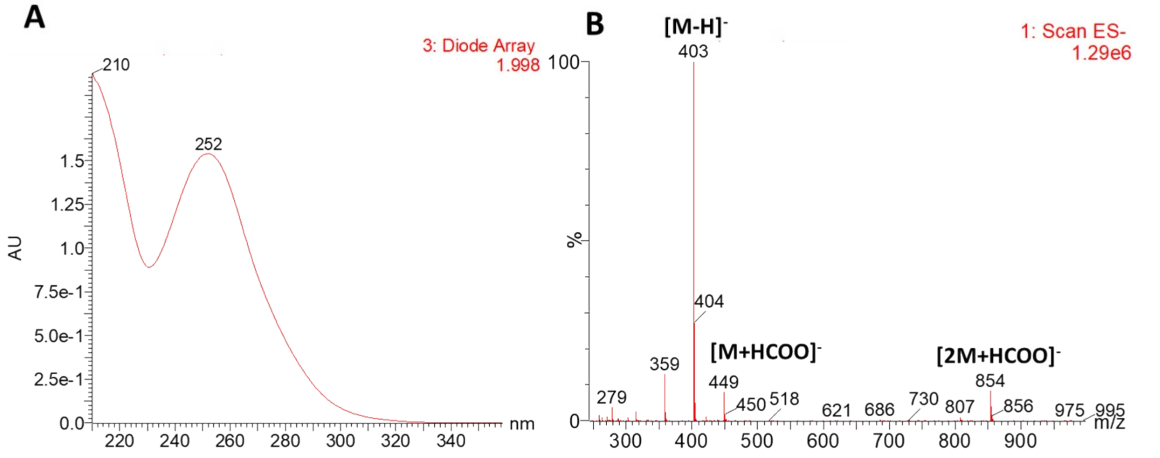


**Figure S18**: LCMS analysis of scytalidin **16**: A, UV spectrum; B, ES$^-$ spectrum.

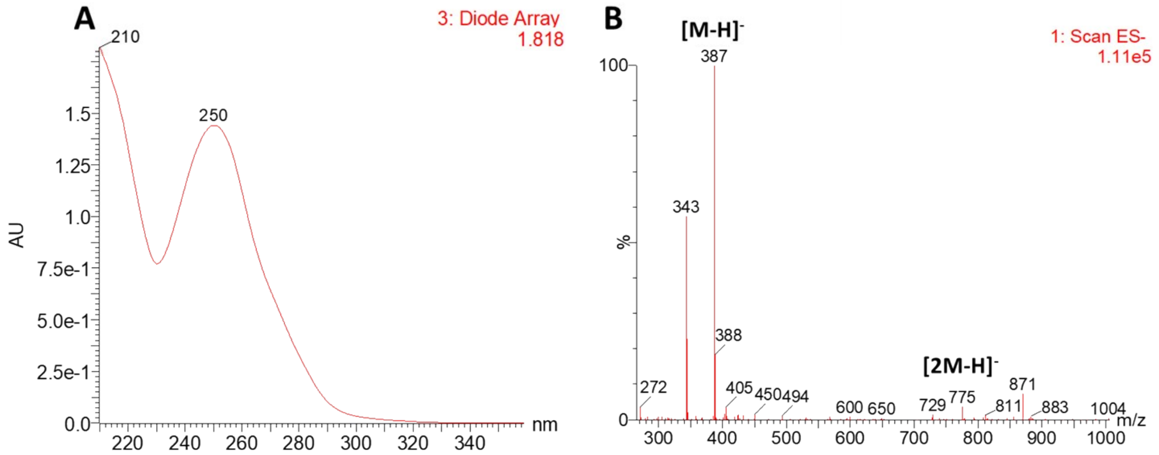


**Figure S19**: LCMS analysis of deoxyscytalidin **17**: A, UV spectrum; B, ES$^-$ spectrum.

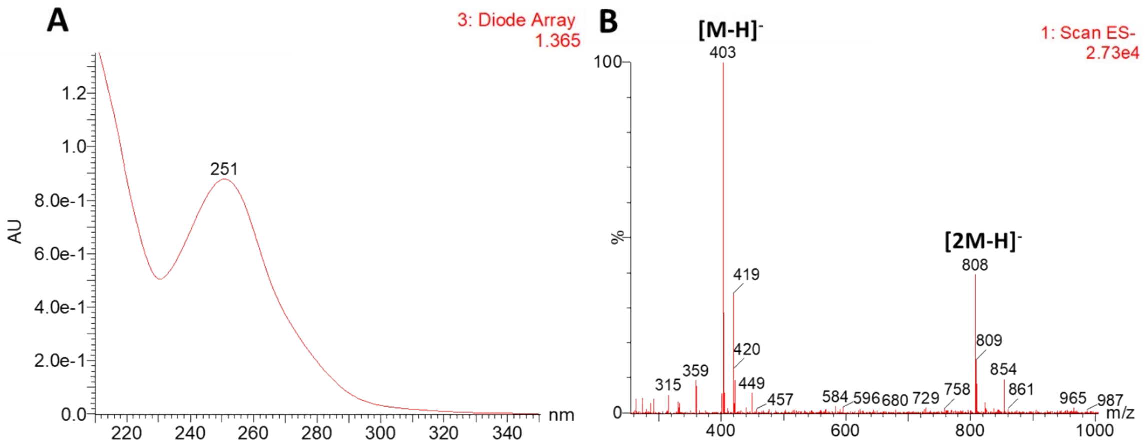


**Figure S20**: LCMS analysis of 5-hydroxy-deoxyscytalidin **19**: A, UV spectrum; B, ES$^-$ spectrum.

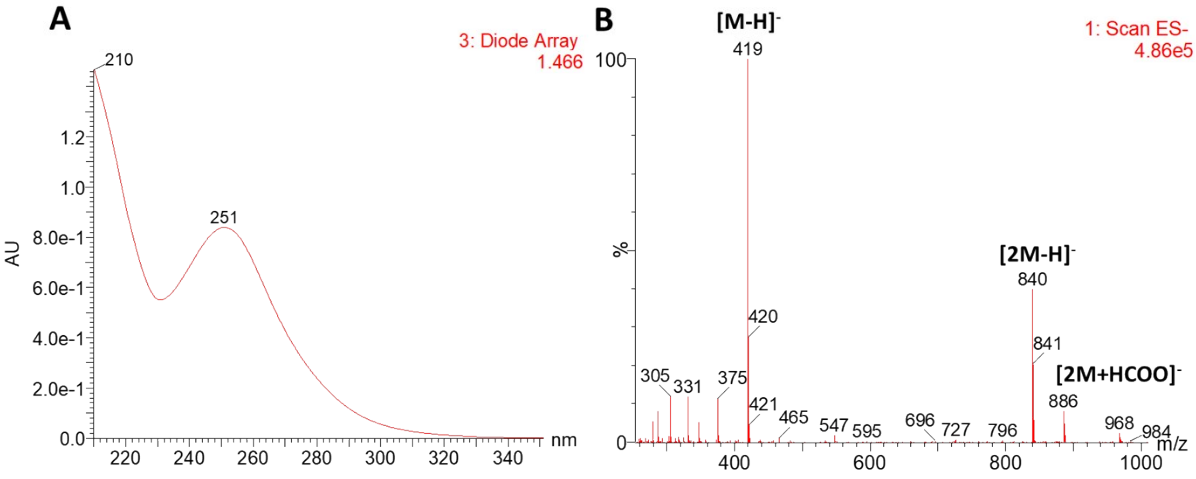


**Figure S21**: LCMS analysis of castaneolide **6**: A, UV spectrum; B, ES$^-$ spectrum.

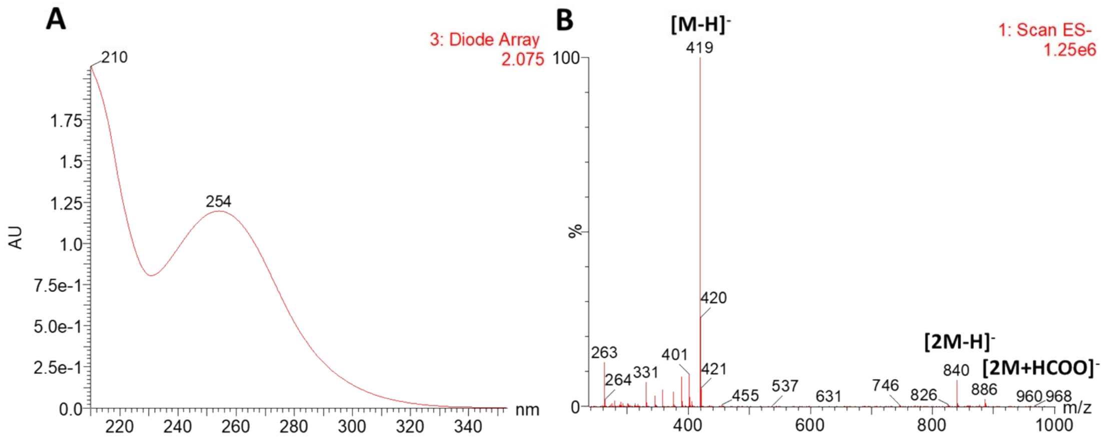


**Figure S22**: LCMS analysis of compound **20**: A, UV spectrum; B, ES$^-$ spectrum.

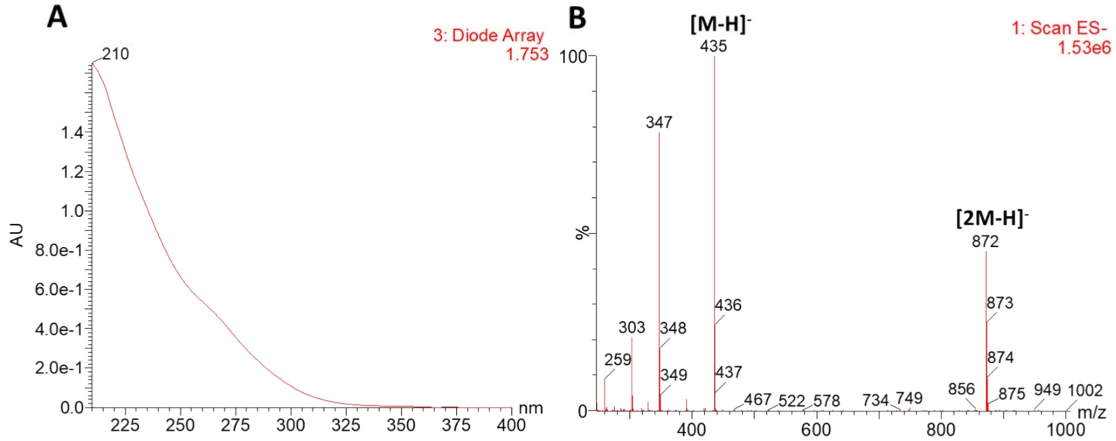


**Figure S23**: LCMS analysis of compound **21**: A, UV spectrum; B, ES$^-$ spectrum.

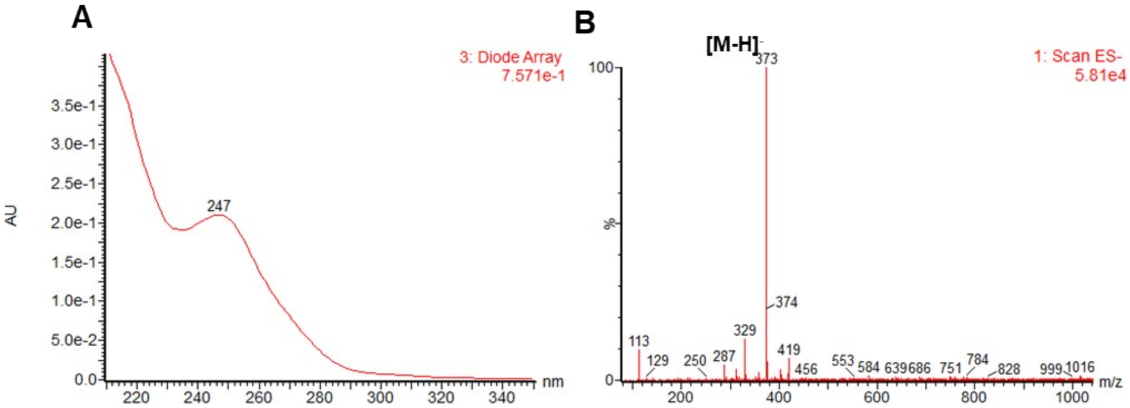


**Figure S24**: LCMS analysis of compound **24**: A, UV spectrum; B, ES$^-$ spectrum.

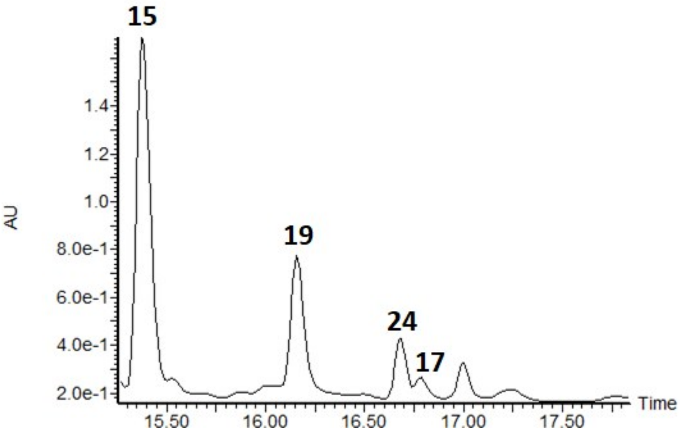


**Figure S25**: HPLC analysis of a crude extract from a wild-type *D. curvata* PDB liquid culture identified deoxyscytalidin **17**, 5-hydroxy-deoxyscytalidin **19** and deoxyzopfiellin **24** as well as zopfiellin **15**. Retention times, UV spectra and ESIMS data was all identical to standards.

## Isolated metabolites

Castaneiolide **6** - $\lambda_{max}$ (UV) 251 nm; ESIMS (LCMS) m/z 419.2 $[M-H]^-$; $[\alpha]_D^{25}$ -63.6 (*c* 0.11 EtOH) (lit. $[\alpha]_D$ -36.5 (*c* 0.11 EtOH)[31]); for $^1H$ and $^{13}C$ NMR data see: Table S5.

Zopfiellin **15 -** $\lambda_{max}$ (UV) 251 nm; HRESIMS m/z 389.1591 $[M-H]^-$ ($C_{21}H_{25}O_7$ requires 389.1606); $[\alpha]_D^{25}$ -84.3 (*c* 0.43, MeOH) (lit. $[\alpha]_D^{23}$ -76.8 (*c* 0.42, MeOH)[32]); for $^1H$ and $^{13}C$ NMR data see: Table S6.

Scytalidin **16** - $\lambda_{max}$ (UV) 252 nm; HRESIMS m/z 427.1732 $[M+Na]^+$ ($C_{22}H_{22}NaO_7$ requires 427.1727); $[\alpha]_D^{22}$ -57.8 (*c* 0.47, EtOAc) (lit. $[\alpha]_D^{21}$ -66.6 (*c* 0.4745, EtOAc)[33]); for $^1H$ and $^{13}C$ NMR data see: Table S7

Deoxyscytalidin **17** - $\lambda_{max}$ (UV) 250 nm; HRESIMS m/z 411.1779 $[M+Na]^+$ ($C_{22}H_{28}NaO_6$ requires 411.1778); $[\alpha]_D^{22}$ −52.5 (*c* 0.13, $CHCl_3$) (lit. $[\alpha]_D$ -82.2 (*c* 0.135, $CHCl_3$)[34]); for $^1H$ and $^{13}C$ NMR data see: Table S8.

5-hydroxy-deoxyscytalidin **19 -** $\lambda_{max}$ (UV) 251 nm; HRESIMS m/z 403.1762 $[M-H]^-$ ($C_{22}H_{27}O_7$ requires 403.1762); $[\alpha]_D$ – 66.4° (*c* 0.2 $CHCl_3$); $^1H$ and $^{13}C$ NMR data see: Table S9.

**20** - $\lambda_{max}$ (UV) 254 nm; HRESIMS m/z 419.1691 $[M-H]^-$ ($C_{22}H_{27}O_8$ requires 419.1711); for $^1H$ and $^{13}C$ NMR data see: Table S10.

**21** - $\lambda_{max}$ (UV) 265 nm; HRESIMS m/z 435.1646 $[M-H]^-$ ($C_{22}H_{27}O_9$ requires 435.1655); for $^1H$ and $^{13}C$ NMR data see: Table S10.

Deoxyzopfiellin **24** - $\lambda_{max}$ 247 nm; ESIMS (LCMS) m/z 373 $[M-H]^-$; for $^1H$ and $^{13}C$ NMR data see: Table S64.

## NMR data for castaneiolide 6

**Table S5**: NMR assignment for castaneiolide **6** ([a]500 MHz, [b]125 MHz in acetone-$d_6$)

| | **Castaneiolide 6** | | |
|---|---|---|---|
| **Position** | $^b\delta_C$ | $^a\delta_H$, mult. (*J* in Hz) | HMBC |
| **1** | 35.7 | 3.36 m | 2, 8, 9, 9′, 1′′ |
| **2** | 27.7 | 3.71, t (13.5)<br>3.02, dd (13.5, 5.5) | 1, 3, 4, 3′ |
| **3** | 144.9 | - | - |
| **4** | 147.0 | - | - |
| **5** | 70.1 | 4.72, s<br>5.40, s (OH) | 3, 4, 6. 7, 4′, 1′′′ |
| **6** | 78.5 | - | - |
| **7** | 29.0 | 2.70, d (14.6)<br>3.52, d (14.6) | 5, 6, 8, 9, 8′, 1′′′ |
| **8** | 142.8 | - | - |
| **9** | 145.4 | - | - |
| **3′** | 166.3 | - | - |
| **4′** | 166.0 | - | - |
| **8′** | 168.0 | - | - |
| **9′** | 167.4 | - | - |
| **1′′** | 36.5 | 1.78, m | 1, 2, 9, 3′′ |
| **2′′** | 27.3 | 1.30 – 1.56, m | |
| **3′′** | 32.4 | 1.30 – 1.56, m | |
| **4′′** | 23.2 | 1.30 – 1.56, m | |
| **5′′** | 14.4 | 0.91, t (7.0) | 4′′, 3′′ |
| **1′′′** | 42.6 | 1.90, t (8.2) | 5, 6, 7, 2′′′, 3′′′ |
| **2′′′** | 25.7 | 1.64, m | 1′′′, 3′′′, 4′′′ |
| **3′′′** | 23.9 | 1.41, m | 1′′′, 2′′′, 4′′′ |
| **4′′′** | 14.5 | 0.97 t (7.3) | 2′′′, 3′′′ |

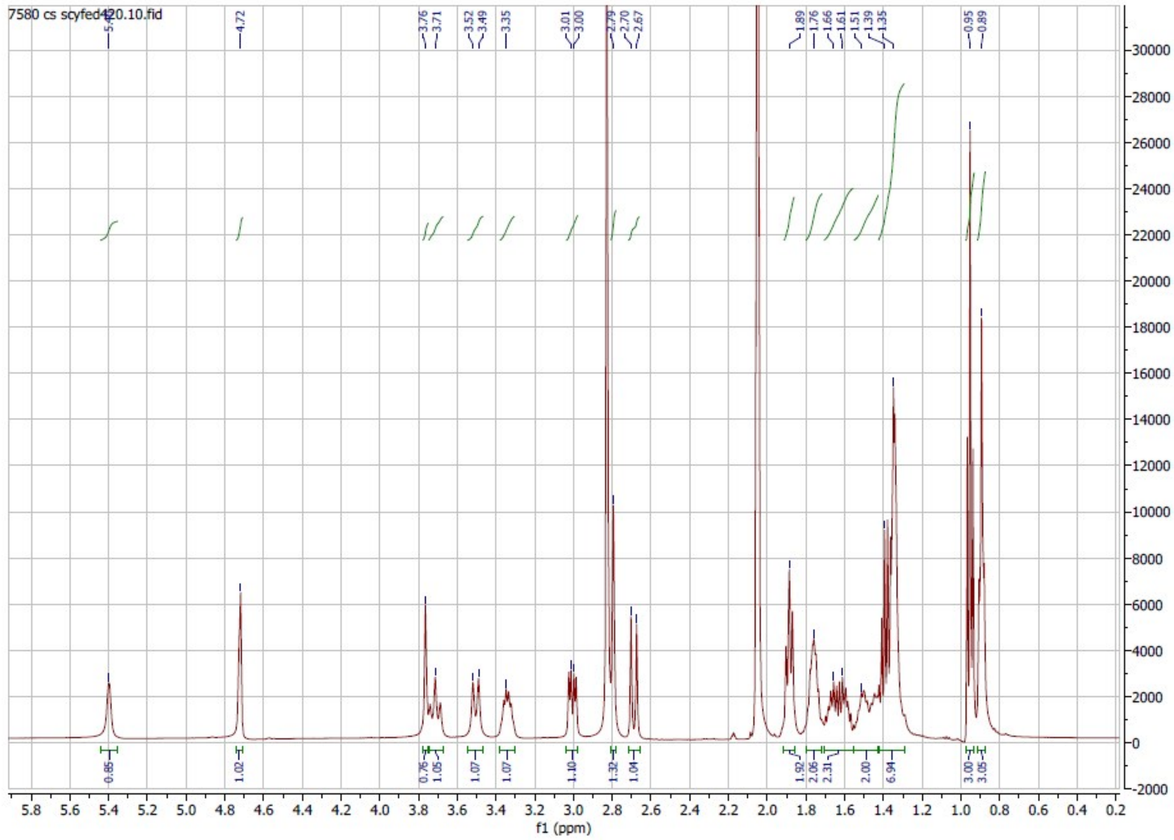

**Figure S26**: $^1$H NMR spectrum (500 MHz, Acetone-$d_6$) of **6**.

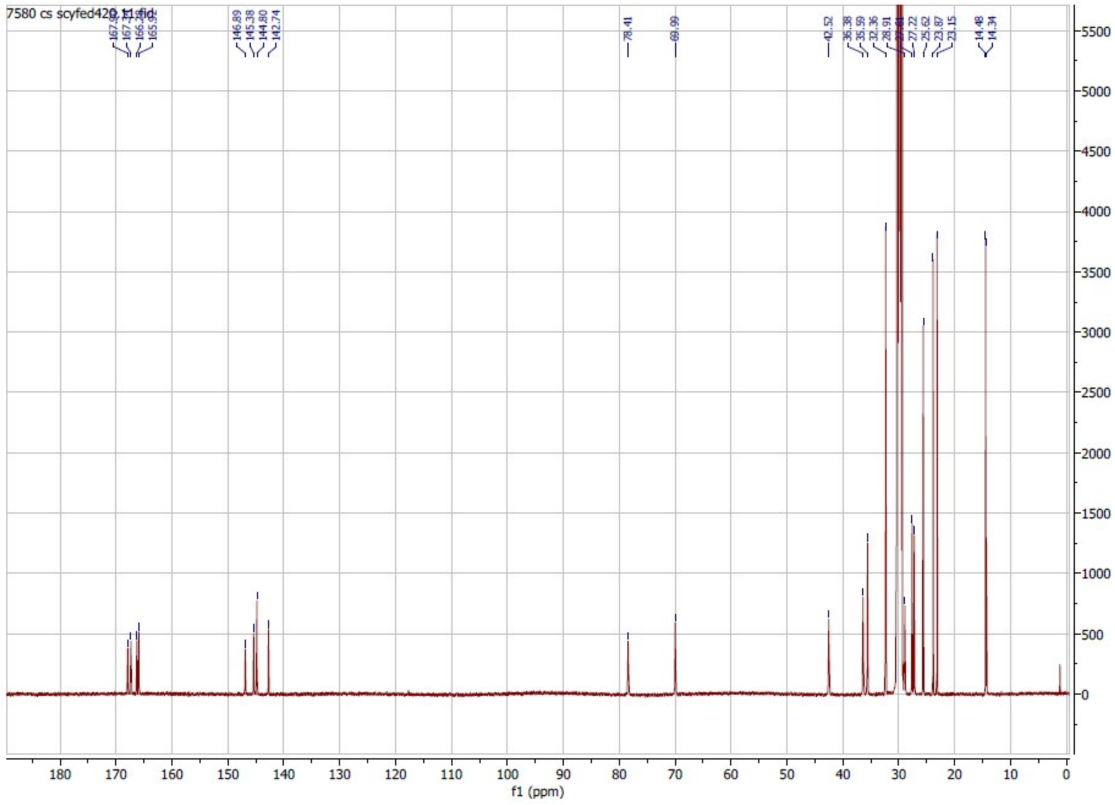

**Figure S27**: $^{13}$C NMR spectrum (125 MHz, Acetone-$d_6$) of **6**.

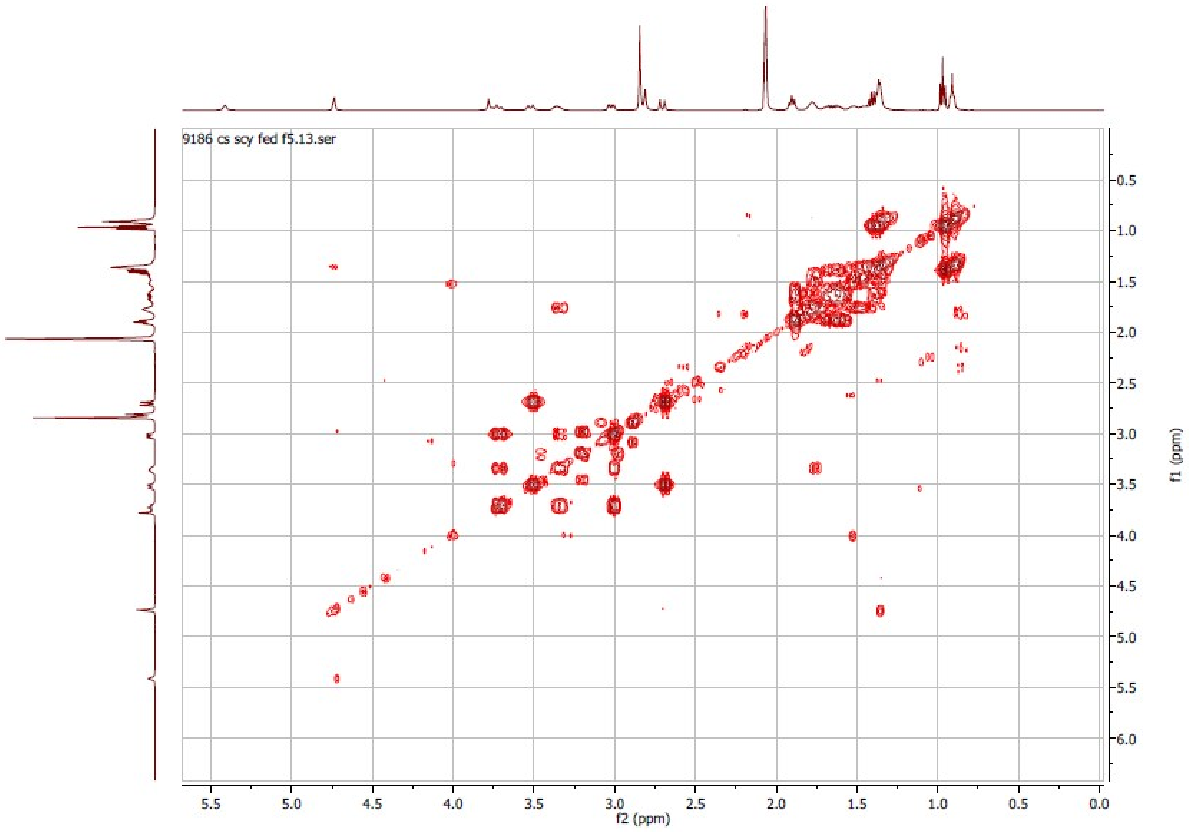


**Figure S28**: COSY spectrum of 6 (acetone-$d_6$)

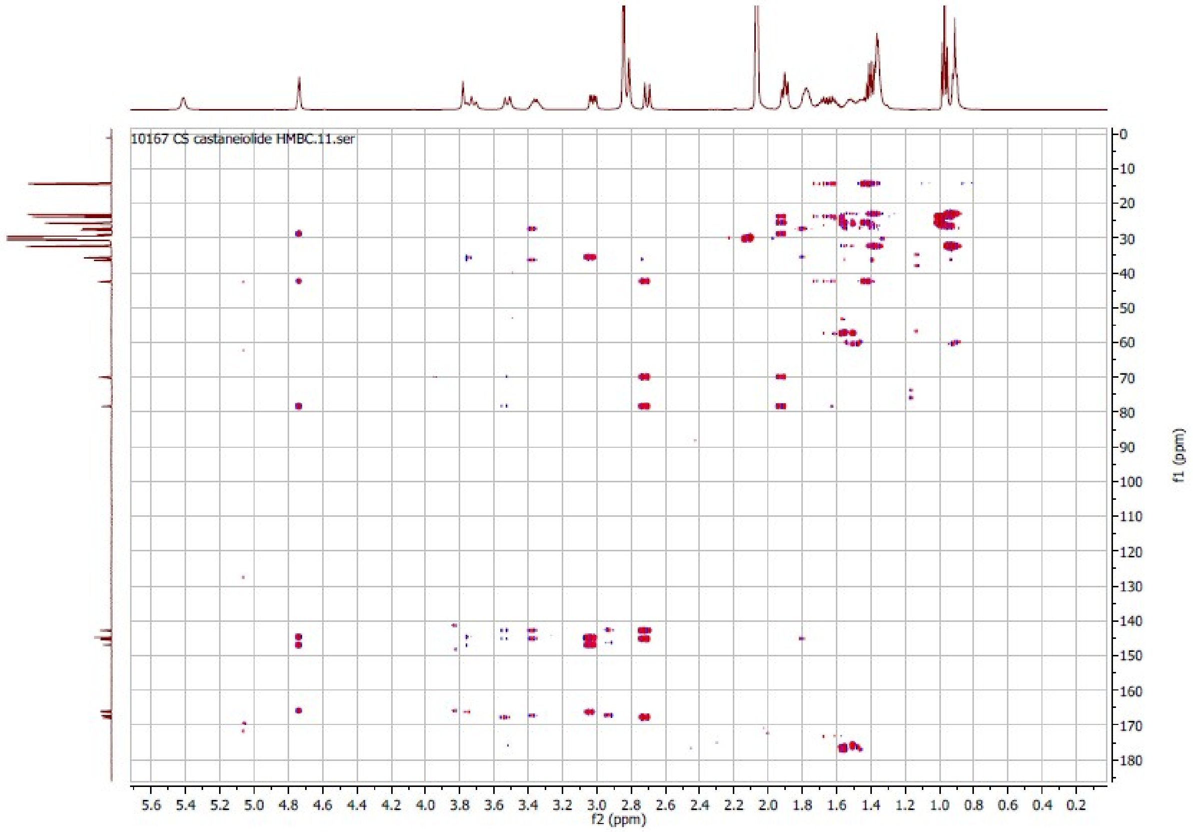


**Figure S29**: HMBC spectrum of **6** (acetone-$d_6$)

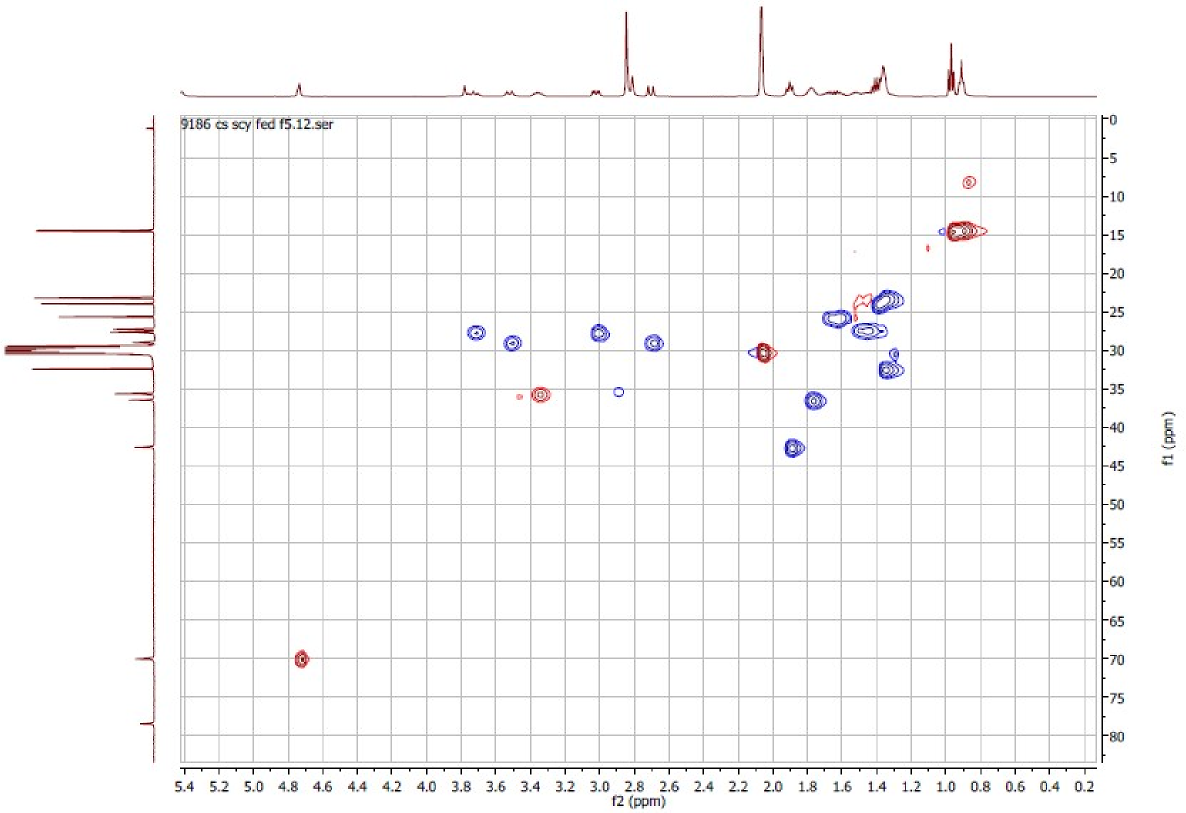


**Figure S30**: HSQC spectrum of **6** (acetone-$d_6$)

## NMR data for zopfiellin **15**

**Table S6**: NMR assignments for zopfiellin **15** ([a]500 MHz, [b]125 MHz in $CDCl_3$)

| | **Zopfiellin 15** | | |
|---|---|---|---|
| **Position** | $^{b}\delta_C$ | $^{a}\delta_H$, mult. (*J* in Hz) | HMBC |
| **1** | 41.8 | 3.02, m | 8′ |
| **2** | 18.6 | 3.29, app. t (13.0)<br>2.91, m | 1, 3, 4, 3′, 4′, 1′′ |
| **3** | 141.1 | - | |
| **4** | 147.4 | - | |
| **5** | 36.7 | 2.97, m | |
| **6** | 25.1 | 3.13, app. t (14.0)<br>2.99, m | 5, 7, 8, 7′, 2′′′ |
| **7** | 143.5 | - | |
| **8** | 145.2 | - | |
| **3′** | 165.5 | - | |
| **4′** | 165.6 | - | |
| **7′** | 165.1 | - | |
| **8′** | 165.3 | - | |
| **1′′** | 71.8 | 3.99, app. t (7.0) | |
| **2′′** | 35.0 | 1.68, m | |
| **3′′** | 28.3 | 1.41, m | |
| **4′′** | 22.6 | 1.41, m | 2′′, 3′′, 5′′ |
| **5′′** | 14.1 | 0.96, dd (7.0) | |
| **1′′′** | 28.8 | 1.41, m | |
| **2′′′** | 34.3 | 1.66, m | |
| **3′′′** | 22.5 | 1.39, m | |
| **4′′′** | 14.0 | 0.93, t (7.0) | 3′′′, 4′′′ |

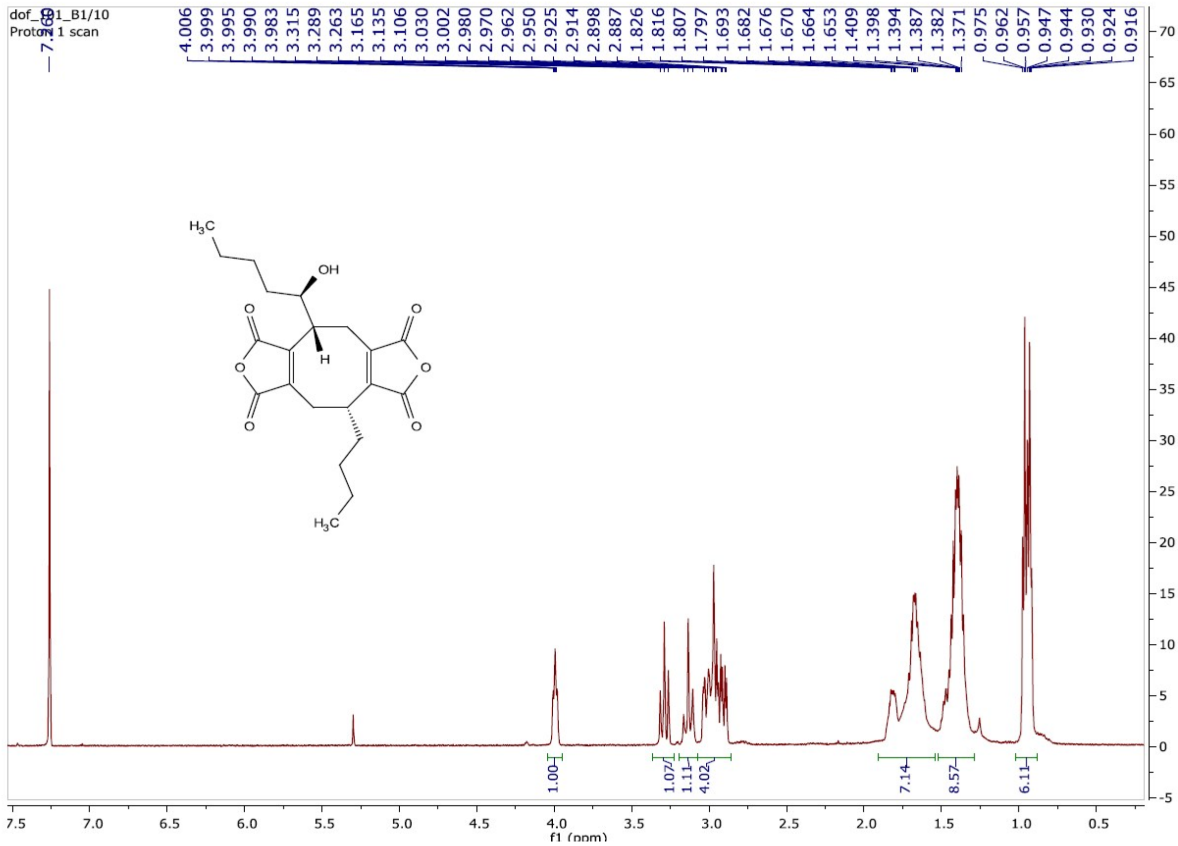


**Figure S31**: $^{1}$H NMR spectrum (500 MHz, $CDCl_3$) of **15**.

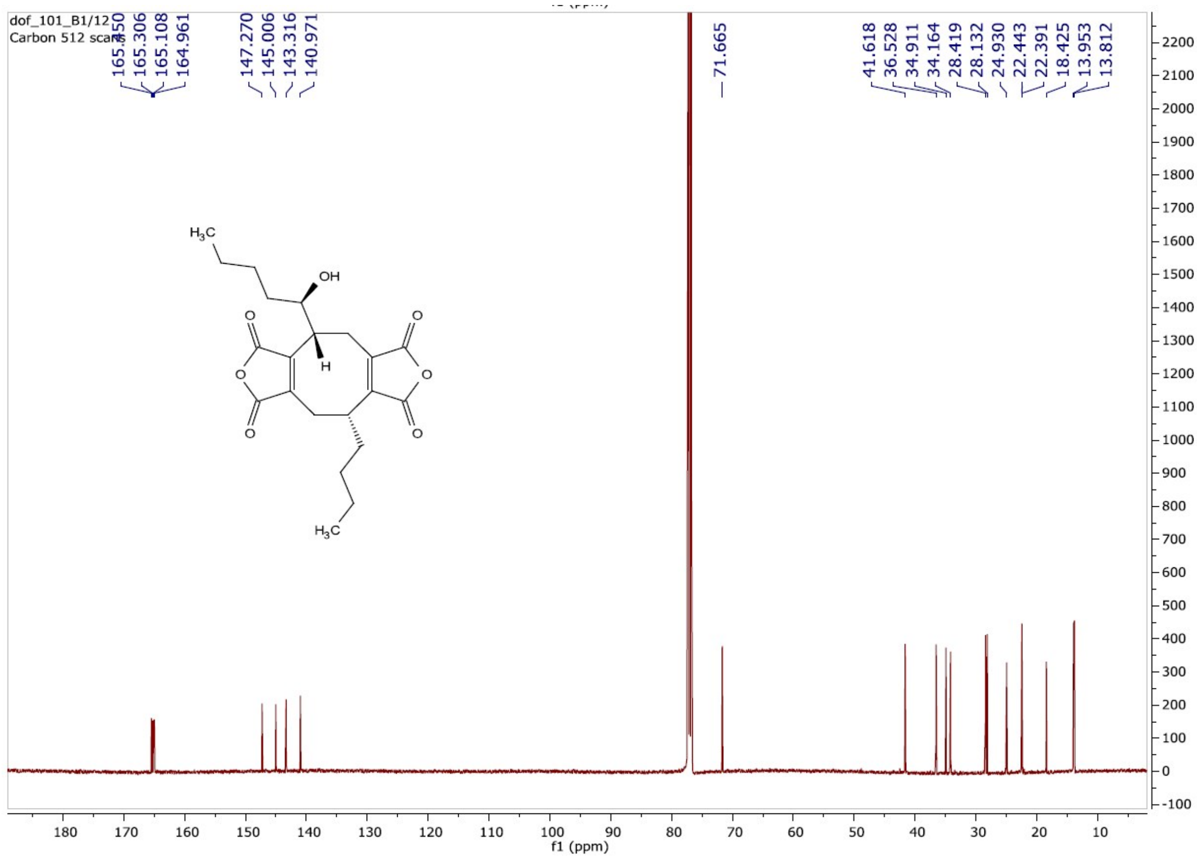


**Figure S32**: $^{13}$C NMR spectrum (125 MHz, $CDCl_3$) of **15**.

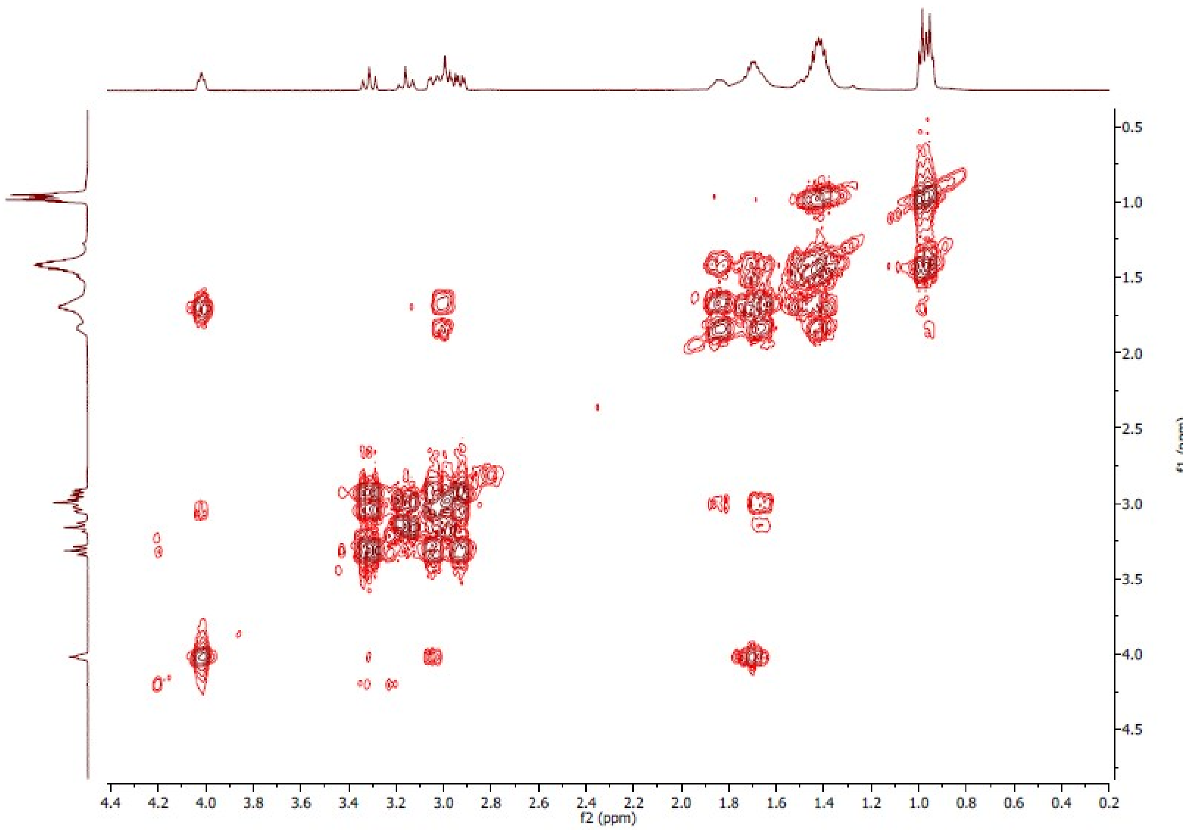


**Figure S33**: COSY spectrum of **15**.

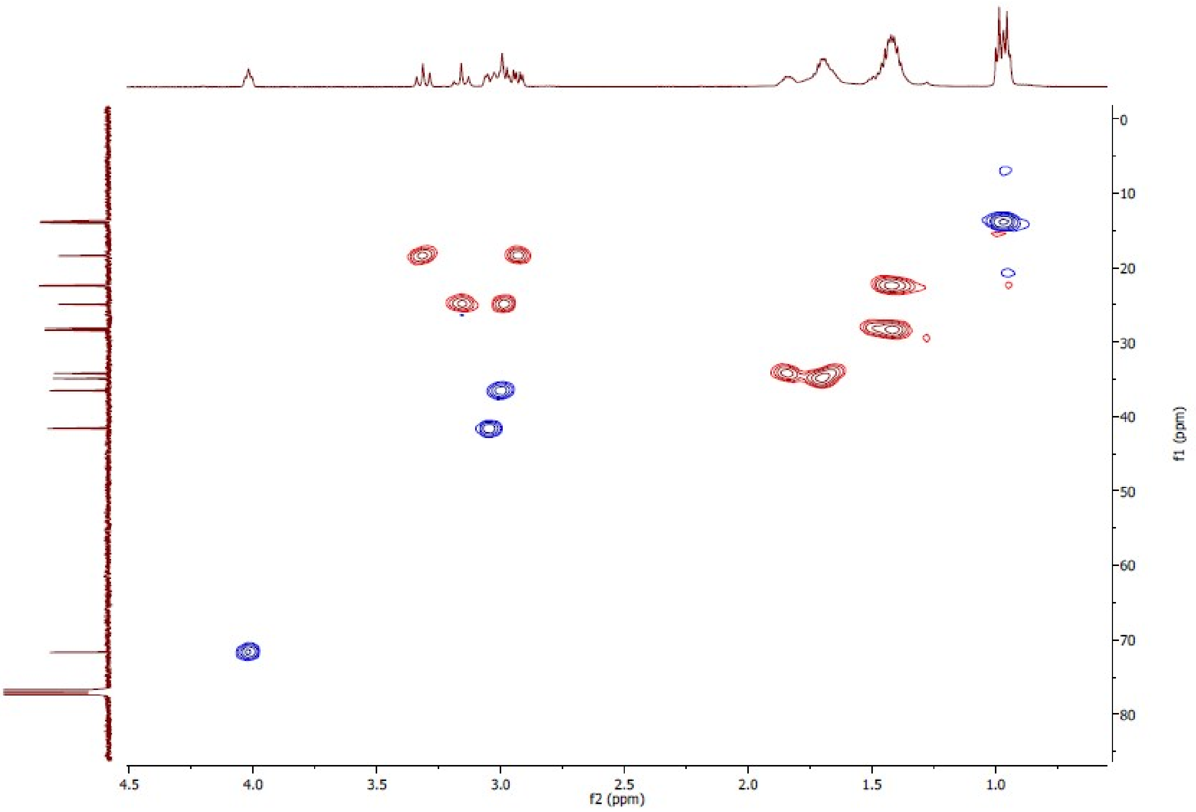


**Figure S34**: HSQC spectrum of **15**.

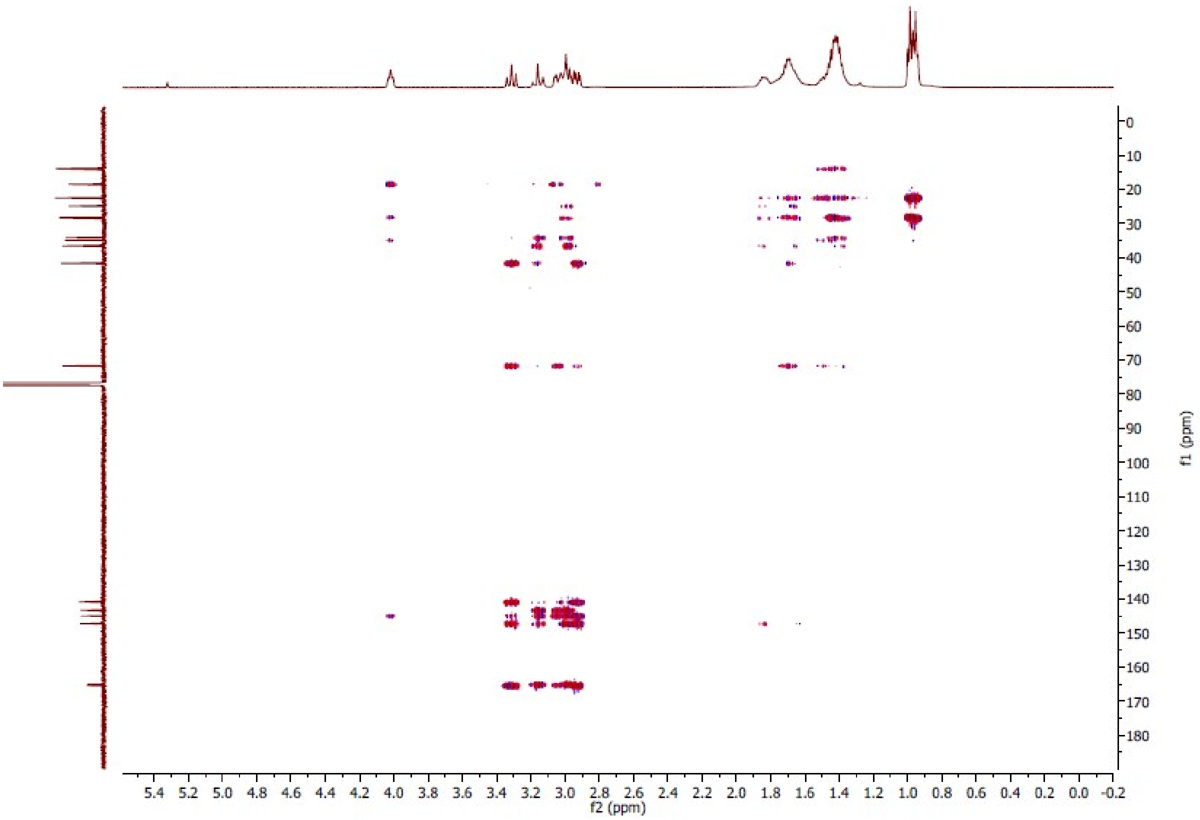

**Figure S35**: HMBC spectrum of **15**.

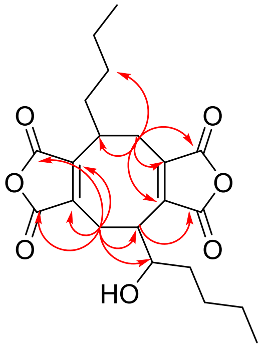


**Figure S36**: Key HMBC correlations for zopfiellin **15**

## NMR data for scytalidin **16**

**Table S7**: NMR assignments for scytalidin **16** ([a]500 MHz, [b]125 MHz in $CD_2Cl_2$)

| | **Scytalidin 16** | |
|---|---|---|
| **Position** | [b]$\delta_C$ | [a]$\delta_H$, mult. (*J* in Hz) |
| **1** | 35.4 | 3.41, m |
| **2** | 28.6 | 2.71 – 3.04, m |
| **3[a]** | 143.5 | - |
| **4[a]** | 141.9 | - |
| **5** | 35.7 | 2.71 – 3.04, m |
| **6** | 76.6 | - |
| **7** | 35.7 | 2.57, d (13.0)<br>2.71- 3.04, m |
| **8[a]** | 143.2 | - |
| **9[a]** | 145.5 | - |
| **3´[b]** | 165.4 | - |
| **4´[b]** | 168.0 | - |
| **8´[b]** | 166.0 | - |
| **9´[b]** | 165.9 | - |
| **1´´** | 35.7 | 1.63 – 1.72, m |
| **2´´** | 27.3 | 1.27 – 1.36, m<br>1.37 – 1.46, m |
| **3´´** | 32.00 | 1.27 – 1.36, m |
| **4´´** | 23.0 | 1.27 – 1.36, m |
| **5´´** | 14.3 | 0.89, t (7.0) |
| **1´´´** | 46.2 | 1.47 – 1.58, m<br>1.74 – 1.85, m |
| **2´´´** | 26.2 | 1.47 – 1.58, m<br>1.74 – 1.85, m |
| **3´´´** | 23.5 | 1.37 – 1.46, m |
| **4´´´** | 14.3 | 0.98, t (7.5) |
| **[a)] signals can be interchanged [b)] signals can be interchanged** | | |

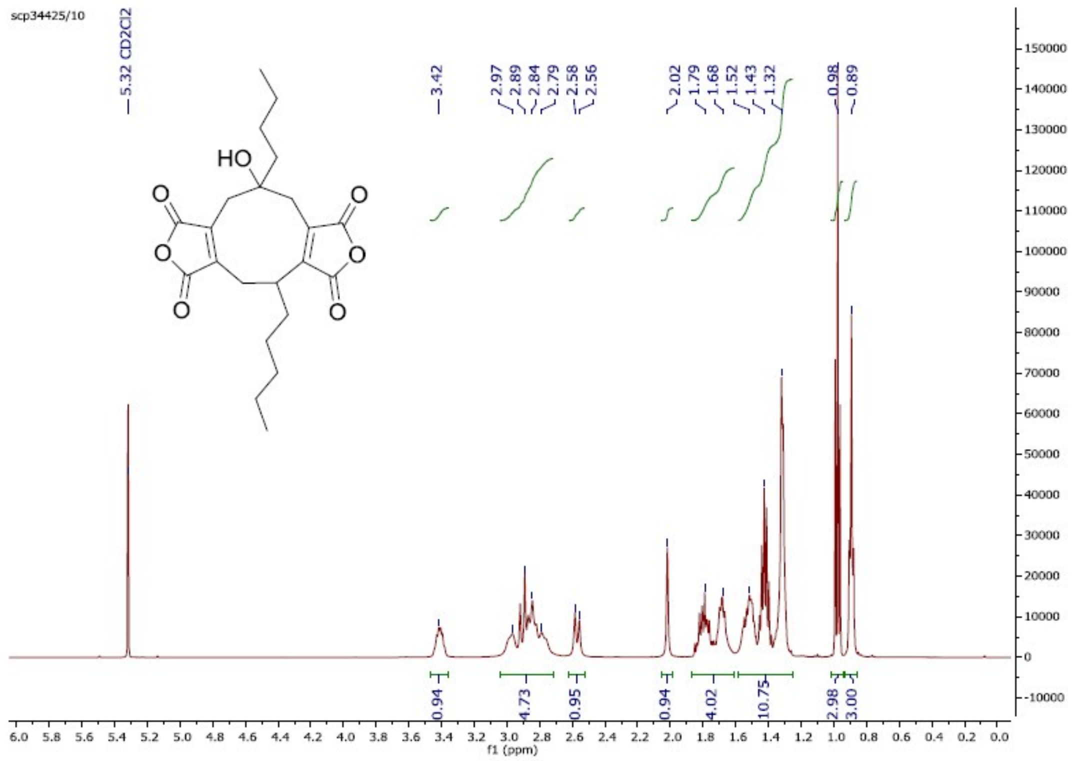


**Figure S37**: $^{1}H$ NMR spectrum (500 MHz, $CD_2Cl_2$) of **16**.

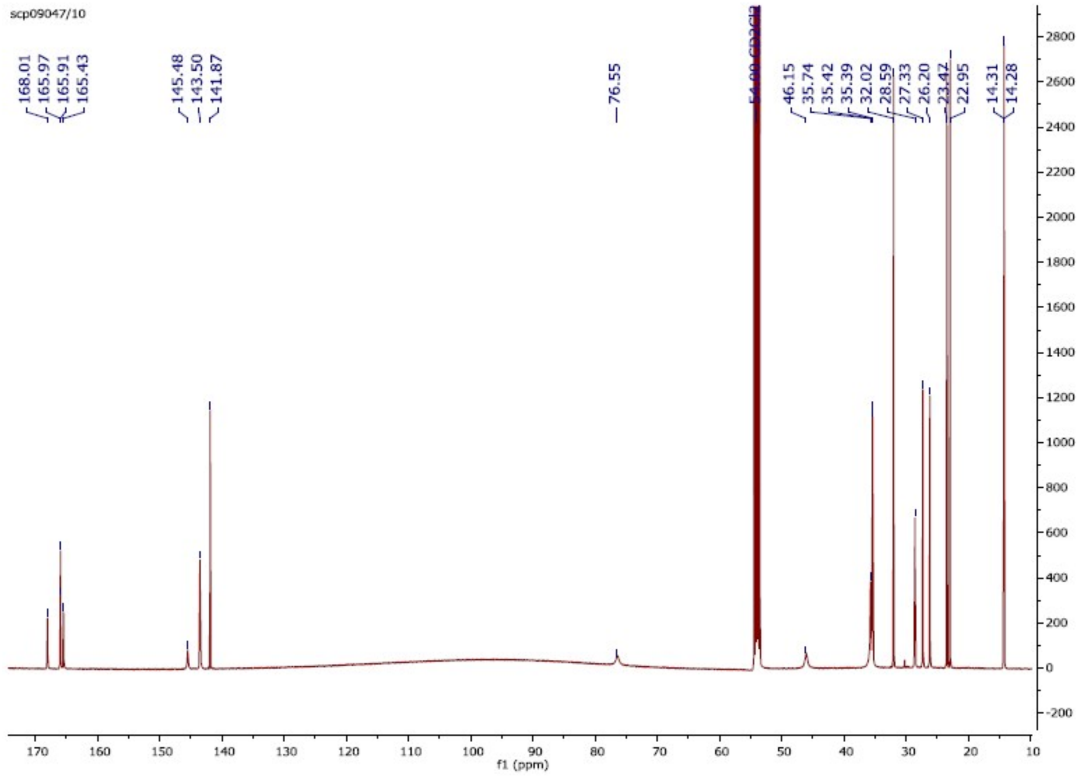


**Figure S38**: $^{13}C$ NMR spectrum (125 MHz, $CD_2Cl_2$) of **16**.

## NMR data for deoxyscytalidin **17**

**Table S8**: NMR assignments for deoxyscytalidin **17** ([a]500 MHz, [b]125 MHz in $CDCl_3$)

| | **Deoxyscytalidin 17** | |
|---|---|---|
| **Position** | $^b\delta_C$ | $^a\delta_H$, mult. ($J$ in Hz) |
| **1** | 35.1 | 3.29 – 3.42, m |
| **2** | 28.2 | 2.97-2.79, m |
| **3** | 143.3 | - |
| **4** | 144.3 | - |
| **5** | 30.6 | 2.22- 2.39, m<br>2.55- 2.75, m |
| **6** | 38.8 | 1.90- 2.05, m |
| **7** | 29.8 | 2.22- 2.39, m<br>2.55- 2.75, m |
| **8** | 143.6 | - |
| **9** | 144.8 | - |
| **3´** | 164.9 | - |
| **4´** | 165.4 | - |
| **8´** | 165.4 | - |
| **9´** | 165.6 | - |
| **1´´** | 34.1 | 1.61- 1.76, m |
| **2´´** | 27.2 | 1.35- 1.44, m<br>1.24- 1.33, m |
| **3´´** | 31.6 | 1.24 – 1.33, m |
| **4´´** | 22.5 | 1.24 – 1.33, m |
| **5´´** | 14.1 | 0.88, m |
| **1´´´** | 37.0 | 1.46- 1.59, m |
| **2´´´** | 29.2 | 1.46- 1.59, m |
| **3´´´** | 22.6 | 1.35 – 1.44, m |
| **4´´´** | 14.1 | 0.96, t (8.0) |

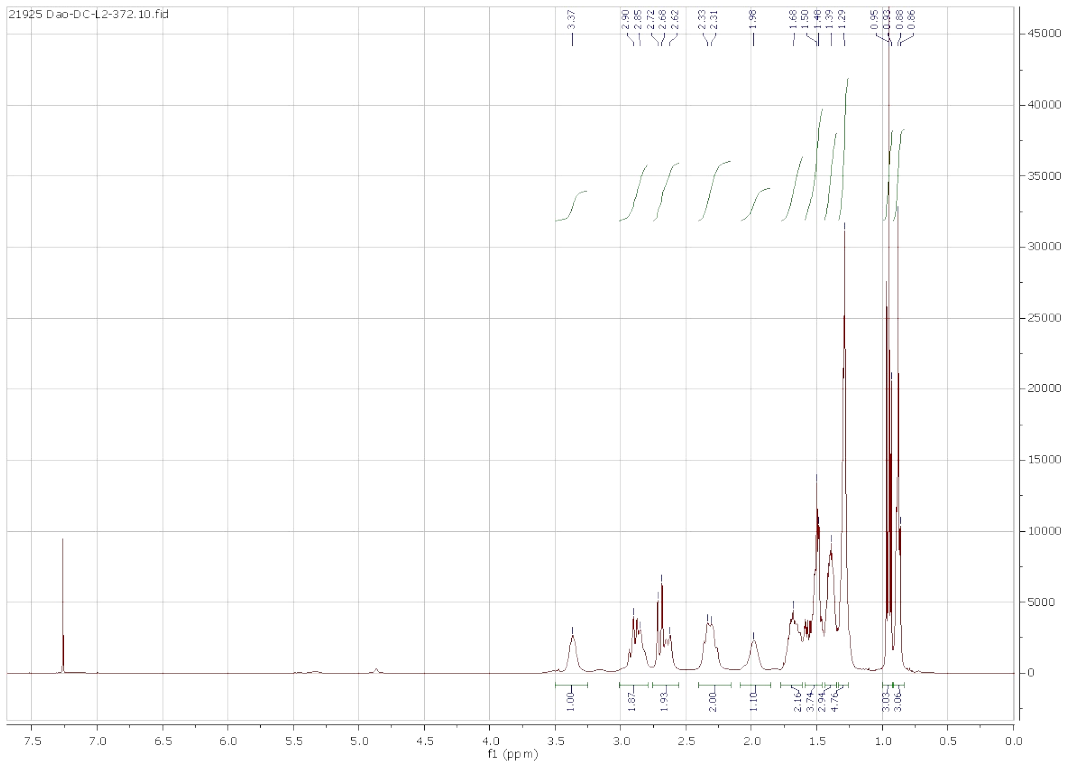

**Figure S39**: ${}^{1}$H NMR spectrum (500 MHz, $CDCl_3$) of **17**.

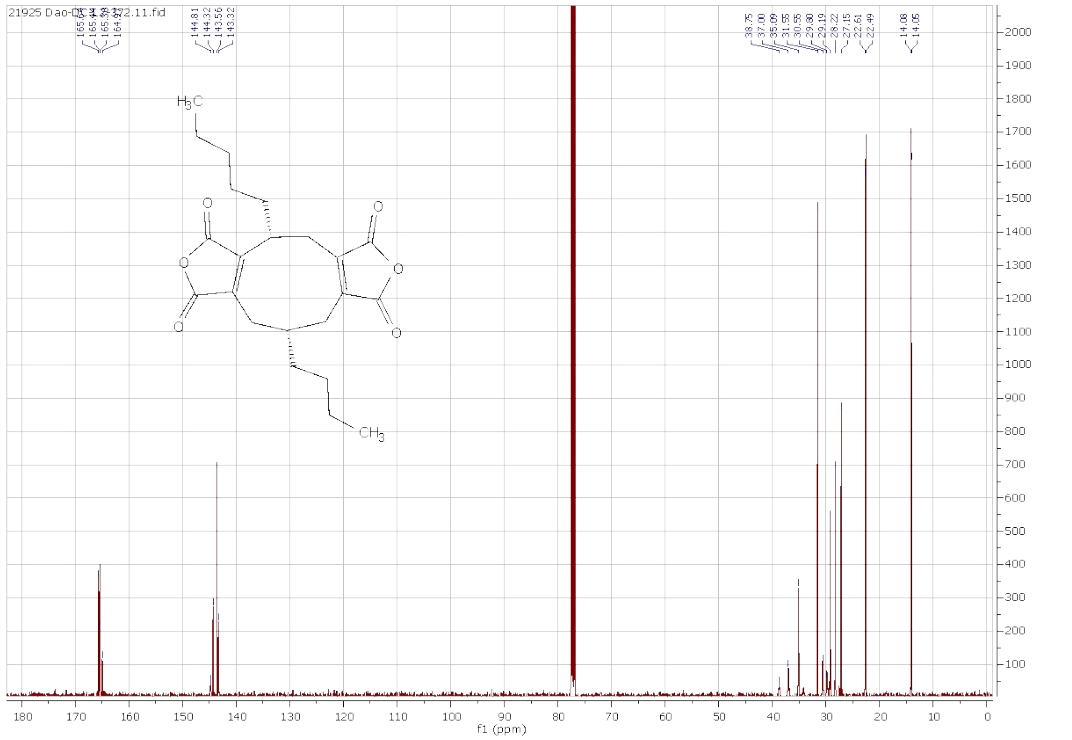

**Figure S40**: ${}^{13}$C NMR spectrum (125 MHz, $CDCl_3$) of **17**.

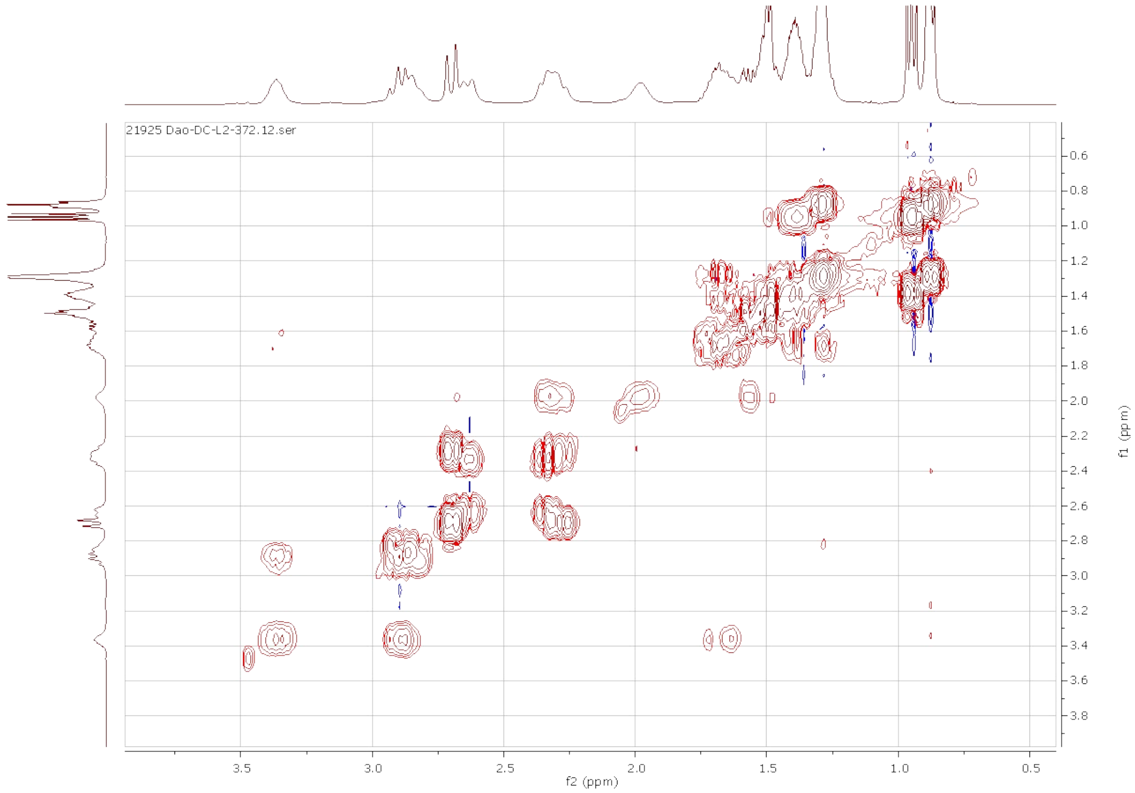


**Figure S41**: COSY spectrum of **17**.

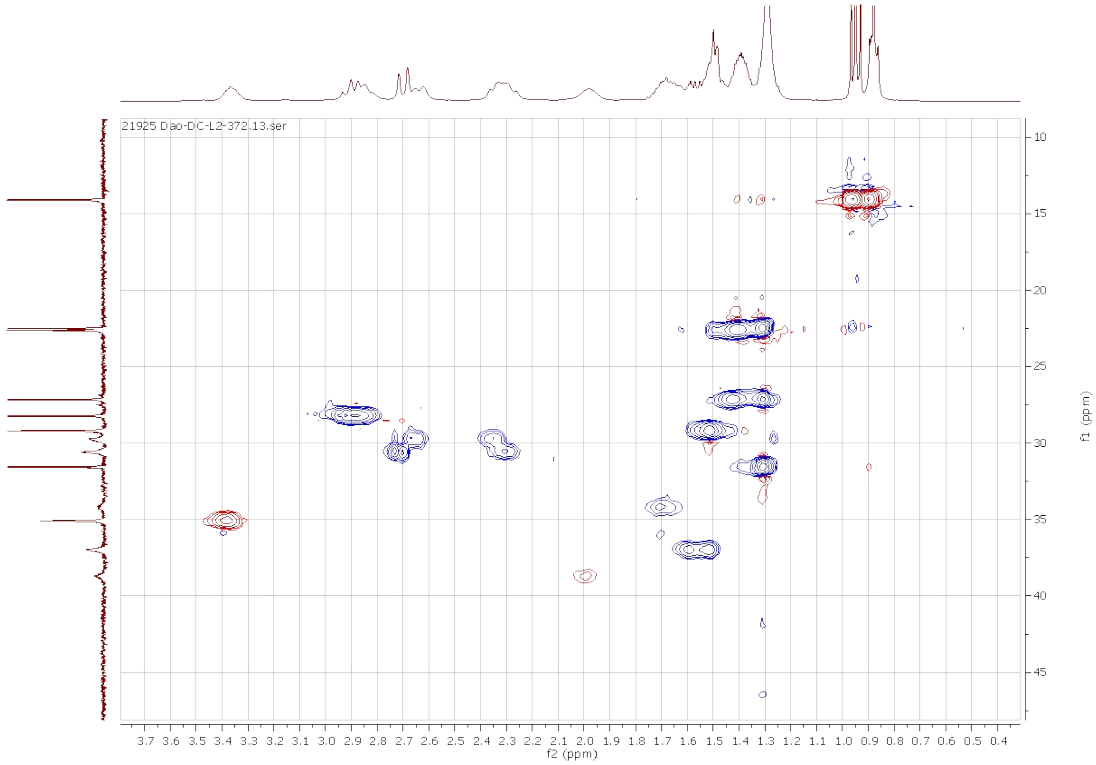


**Figure S42:** HSQC spectrum of **17**.

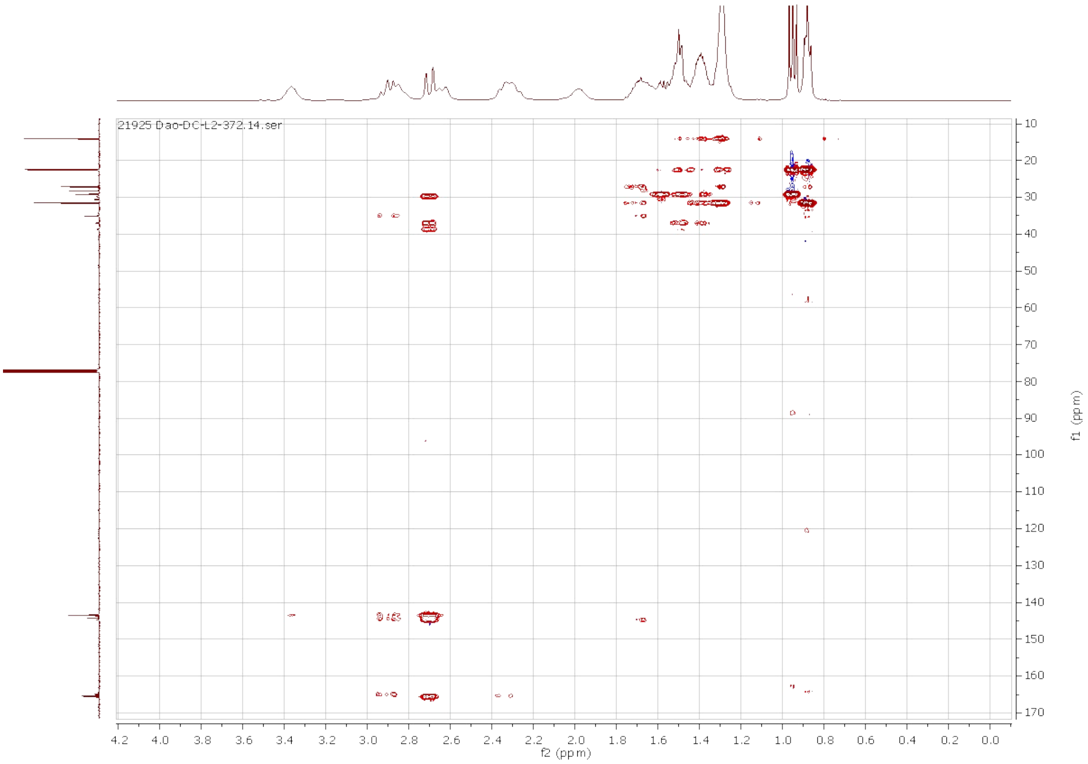


**Figure S43:** HMBC spectrum of **17**.

## NMR data for 5-hydroxy-deoxyscytalidin **19**

**Table S9**: NMR assignments for 5-hydroxy-deoxyscytalidin **19** ([a]500 MHz, [b]125 MHz in $CDCl_3$)

| | 5-hydroxy-deoxyscytalidin **19** | | |
|---|---|---|---|
| **Position** | $^{b}\delta_C$ | $^{a}\delta_H$, mult. (*J* in Hz) | HMBC |
| **1** | 35.1 | 3.34, m | 9 |
| **2** | 27.6 | 2.99, m<br>3.51, m | 1, 3 |
| **3** | 144.2 | - | |
| **4** | 144.4 | - | |
| **5** | 66.7 | 4.90, s | 4, 6, 7, 4', 1''' |
| **6** | 42.1 | 1.78, m | |
| **7** | 22.5 | 2.34, d (14.0)<br>3.14, m | 5, 6, 8, 8', 1''' |
| **8** | 144.0 | - | |
| **9** | 144.7 | - | |
| **3´** | 164.2 | - | |
| **4´** | 164.5 | - | |
| **8´** | 165.4 | - | |
| **9´** | 165.9 | - | |
| **1´´** | 36.1 | 1.69, m | |
| **2´´** | 26.9 | 1.35, m<br>1.44, m | |
| **3´´** | 31.6 | 1.30, m | |
| **4´´** | 22.6 | 1.32, m | |
| **5´´** | 14.08 | 0.90, t (7.0) | 4'', 3'' |
| **1´´´** | 34.8 | 1.63, m | |
| **2´´´** | 29.3 | 1.56, m | |
| **3´´´** | 22.7 | 1.40, m | |
| **4´´´** | 14.11 | 0.96 t (7.5) | 2''', 3''' |

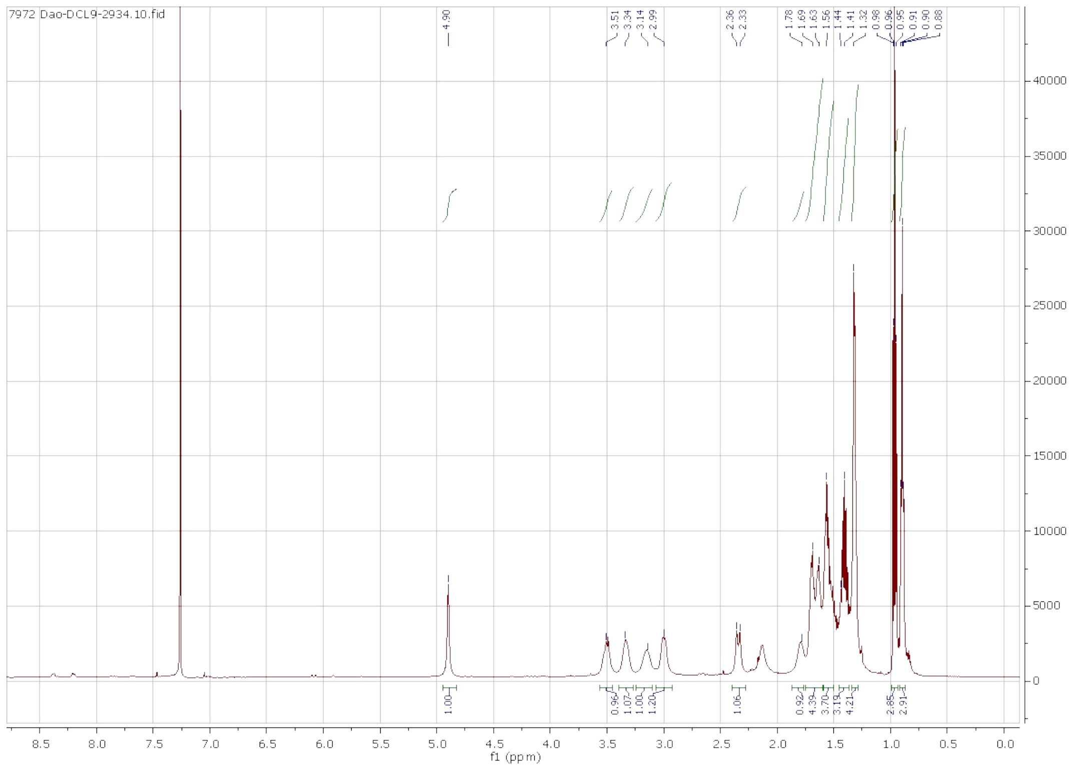

**Figure S44:** $^{1}$H NMR spectrum (500 MHz, $CDCl_3$) of **19**.

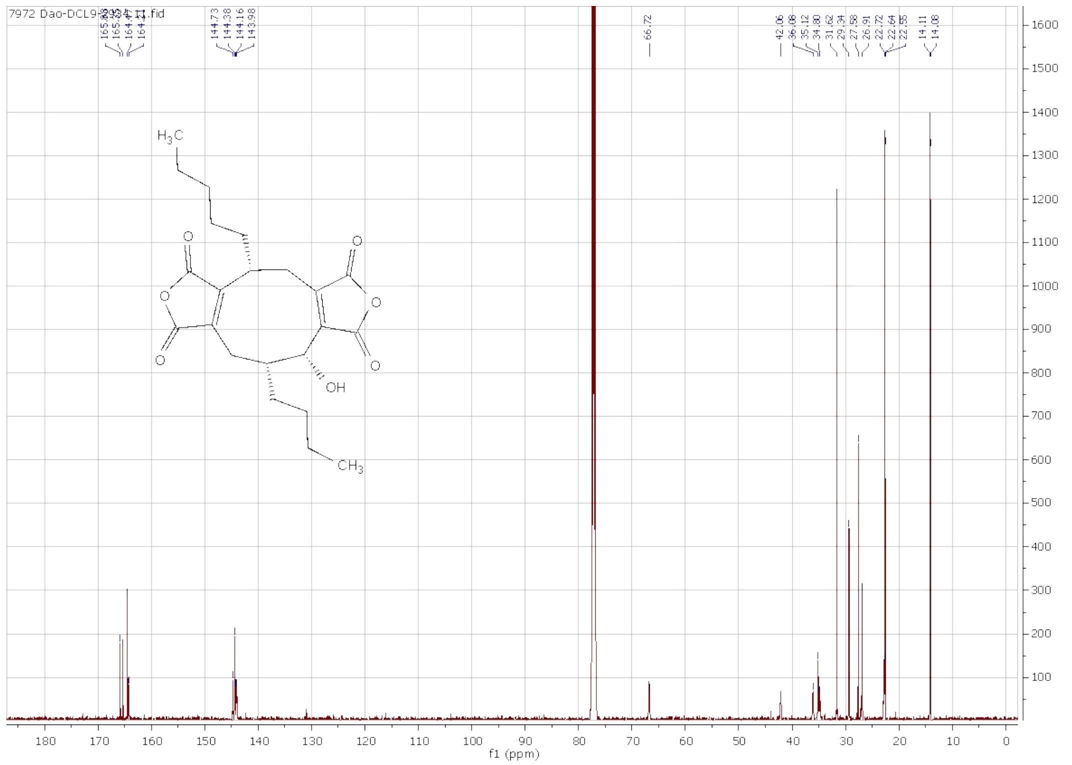

**Figure S45:** $^{13}$C NMR spectrum (125 MHz, $CDCl_3$) of **19**.

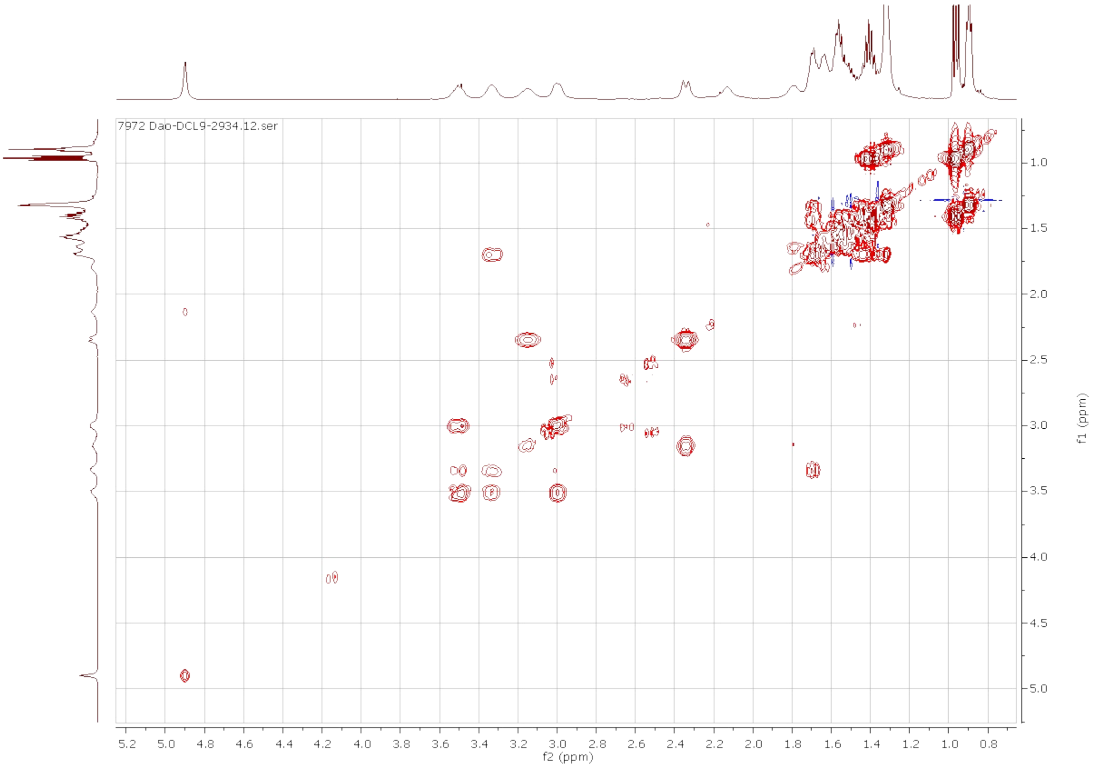


**Figure S46:** COSY spectrum of **19**.

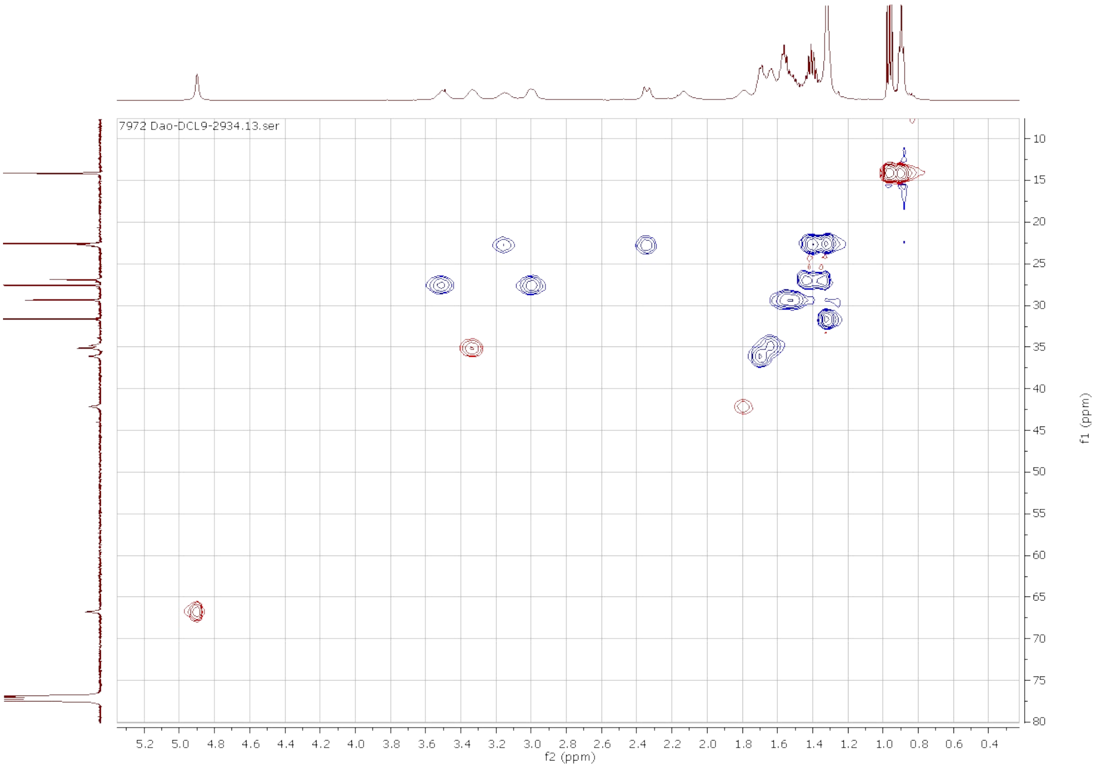


**Figure S47:** HSQC spectrum of **19**.

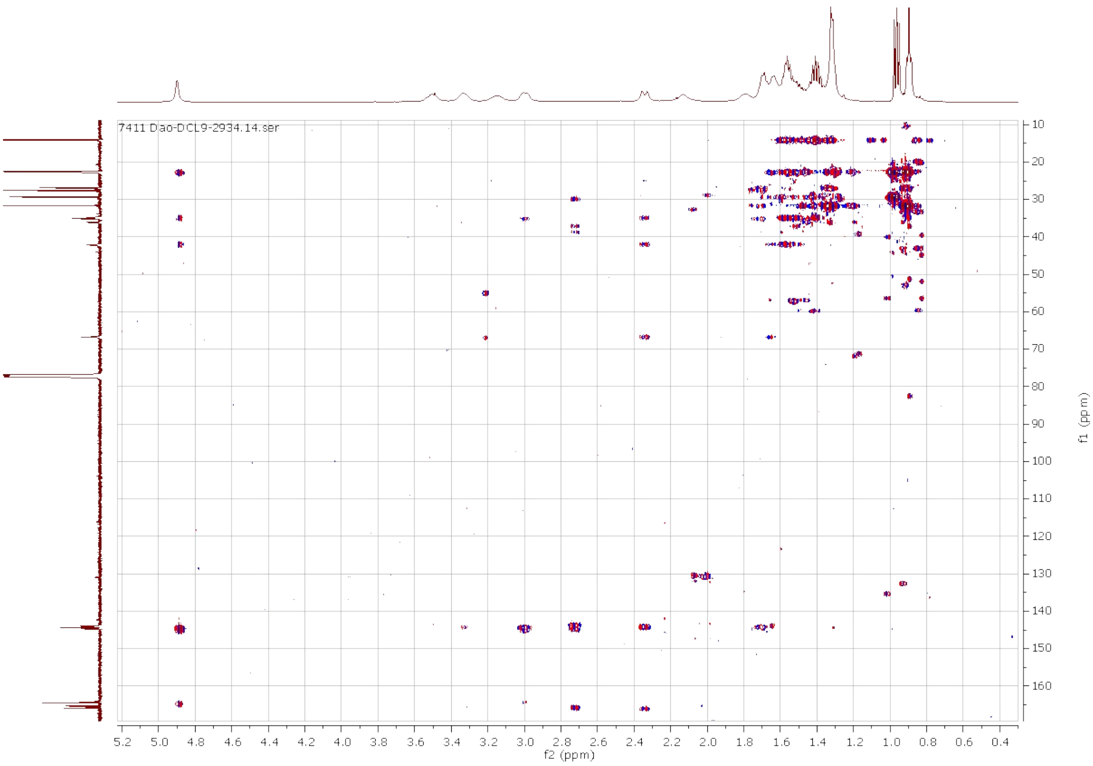


**Figure S48:** HMBC spectrum of **19**.

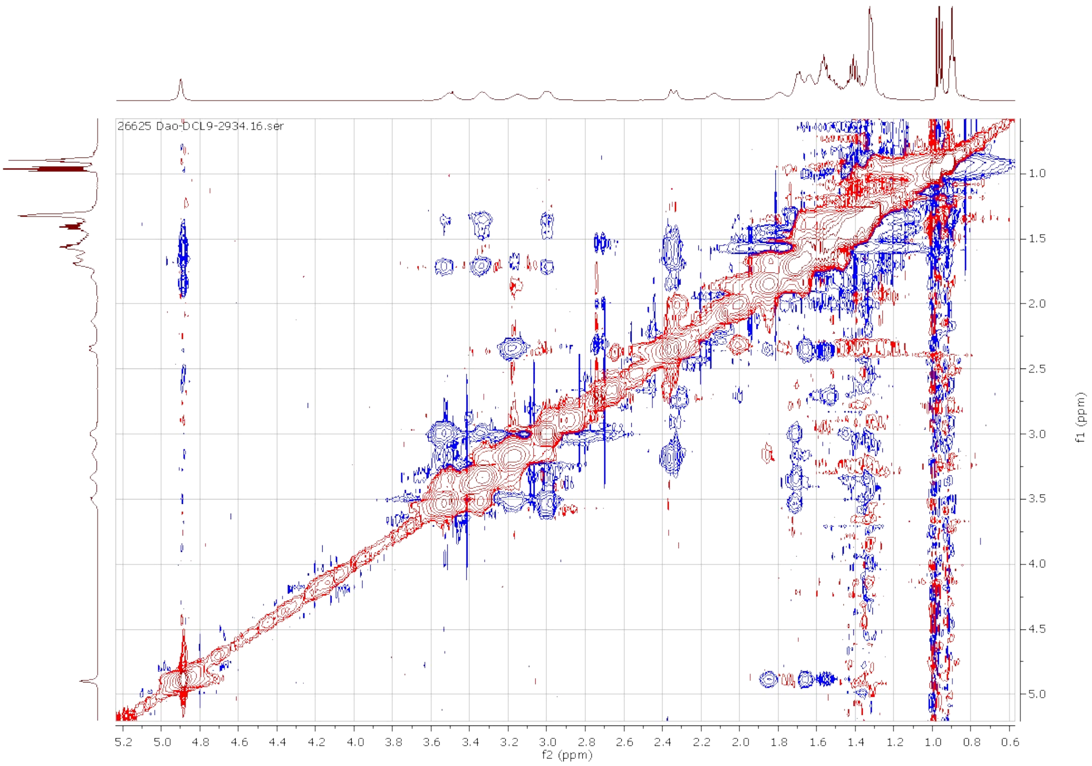


**Figure S49**: NOESY spectrum **19**.

## NMR data for 20 and 21

**Table S10**: NMR assignments for **20** and **21** ([a]500 MHz, [b]125 MHz in $CDCl_3$). Compound one is observed as a mixture of epimers. The data for the major compound is shown here.

| | 20 | | | 21 | | |
|---|---|---|---|---|---|---|
| **Position** | [b]$\delta_C$ | [a]$\delta_H$, mult. ($J$ in Hz) | HMBC | [b]$\delta_C$ | [a]$\delta_H$, mult. ($J$ in Hz) | HMBC |
| **1** | 37.0 | 2.98, m | 2, 3, 8, 9, 9' | 36.9 | 2.94, m | 2, 9, 9', 1'' |
| **2** | 28.0 | 2.83, dd (13.5, 4.2)<br>3.29, dd (14.0, 10.6) | 1, 3, 4, 9, 3', 1'' | 28.8 | 2.67, m<br>3.47, m | 1, 3, 9, 3', 1'' |
| **3** | 142.7 | - | | 140.0 | - | |
| **4** | 143.4 | - | | 147.0 | - | |
| **5** | 55.4 | 4.50, d (15.3)<br>4.60, d (15.3) | 4, 4' | 170.0 | - | |
| **6** | 204.3 | - | | 208.8 | - | |
| **7** | 37.3 | 3.40, d (17.0)<br>3.65, d (17.0) | 6, 8, 9, 8' | 37.2 | 3.40, m<br>3.75, m | 6, 8, 9, 8' |
| **8** | 139.6 | - | | 138.2 | - | |
| **9** | 147.5 | - | | 148.6 | - | |
| **3´** | 166.2 | - | | 170.0 | - | |
| **4´** | 164.6 | - | | 97.3 | 6.19, s | 3, 4, 3' |
| **8´** | 164.6 | - | | 164.8 | - | |
| **9´** | 164.3 | - | | 163.4 | - | |
| **1´´** | 33.3 | 1.77, m<br>1.90, m | 2'', 3'' | 33.5 | 1.83, m<br>1.98, m | 1, 9, 2'', 3'' |
| **2´´** | 31.5 | 1.25 – 1.36, m | | 31.5 | 1.28, m | |
| **3´´** | 27.6 | 1.25 – 1.36, m | | 27.6 | 1.19, m<br>1.27, m | |
| **4´´** | 22.2 | 1.25 – 1.36, m | | 22.4 | 1.33, m | |
| **5´´** | 13.9 | 0.88, t (6.9) | 3'' | 13.7 | 0.87, t (6.9) | |
| **1´´´** | 43.3 | 2.54, td (7.5, 3.3) | 6, 2''', 3''' | 43.4 | 2.63, m | 6, 2''', 3''' |
| **2´´´** | 25.7 | 1.58, quint. (7.5) | 6, 1''', 3''', 4''' | 25.4 | 1.56, quint (7.5) | 6, 1''', 3''', 4''' |
| **3´´´** | 22.2 | 1.25 – 1.36, m | | 22.0 | 1.33, m | |
| **4´´´** | 14.1 | 0.92, t (7.3) | 2´´´ | 14.0 | 0.92, t (7.2) | |

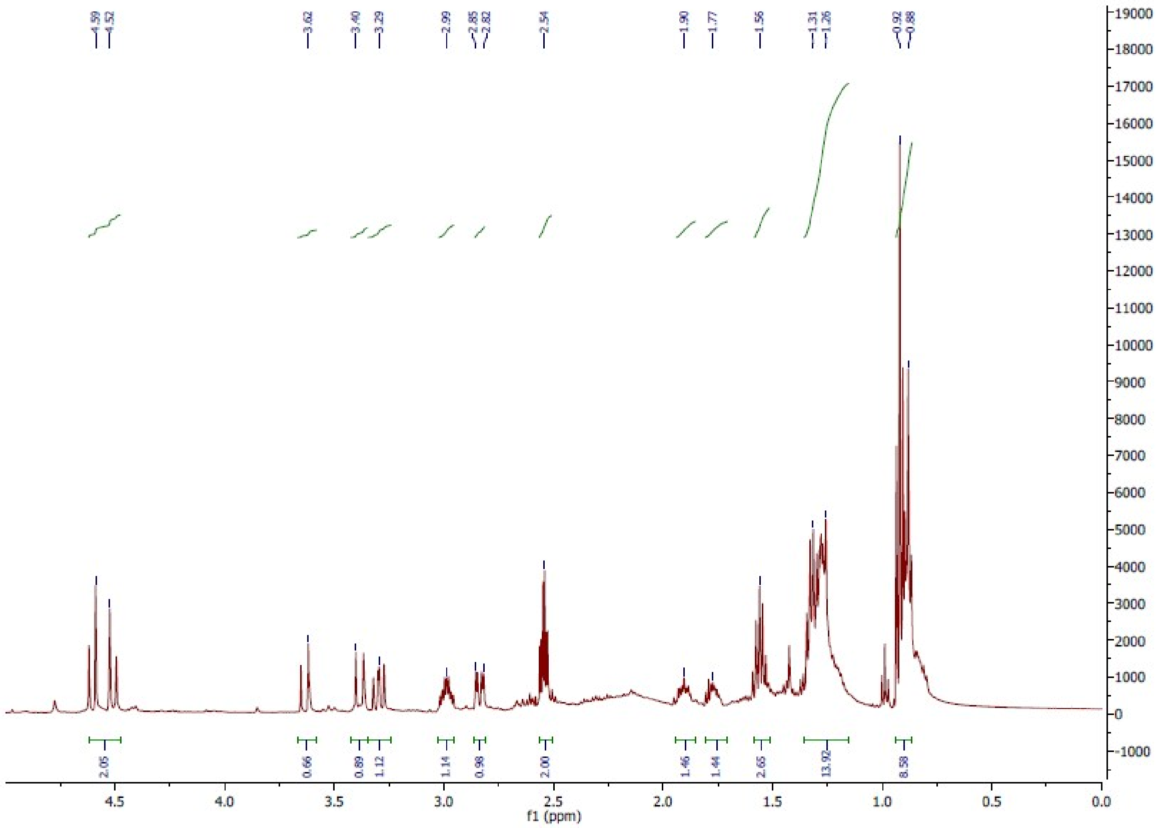


**Figure S50**: $^{1}$H NMR spectrum (500 MHz, $CDCl_3$) of **20**.

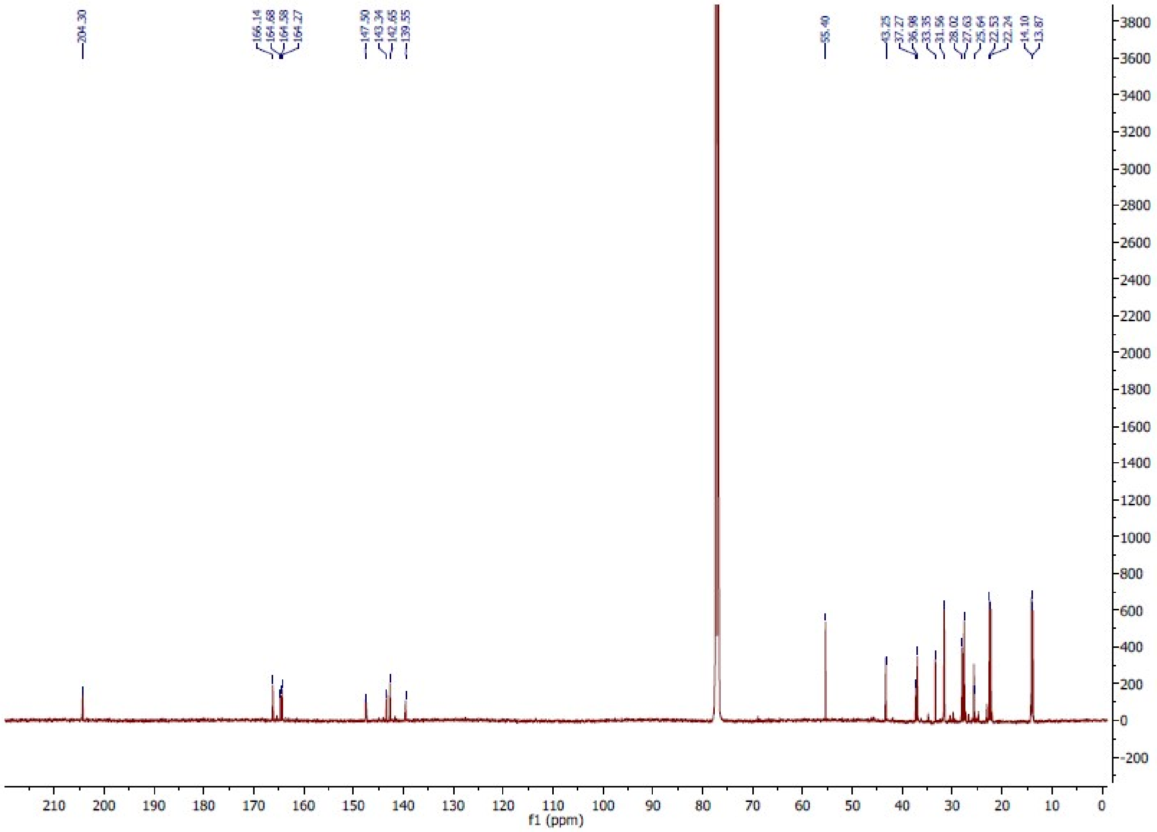


**Figure S51:** $^{13}$C NMR spectrum (125 MHz, $CDCl_3$) of **20**.

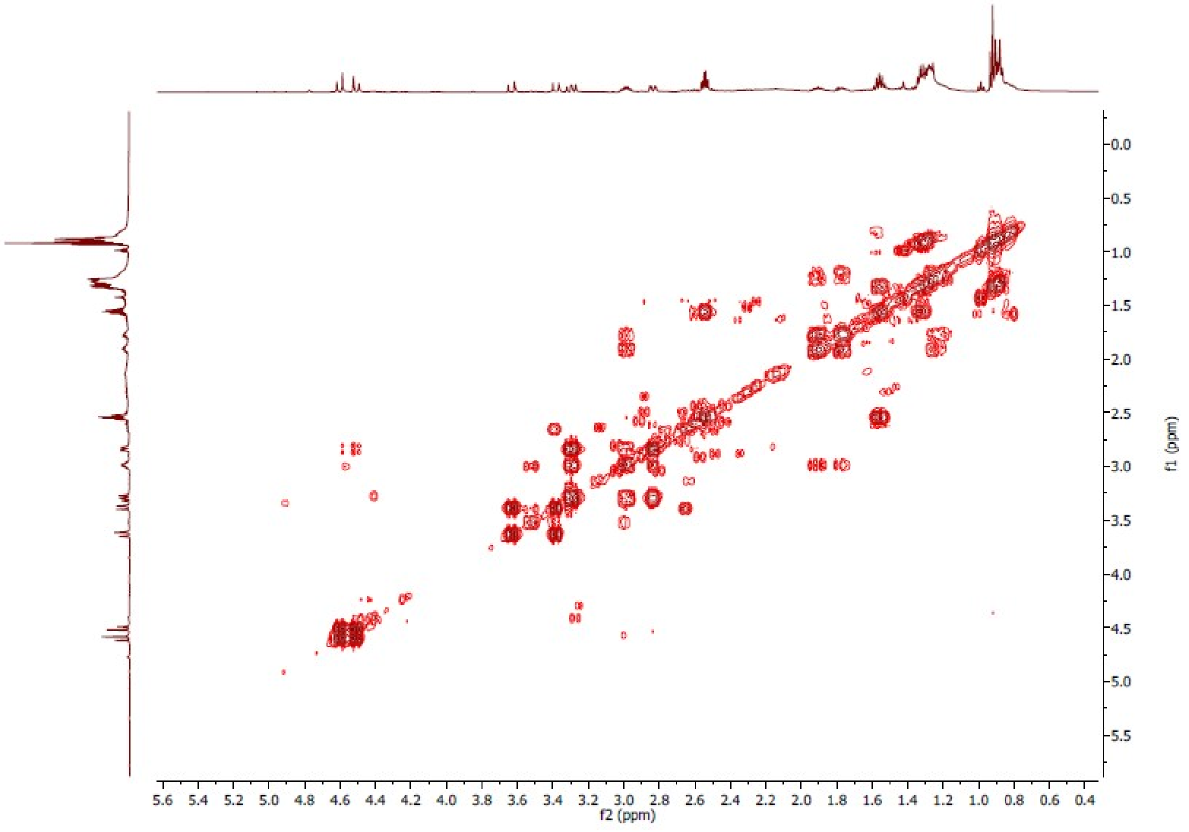


**Figure S52**: COSY spectrum of **20**.

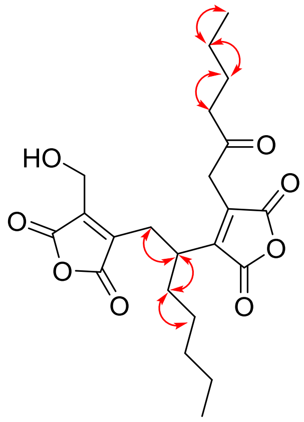


**Figure S53**: Key COSY correlations of **20**.

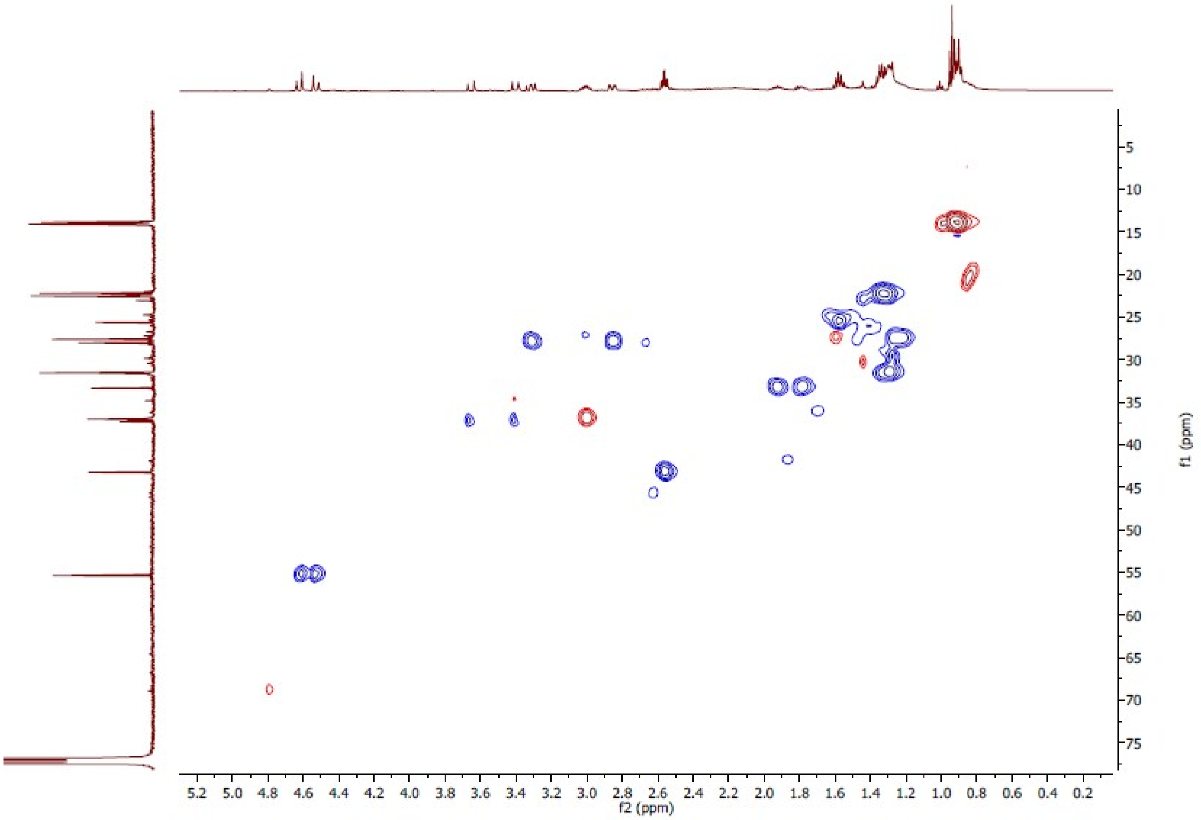


**Figure S54**: HSQC spectrum of **20**.

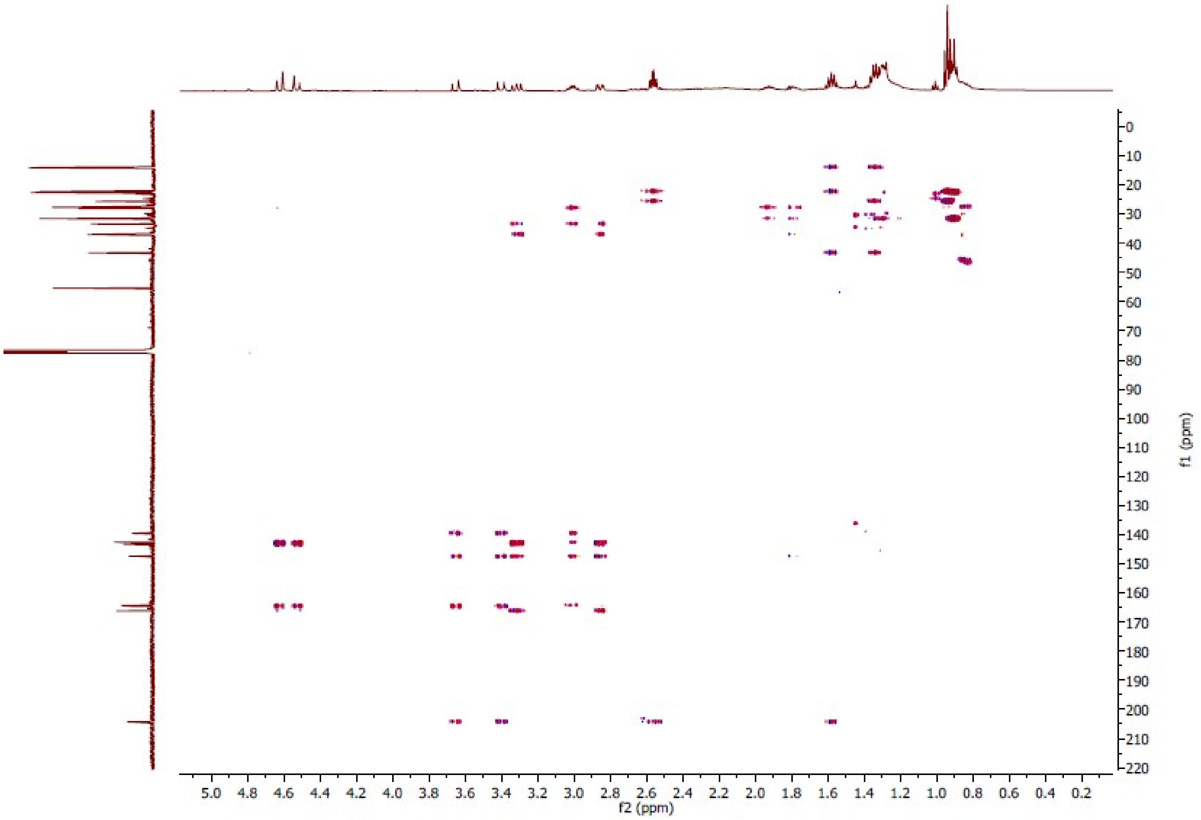


**Figure S55**: HMBC spectrum of **20**.

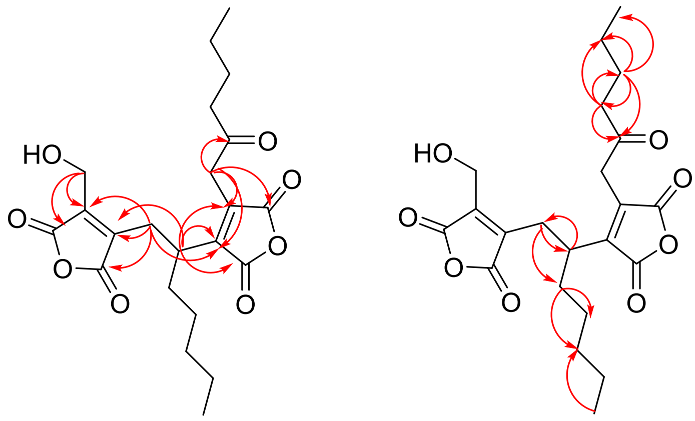


**Figure S56**: Key HMBC correlations for **20**.

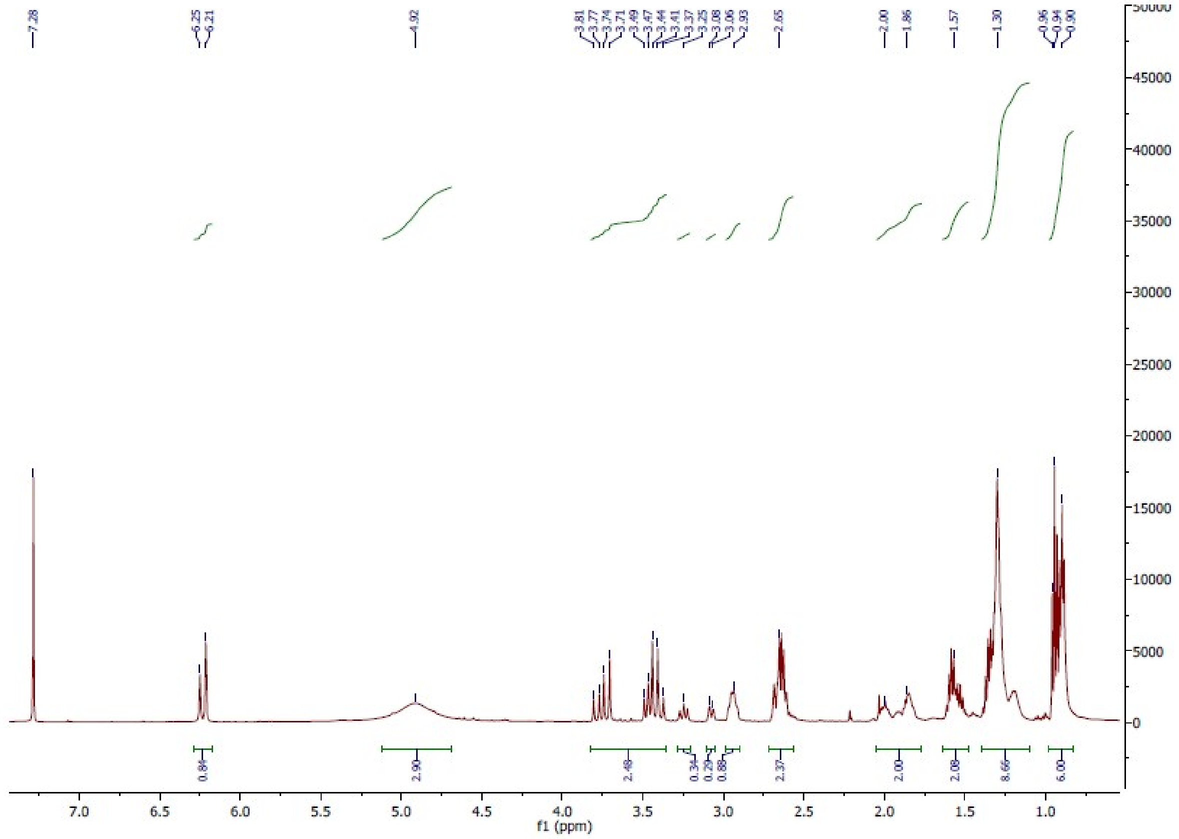


**Figure S57:** $^{1}$H NMR spectrum (500 MHz, $CDCl_3$) of **21**.

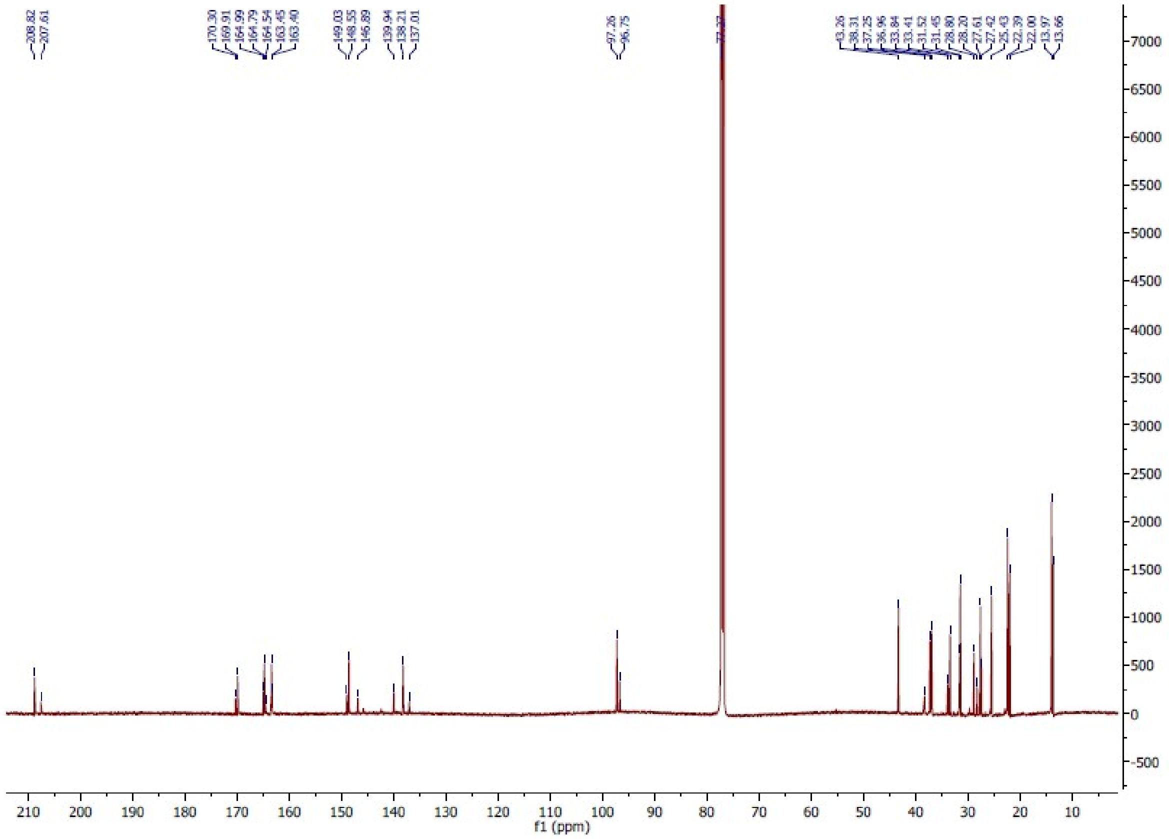

**Figure S58:** $^{13}C$ NMR spectrum (125 MHz, $CDCl_3$) of **21**.

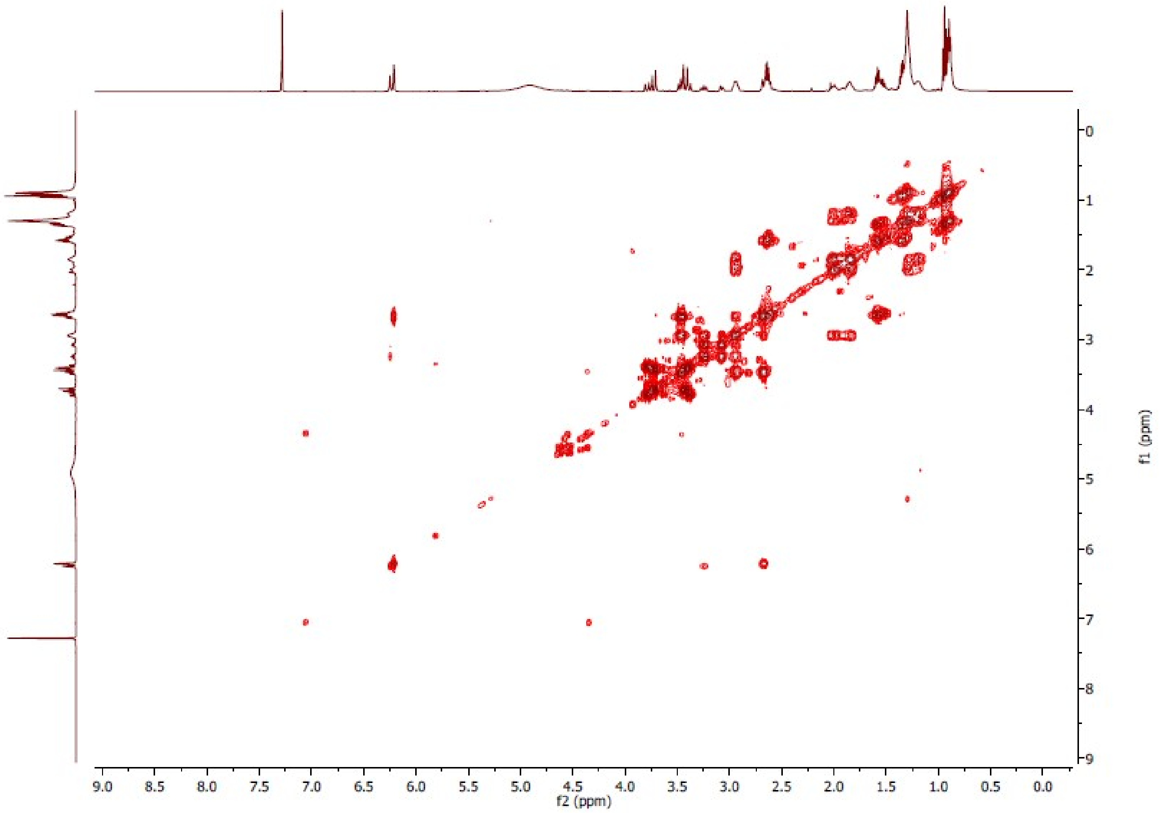

**Figure S59:** COSY spectrum of **21**.

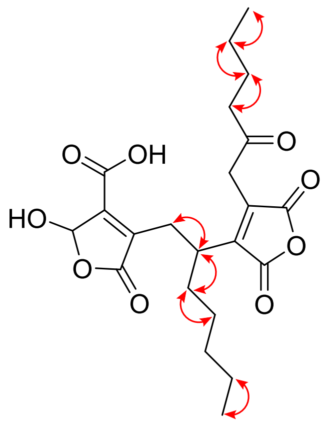


**Figure S60**: Key COSY correlations of **21**.

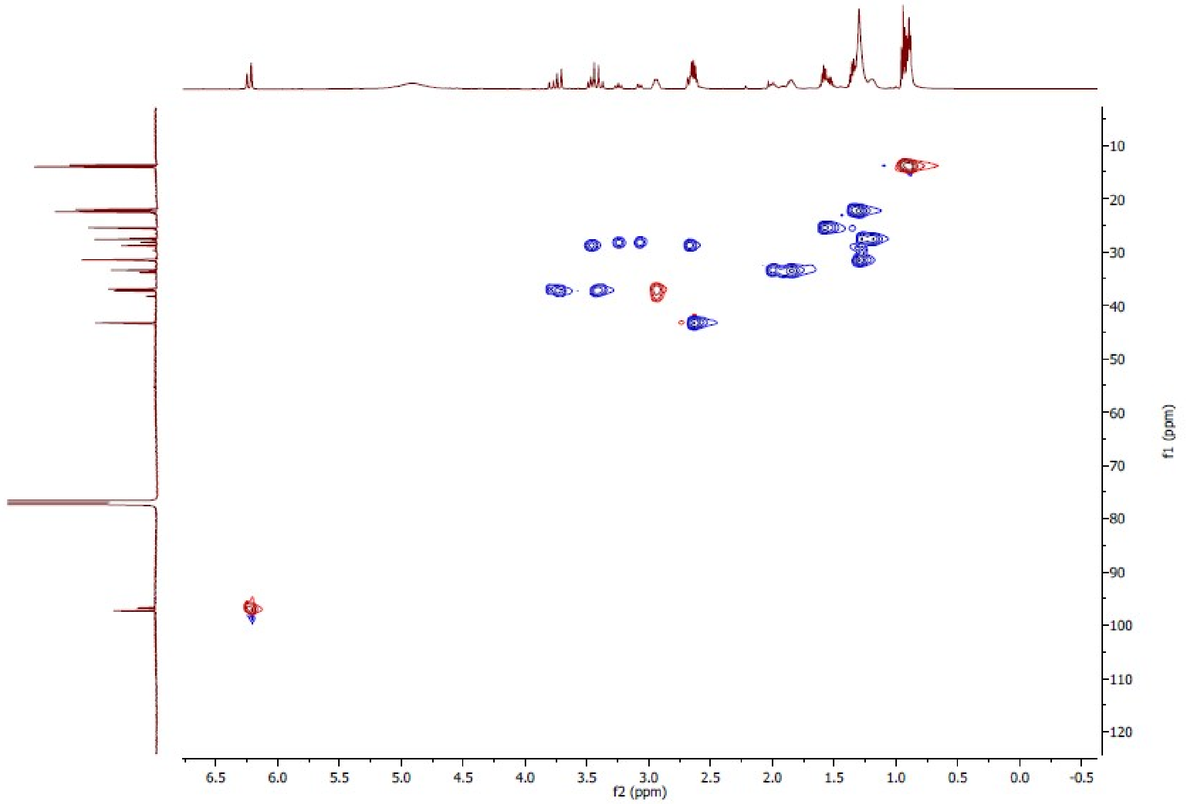


**Figure S61:** HSQC spectrum of **21**.

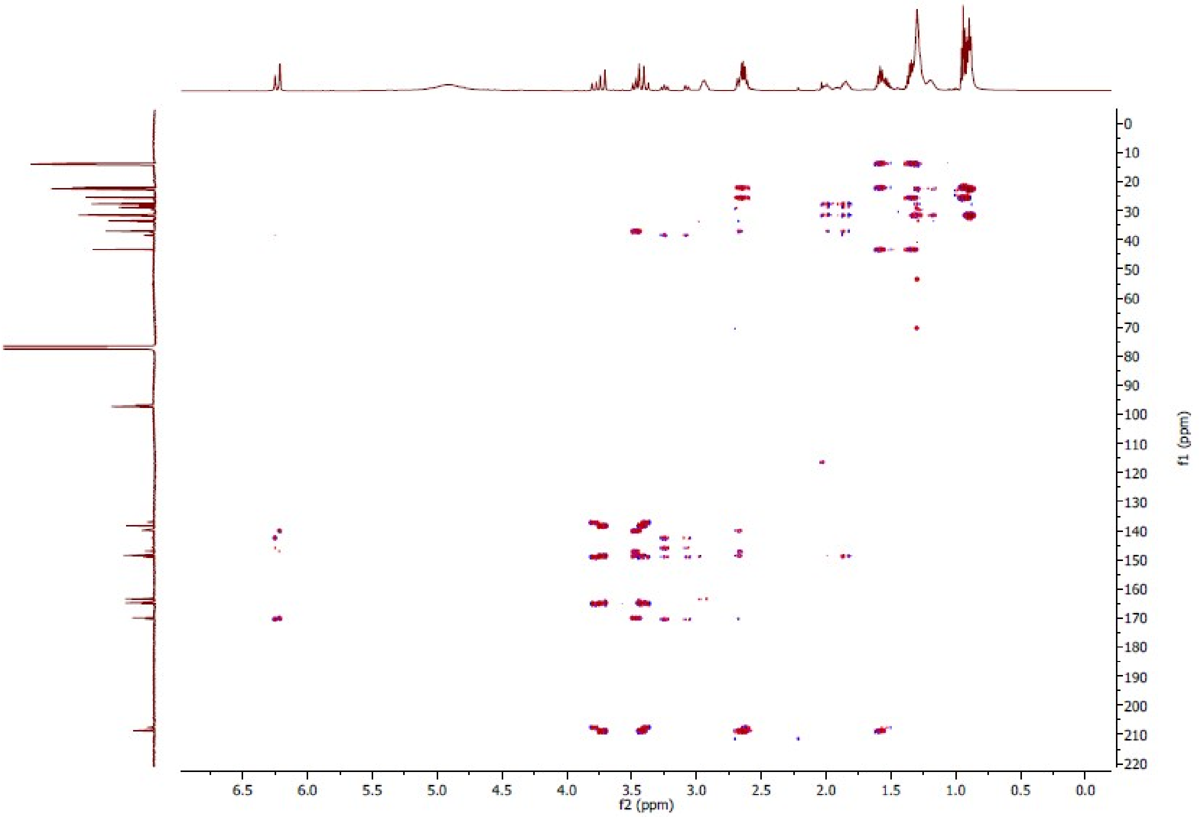


**Figure S62**: HMBC spectrum of **21**.

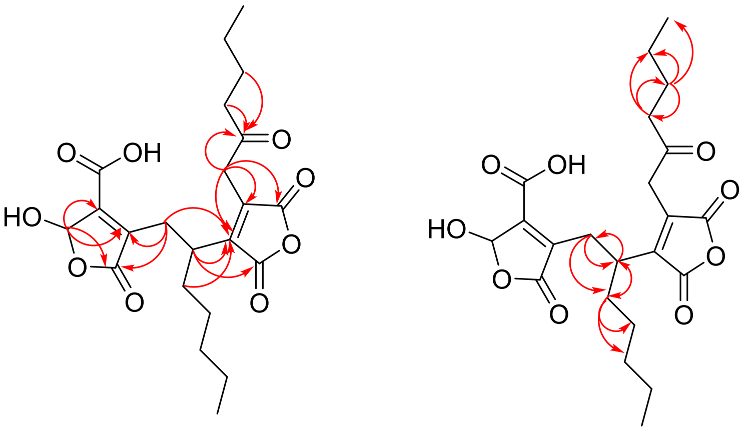

**Figure S63**: HMBC correlations of **21**.

## NMR data for deoxyzopfiellin **24**

**Table S11**: NMR assignments for deoxyzopfiellin **24** (pyridine-d$_5$, 500 MHz) -sample run in pyridine-d$_5$ to enable direct comparison with the data of Minami, Oikawa and co-workers (*Org. Lett.*, 2020, **22**, 1997-201)

| | **Deoxyzopfiellin 24** | |
|---|---|---|
| **Position** | $\delta_C$ | $\delta_H$, mult. (*J* in Hz) |
| **1** | 37.58 | 3.34 (m) |
| **2** | 25.41 | 3.41 (m)/3.21 (m) |
| **3** | 141.62 | |
| **4** | 147.88 | |
| **5** | 37.52 | 3.34 (m) |
| **6** | 25.41 | 3.41 (m)/3.21 (m) |
| **7** | 141.62 | |
| **8** | 147.88 | |
| **3´** | 167.14 | |
| **4´** | 166.81 | |
| **7´** | 167.14 | |
| **8´** | 166.83 | |
| **1´´** | 34.67 | 1.89 (m)/1.69 (m) |
| **2´´** | 29.22 | 1.43 (m) |
| **3´´** | 32.10 | 1.26 (m) |
| **4´´** | 23.07 | 1.27 (m) |
| **5´´** | 14.53 | 0.85 (m) |
| **1´´´** | 34.96 | 1.89 (m)/1.69 (m) |
| **2´´´** | 26.91 | 1.47 (m) |
| **3´´´** | 23.01 | 1.26 (m) |
| **4´´´** | 14.30 | 0.84 (m) |

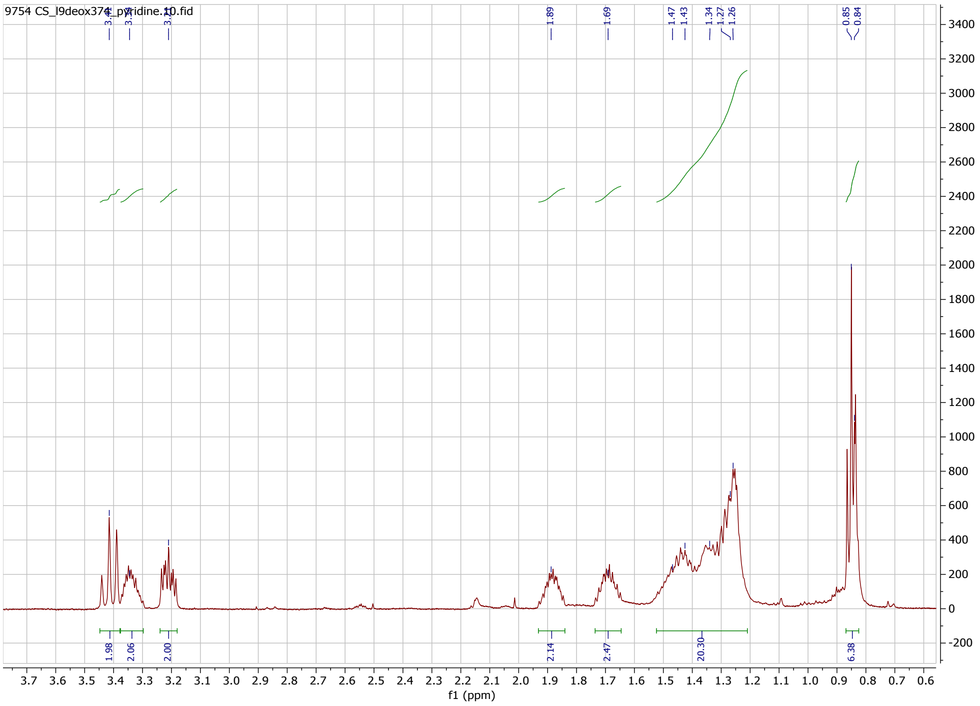

**Figure S64**: $^{1}H$ NMR spectrum (500 MHz, pyridine-$d_5$) of **24**.

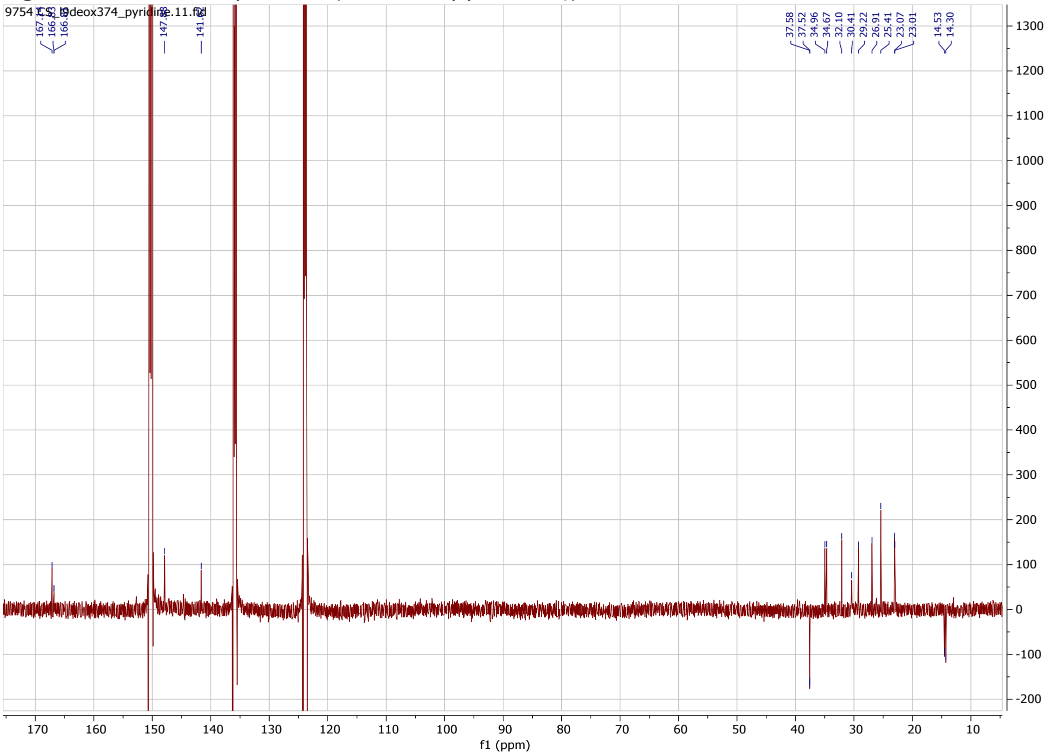


**Figure S65:** $^{13}C$ NMR spectrum (125 MHz, pyridine-$d_5$) of **24**.

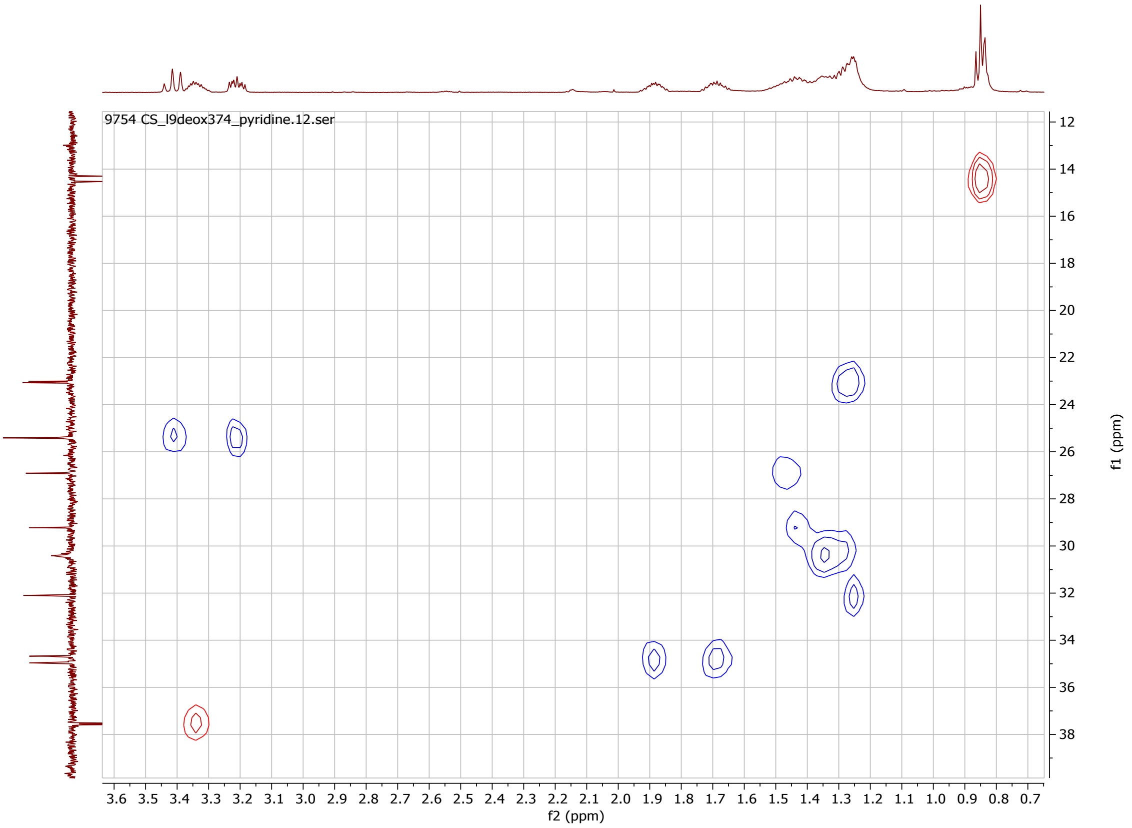


**Figure S66**: HSQC spectrum of **24**.

## *In vitro* assays with ZopL9

### Protein production

The coding sequence for *zopL9* was ordered as a codon optimised synthetic gene (Figure SX), cloned into the expression vector pET151 (purchased from ThermoFisher scientific). The resulting plasmid; pET151-zopL9, was then transformed into Agilent BL21 gold cells according to the manufacturers protocol. A single colony was used to inoculate LB media (4 x 800 mL flasks) supplemented with 100 µg/ mL ampicillin. When the $OD_{600}$ reached 0.5 - 0.7 protein expression was induced by the addition of IPTG (100 µL, 1 M stock). The cells were incubated overnight, then collected and resuspended in column buffer A (20 mM Tris pH 7.9, 10 mM imidazole, 500 mM NaCl, 10 % glycerol). Sonication was used to disrupt the cells before centrifugation at 15,000 rpm for 30 minutes. The supernatant was loaded onto a Nickel column (GE Healthcare HiTrap 5 mL) and eluted with column buffer A. The target protein was collected by eluting with column buffer B (20 mM Tris-HCl pH 7.9, 500 mM imidazole, 500 mM NaCl, 10 % glycerol). ZopL9 was purified using size exclusion chromatography and eluted into storage buffer (50 mM Tris-HCl pH 8, 20 % glycerol). Mass spectrometry and SDS page analysis confirmed the protein had the expected mass.

```
>ZopL9-CO
ATGGCAACCGCAACCGTTACCACCGCACCGACCGTTGTTCGCACCACCGCAGATTATTATGATGCACCGCCTGTT
CTGAAAATCCATACCTATACACGTGAAAGCTATGAAGAACAGTTTGGCAACAAAAGCGTTATCCATCATCCGATC
AACCTGAAAGATATTCGTGCAGCAAACATTAACCTGCAGAATAATGGTTTCCAGCTGATTAAACTGCAGAGCAAA
CTGACCAATCCGGATGATTATCTGGATGAAGAAACCGTGAAACGTGTGTATATTCCGGAACTGGCAGAAGCAGTT
AAAAAACTGACCGGTGCAACCGAAGTTCGTGTTCTGAATCCGAAAGTTCGTGATAGCAGCACCGAAAAAGATGGC
TTTGAAAATAACTGGGGCAAAAATAACGGTGCCGTTCGTCGTATTCATATTGATCTGGCACCTGGTGGTGTTGAA
GAAGCACTGTATCCGATTTTTGGCGAAGAGTACATGAAAAGCATTGCAGGTCGTTGGCGTCTGATTAATGCATGG
AAACCGACACGTCCGGTTGAACGTGATCCGCTGGCAGTTTGTGATCGTGTTCCGGATGAAGATCTGGTTCCTCTG
CAGCGTGTTGTTCCGGGTAAAGCACTGATGGAACAGCGTTATCATCTGAAAACCGGCAAAAAAGATCACGATTGG
TATTATGCAAGCAATCAGCAGCCTGATGAAGTTCTGCTGTTTACCCAGTATAGCGATTTTCCGAATCGTAATACC
GCAGATCGTGTGCCGCATGTTAGCGTTAAACTGCCTGGTCAAGAAGATAAACCGCGTCGTACCAGCGTTGATGCA
CGTTGTCTGGTTGTTTGGTAA
```

**Figure S67**: *zopL9* codon optimised for expression in *E. coli*. Optimised and using the ThermoFisher GeneArt service.

### In vitro assays

*In vitro* assays with ZopL9 were conducted in 200 µL reaction mixtures containing 50 mM PBS buffer (pH 7.8), 0.1 mM of substrate (either **17** or **19**), 1.0 mM $(NH_4)_2Fe(SO_4)_2$, 10 mM α-ketoglutarate, 8 mM ascorbic acid and 10 µM ZopL9. After incubation at 30 °C for 18 hours, 200 µL of MeOH was added to terminate the reaction. Protein was removed by centrifugation and the supernatant was extracted with EtOAc (2 x 400 µL). The extract was dried and dissolved in MeCN prior to LCMS analysis.

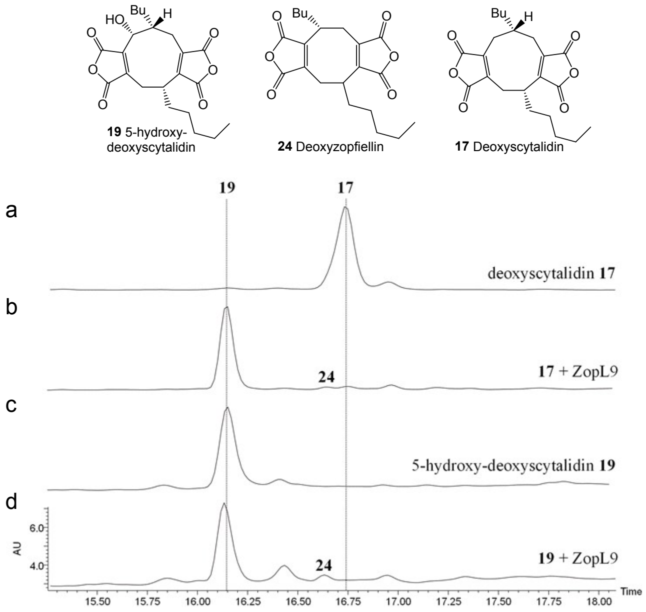


**Figure S68**: *In vitro* assays using purified ZopL9 and either deoxyscytalidin **17** or 5-hydroxy-deoxyscytalidin **19** as substrates. An efficient and total conversion of **17** to **19** can be seen (a,b), as well as the conversion – albeit at a lower level - of 5-hydroxy-deoxyscytalidin **19** to deoxyzopfiellin **24** (c,d).

## Labelling studies

### *S. album* UAMH 3620 $^{13}C$ labelling

Sodium [1,2- $^{13}C_2$]-acetate (100 mg in 5 mL deionised $H_2O$) was added to PDB (5 x 100 mL) on days 5 and 7. After 12 days the cultures were extracted to isolate labelled scytalidin and deoxyscytalidin. The labelling patterns for scytalidin and deoxyscytalidin were the same and were in agreement with the predicted biosynthetic pathway (Figure 3, main manuscript).

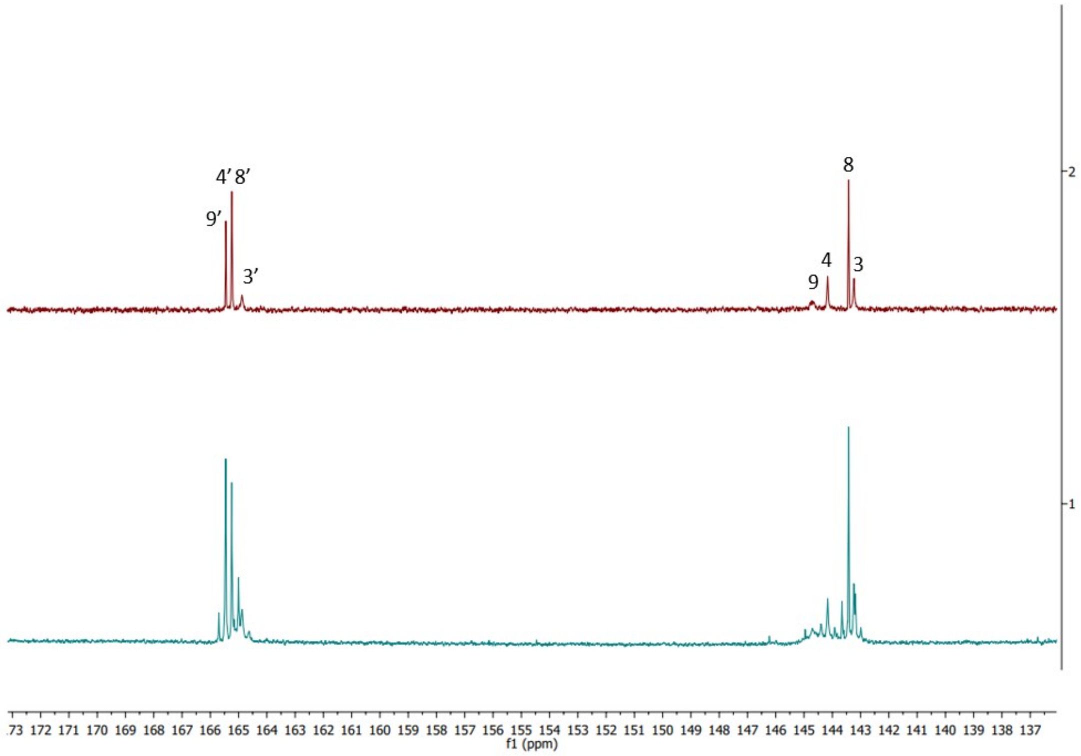


**Figure S69**: $^{13}C$ NMR of unlabelled (top) and labelled (bottom) deoxyscytalidin **17** from [1,2-$^{13}C_2$] acetate (125 MHz, $CDCl_3$)

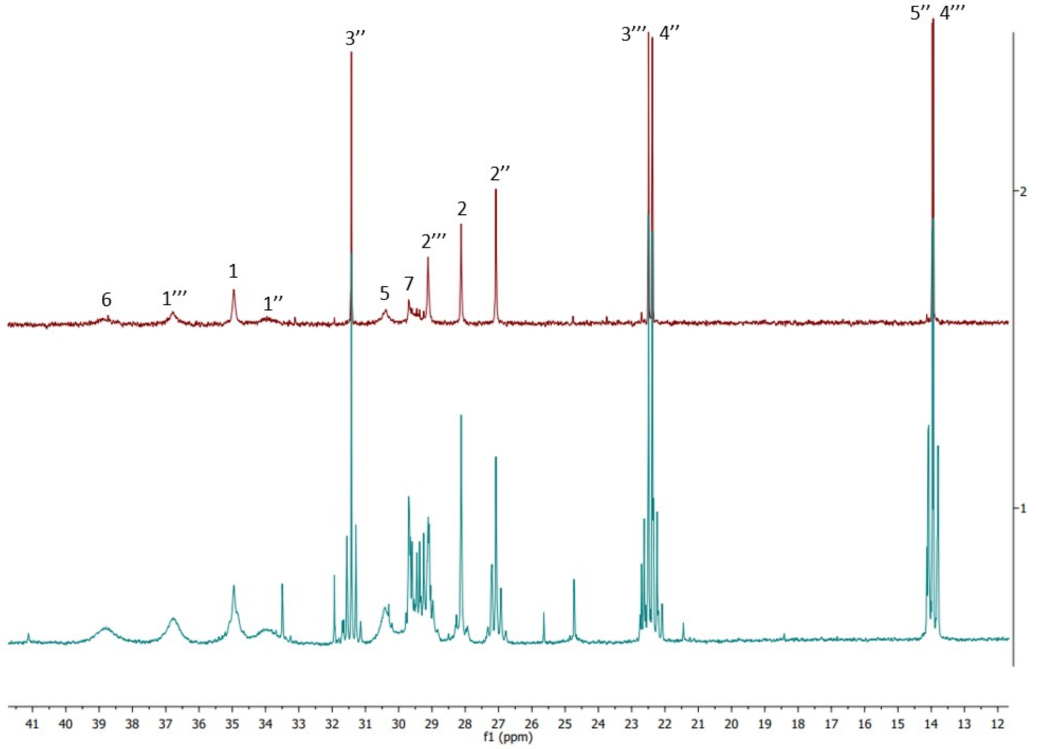


**Figure S70**: $^{13}C$ NMR of unlabelled (top) and labelled (bottom) deoxyscytalidin **17** from [1,2-$^{13}C_2$] acetate (125 MHz, $CDCl_3$)

## Zopfiellin $^{13}C$ labelling

Sodium [1,2-$^{13}C_2$]-acetate (50 mg in 6 mL of deionised $H_2O$) was added to PDB (3 × 100 mL) *D. curvata* cultures on days 4 and 5. After 14 days the culture was extracted and labelled zopfiellin **15** was purified. The samples were analysed by $^{13}C$ NMR and compared to the unlabelled zopfiellin **15** to determine the level and position of the incorporation. The carbon signals assigned to C-2 and C-6 were enhanced and appeared as singlets. Two carbonyl carbons, C-3' and C-7', were doublets ($J_{CC}$ 61 Hz) coupled to C-3 and C-7 respectively. One side of the molecule has four intact acetates (from C-8' to C-5''), suggesting that the original polyketide is a tetraketide. The other side of the molecule has three intact acetates (C- 4'/C-4; C-1'''/C-2'''; C-3'''/C-4'''). C-5 was labelled, but not coupled, suggesting the loss of one carbon.

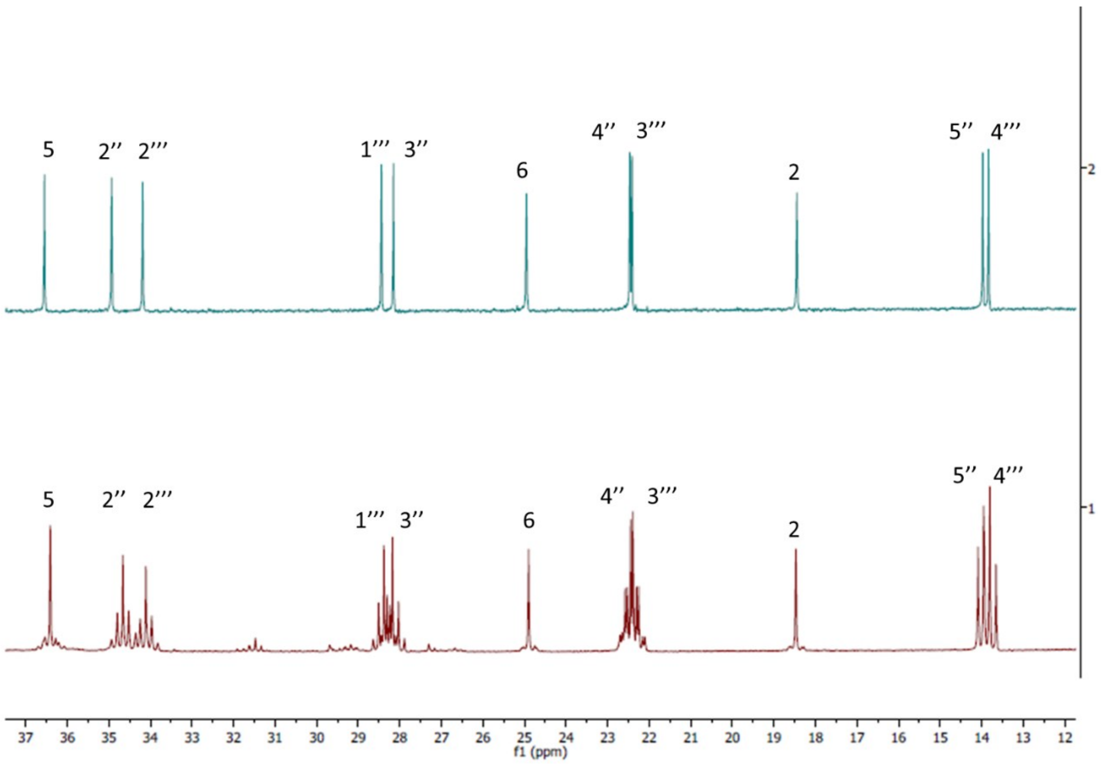


**Figure S71**: $^{13}C$ NMR of unlabelled (top) and labelled (bottom) zopfiellin **15** from [1,2-$^{13}C_2$] acetate (125 MHz, $CDCl_3$)

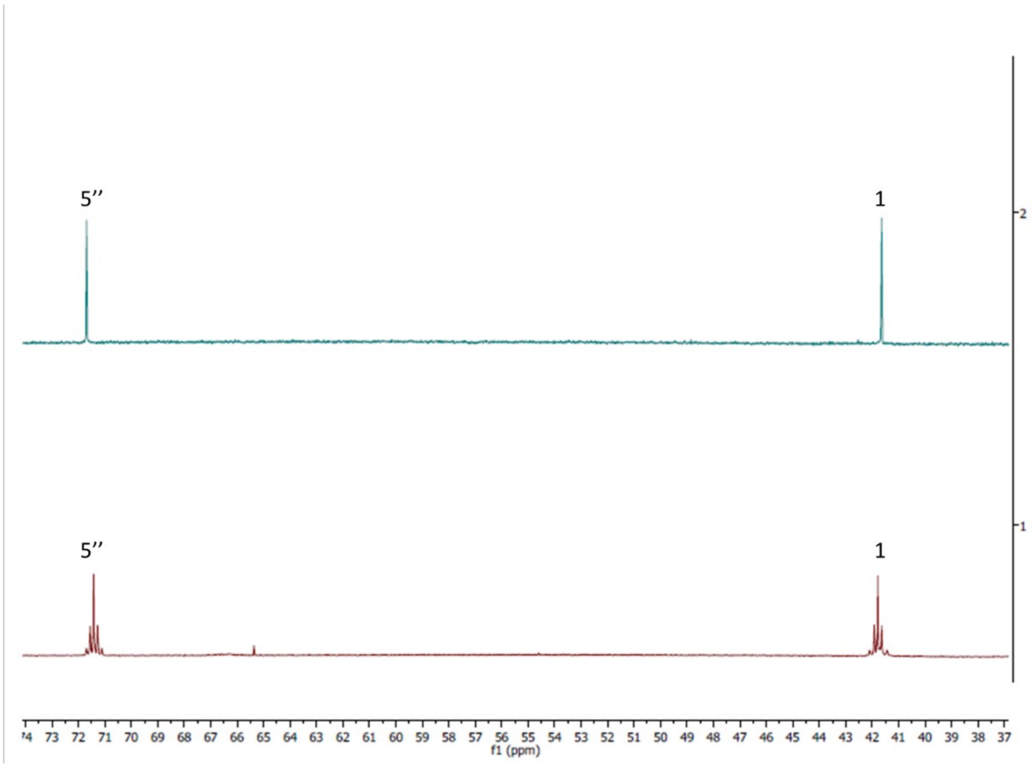


**Figure S72:** $^{13}C$ NMR of unlabelled (top) and labelled (bottom) zopfiellin **15** from [1,2-$^{13}C_2$] acetate (125 MHz, $CDCl_3$)

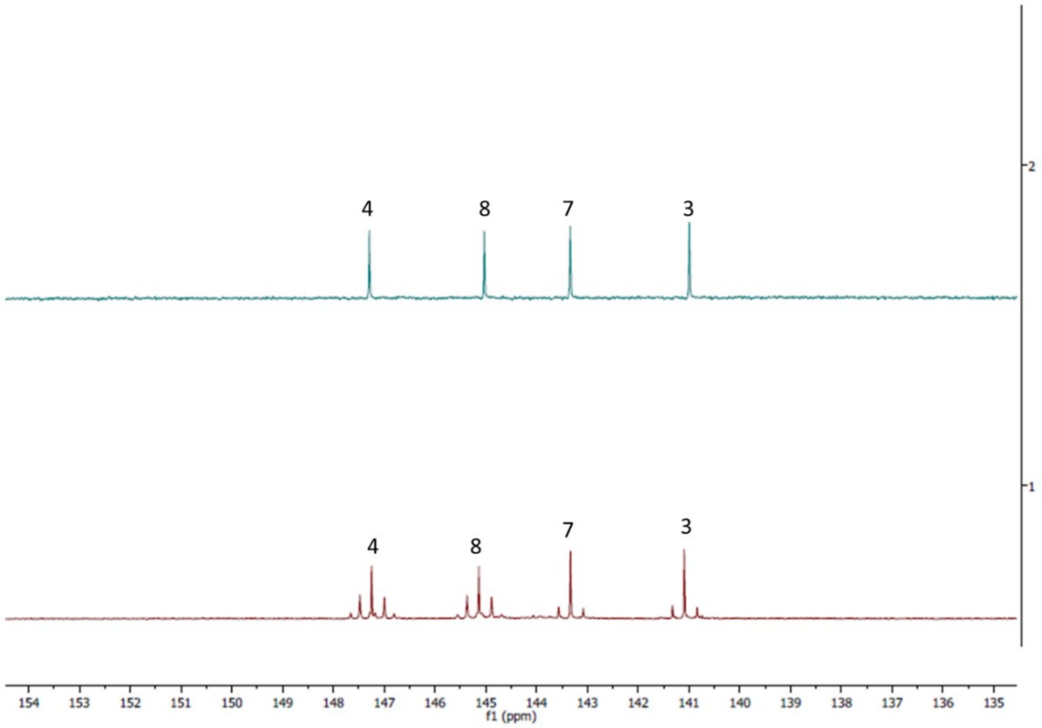


**Figure S73**: $^{13}C$ NMR of unlabelled (top) and labelled (bottom) zopfiellin **15** from [1,2-$^{13}C_2$] acetate (125 MHz, $CDCl_3$)

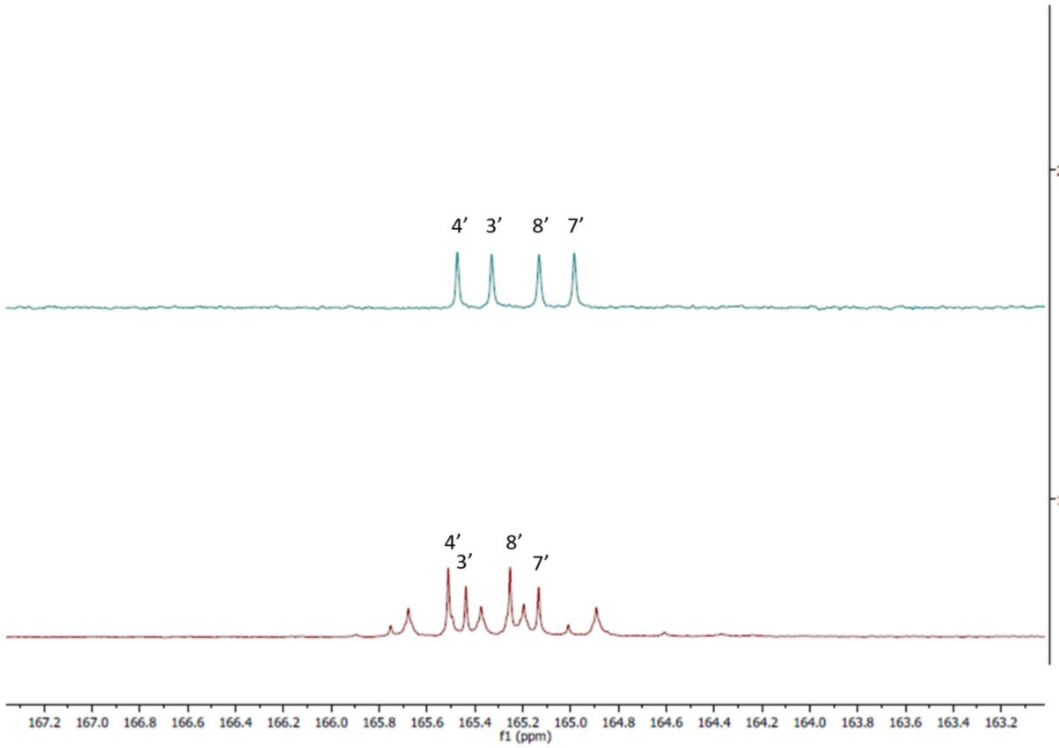


**Figure S74**: $^{13}C$ NMR of unlabelled (top) and labelled (bottom) zopfiellin **15** from [1,2-$^{13}C_2$] acetate (125 MHz, $CDCl_3$)

## Feeding Compounds to *Diffractella curvata* Strain Δ*zopPKS*

Δ*zopPKS* was fed with scytalidin **16** or deoxyscytalidin **17** purified from *S. album* cultures. 5-hydroxy-deoxyscytalidin **19**, purified from Δ*zopPKS* cultures fed with deoxyscytalidin, was also fed back to strain Δ*zopPKS*. To achieve these feeding studies, a seed culture of Δ*zopPKS* was homogenised and used to inoculate 500 mL non-baffled flasks containing 100 mL PDB. After 3 days, 5 mg per 100 mL flask, of compounds **16**, **17** or **19** (solubilized in DMSO or MeOH at a concentration of 20 mg/ mL) were fed. The feeding was repeated on day 5. After 12- 14 days the cultures were extracted with EtOAc and the products analysed using LCMS.

To isolate compounds **6**, **20** and **21**, a total of 150 mg of scytalidin was fed to 15 x 100 mL cultures of Δ*zopPKS*. The cultures were extracted with EtOAc and preparative LCMS using a 40 – 95 % gradient yielded 10 mg of **21**, 3.4 mg of **20** and 0.7 mg of **6**.

## Synthesis

All reagents were sourced from commercial suppliers and were used without further purification unless stated otherwise. All reactions using anhydrous solvents were performed using standard Schlenk syringe-septa techniques, with flame dried glassware under a positive pressure of nitrogen. Anhydrous DCM was dried by passing through a modified Grubbs system of alumina columns, manufactured by Anhydrous Engineering. Flash column chromatography was performed according to the procedures used by Still et al.[35] using silica gel (Fisher Scientific or Aldrich) and a suitable eluent.

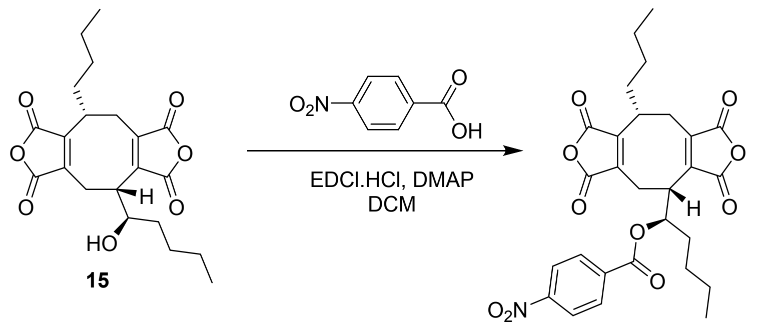


Zopfiellin (**15**) (28 mg, 72.0 µmol) was dissolved in anhydrous DCM (0.4 mL) under a nitrogen atmosphere and the solution was cooled to 0 °C. *p*-Nitrobenzoic acid (15 mg, 93.0 µmol), EDCI.HCl (17 mg, 93.0 µmol) and DMAP (3 mg, 18.0 µmol) were added and the reaction mixture was stirred at 0 °C for 3 h. The reaction mixture was then warmed to room temperature and stirred for 12 h. The reaction mixture was diluted with DCM (10 mL) and quenched with saturated aqueous $NH_4Cl$ (5 mL). The layers were separated, and the aqueous layer was extracted further with DCM (10 mL x 2). The combined organic layers were dried over $MgSO_4$, filtered and concentrated under reduced pressure. Purification by silica gel column chromatography (10% ethyl acetate in petroleum ether) gave *p*-nitrobenzoate **18** (23 mg, 60%) as a white solid. Recrystallisation by slow evaporation from an ethyl acetate:hexane mixture gave **18** as colourless crystals; $\delta_H$ (400 MHz, $CDCl_3$) 0.90 (3H, t, *J* 7.0, 5''-$H_3$), 0.95 (3H, t, *J* 7.0, 4'''-$H_3$), 1.24-1.38 (4H, m, 2'''-$H_2$ and 3'''-$H_2$), 1.38-1.49 (4H, m, 3''-$H_2$ and 4''-$H_2$), 1.50- 1.63 (1H, m, 1'''-*H*H), 1.72-1.82 (1H, m, 1'''-H*H*), 1.82-1.91 (1H, m, 2''-*H*H), 1.92-2.01 (1H, m, 2''-H*H*), 2.72 (1H, *app.* t, *J* 13.5, 6-*H*H), 2.78 (1H, dd, *J* 13.5 and 5.5, 6-H*H*), 2.88-2.96 (1H, m, 5-H), 3.13-3.24 (2H, m, 2-$H_2$), 3.31 (1H, ddd, *J* 12.0, 6.0 and 2.5, 1-H), 5.58 (1H, ddd, *J* 8.0, 5.0 and 2.5, 1''-H), 8.15 (2H, d, *J* 9.0, Ar*H*), 8.33 (2H, d, *J* 9.0, Ar*H*); $\delta_C$ (100 MHz, $CDCl_3$) 13.9 (C-5''), 14.0 (C-4'''), 19.9 (C-2), 22.38 (C-4''), 22.40 (C-3'''), 24.7 (C-6), 27.9 (C-3''), 28.6 (C-2'''), 31.9 (C-2''), 34.1 (C-1'''), 36.9 (C-5), 40.8 (C-1), 75.3 (C-1''), 124.1 (Ar), 130.9 (Ar), 134.3 (Ar), 139.7 (C-3), 142.2 (C-7), 144.0 (C-8), 147.7 (C-4), 151.1 (Ar), 164.3 (CO), 164.7 (CO), 164.8 (CO), 164.8 (CO), 165.3 (CO); $\nu_{max}$/cm$^{-1}$ 2959, 2932, 1850 (C=O), 1769 (C=O), 1725 (C=O), 1528, 1264; $[\alpha]_D^{24}$ -127.5 (*c*. 0.40, $CHCl_3$).

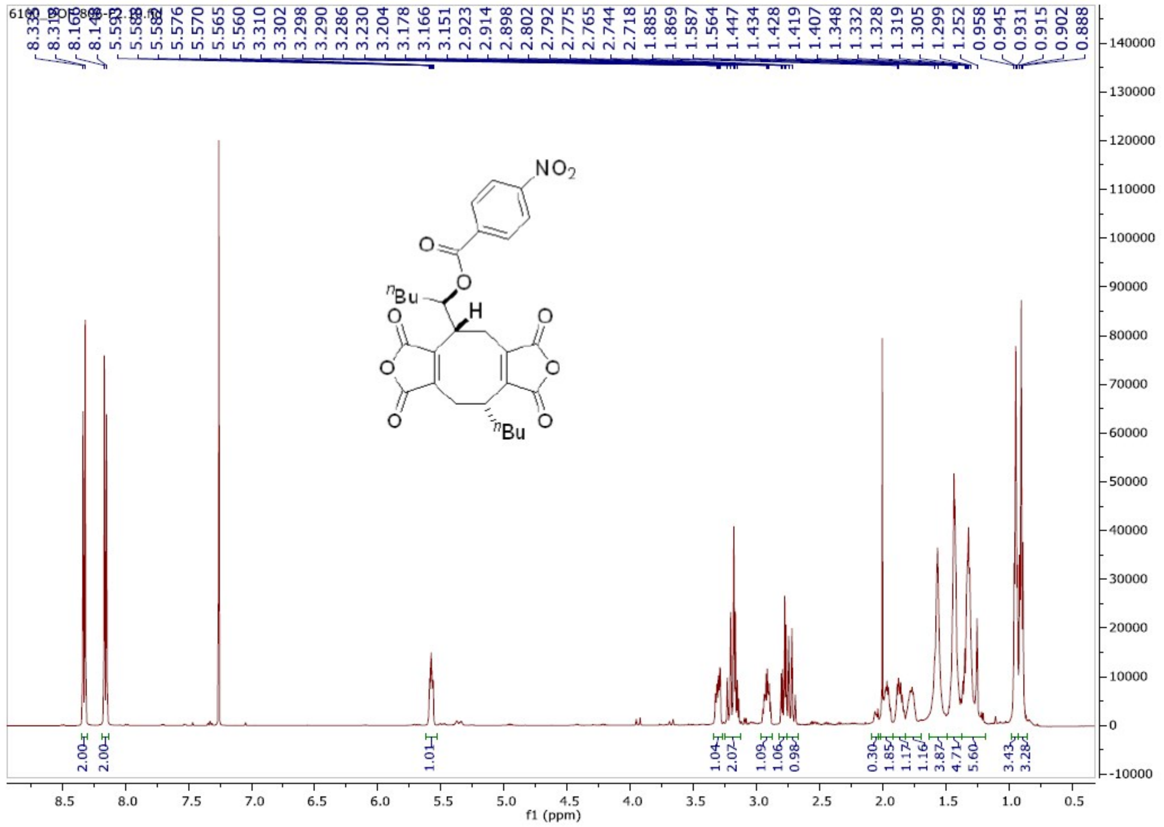

**Figure S75**: $^{1}$H NMR spectrum (400 MHz, $CDCl_3$) of **18**.

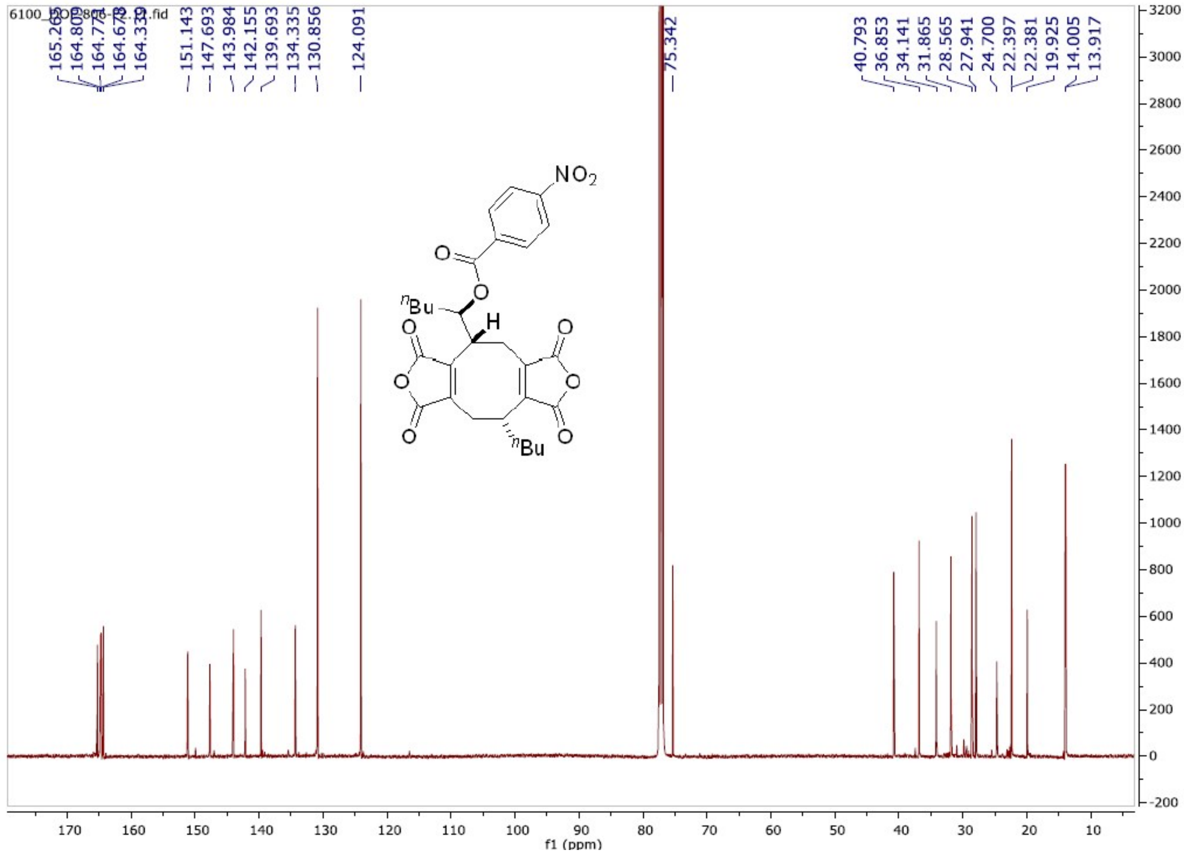

**Figure S76:** $^{13}$C NMR spectrum (100 MHz, $CDCl_3$) of **18**.

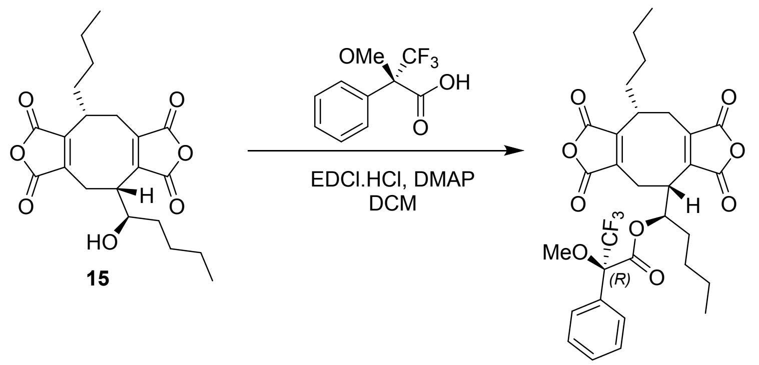


Zopfiellin (**15**) (73 mg, 0.19 mmol) was dissolved in anhydrous DCM (0.5 mL) under a nitrogen atmosphere at 0 °C. EDCI.HCl (54 mg, 0.28 mmol) and DMAP (6 mg, 0.05 mmol) were added, followed by (R)-(+)-α-methoxy-α-trifluoromethylphenylacetic acid (66 mg, 0.28 mmol, dissolved in anhydrous DCM (0.5 mL)). The reaction mixture was then stirred at room temperature for 12 h. The reaction was diluted with DCM (5 mL) and quenched with saturated aqueous $NH_4Cl$ solution (2 mL). The layers were separated, and the aqueous layer was extracted further with DCM (10 mL x 2). The organic layers were combined, dried over $MgSO_4$, filtered and concentrated under reduced pressure. Purification by silica gel column chromatography (7-15% ethyl acetate in petroleum ether) gave R-MTPA-zopfiellin (44 mg, 38%) as a pale yellow oil; $\delta_H$ (400 MHz, $CDCl_3$) 0.91-0.98 (6H, m, 5''-$H_3$ and 4'''-$H_3$), 1.20-1.59 (9H, m, 3''-$H_2$, 4''-$H_2$, 1'''-*H*H, 2'''-$H_2$ and 3'''-$H_2$), 1.64-1.77 (2H, m, 2''-*H*H and 1'''-H*H*), 1.95-2.04 (1H, m, 2''-H*H*), 2.33 (1H, dd, *J* 13.5 and 5.5, 6-*H*H), 2.45 (1H, *app.* t, *J* 13.5, 6-H*H*), 2.77- 2.85 (1H, m, 5-H), 2.96-3.04 (2H, m, 2-$H_2$), 3.13-3.19 (1H, m, 1-H), 3.33 (3H, s, $OCH_3$), 5.36 (1H, td, *J* 7.5 and 1.5, 1''-H), 7.36-7.43 (4H, m, Ar*H*), 7.48 (1H, tt, *J* 7.0 and 2.5, Ar*H*); $\delta_C$ (100 MHz, $CDCl_3$) 13.91 (C-5''), 13.94 (C-4'''), 18.9 (C-2), 22.3 (C-4''), 22.4 (C-3'''), 24.2 (C-6), 27.3 (C-3''), 28.2 (C-2'''), 31.1 (C-2''), 34.0 (C-1'''), 36.3 (C-5), 39.3 (C-1), 55.0 ($OCH_3$), 76.5 (C-1''), 85.0 (d, $^2J_{CF}$ 28.0, $COCH_3$), 123.5 (q, $^1J_{CF}$ 288.0, $CF_3$), 127.8 (d, $^3J_{CF}$ 1.5, Ar), 129.1 (Ar), 130.6 (Ar), 130.8 (Ar), 139.8 (C-3), 143.0 (C-8), 143.6 (C-7), 147.8 (C-4), 164.5 (CO), 164.8 (CO), 164.9 (CO), 165.2 (CO), 166.0 (CO); $\delta_F$ (376 MHz, $CDCl_3$) -70.2; $\nu_{max}$/$cm^{-1}$ 2959, 2934, 1850 (C=O), 1768 (C=O), 1467, 1252, 1169; *m/z* (ESI) found: 629.1968 $[M+Na]^+$, ($C_{31}H_{33}F_3O_9Na$ requires 629.1969); $[\alpha]_D^{23}$ -45.0 (*c*. 0.40, $CHCl_3$).

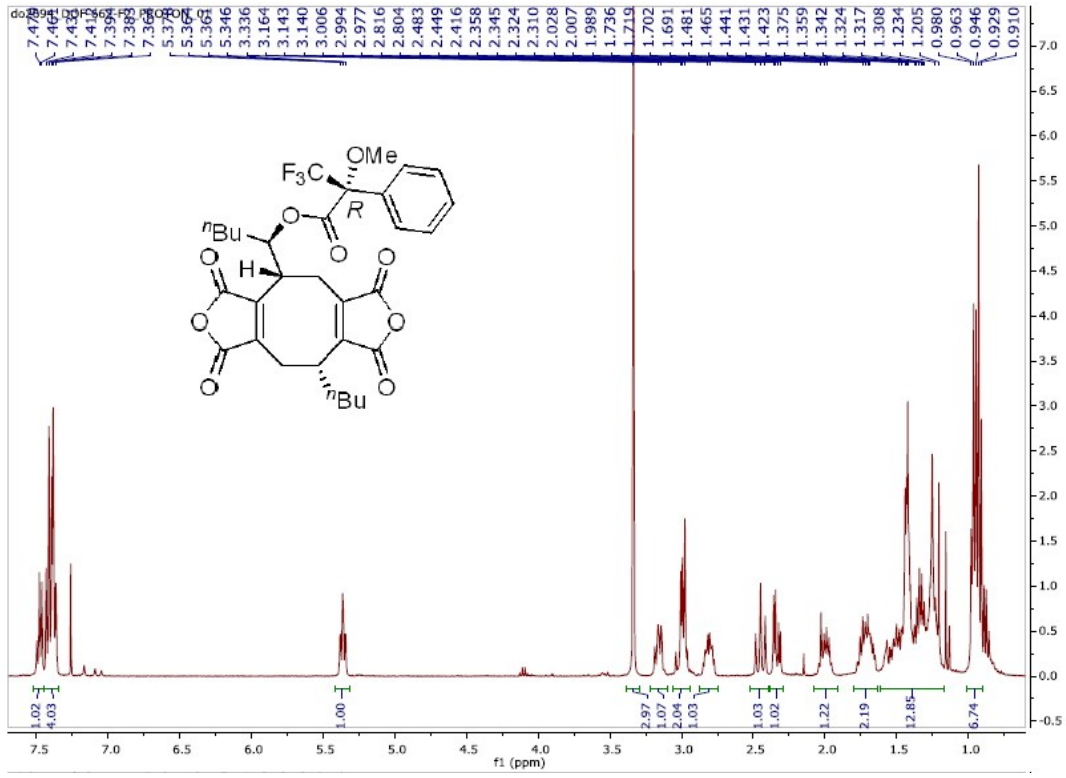


**Figure S77:** $^{1}$H NMR spectrum (100 MHz, $CDCl_3$) of ***R*-MTPA-zopfiellin**

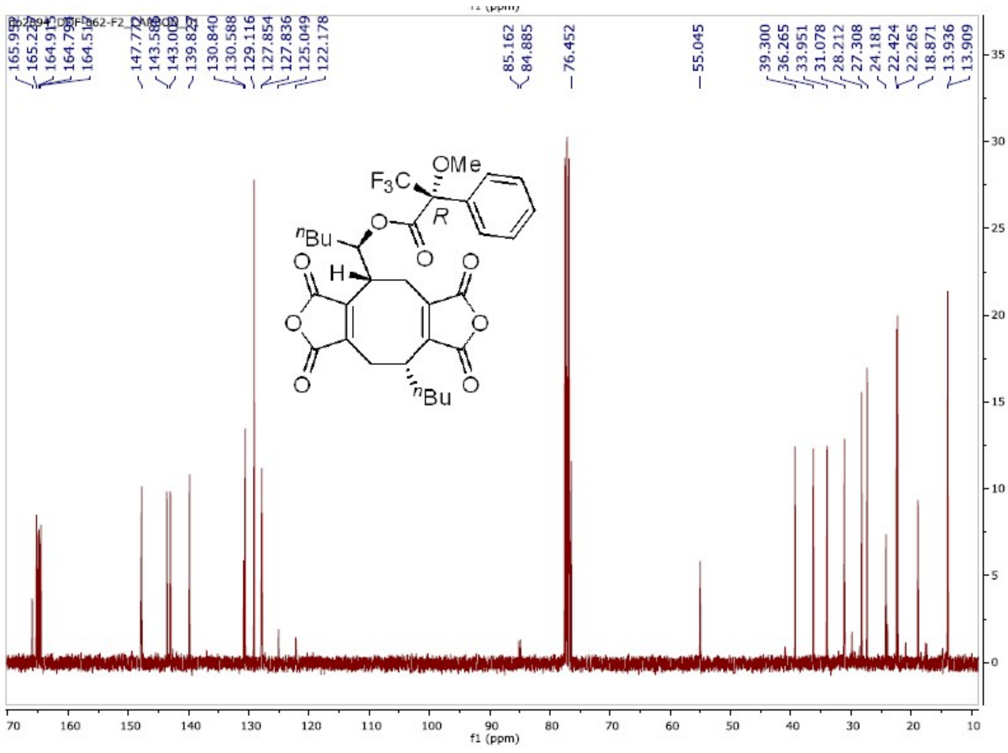


**Figure S78**: $^{13}$C NMR spectrum (100 MHz, $CDCl_3$) of ***R*-MTPA-zopfiellin**

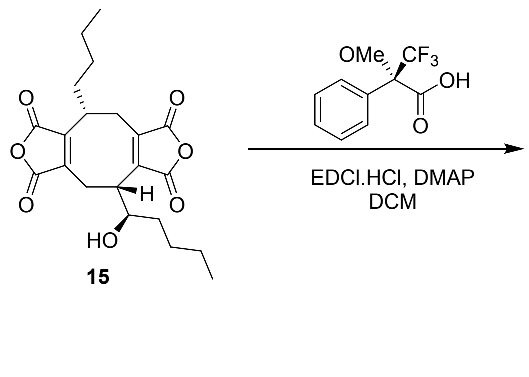


Zopfiellin (**15**) (77 mg, 0.20 mmol) was dissolved in anhydrous DCM (0.5 mL) under a nitrogen atmosphere at 0 °C. EDCI.HCl (58 mg, 0.30 mmol) and DMAP (6 mg, 0.05 mmol) were added, followed by (*S*)-(-)-α-methoxy-α-trifluoromethylphenylacetic acid (70 mg, 0.30 mmol, dissolved in anhydrous DCM (0.5 mL)). The reaction mixture was then stirred at room temperature for 12 h. The reaction was diluted with DCM (5 mL) and quenched with saturated aqueous $NH_4Cl$ solution (2 mL). The layers were separated, and the aqueous layer was extracted further with DCM (10 mL x 2). The organic layers were combined, dried over $MgSO_4$, filtered and concentrated under reduced pressure. Purification by silica gel column chromatography (15% ethyl acetate in petroleum ether) gave *S*-MTPA-zopfiellin (53 mg, 43%) as a colourless needles; $\delta_H$ (400 MHz, $CDCl_3$) 0.95 (3H, t, *J* 7.0, 4'''-$H_3$), 0.98 (3H, t, *J* 7.0, 5''-$H_3$), 1.20-1.57 (9H, m, 3''-$H_2$, 4''-$H_2$, 1'''-*H*H, 2'''-$H_2$ and 3'''-$H_2$), 1.66-1.74 (1H, m, 1'''-H*H*), 1.74- 1.83 (1H, m, 2''-*H*H), 1.95-2.02 (1H, m, 2''-H*H*), 2.09 (1H, dd, *J* 14.0 and 5.0, 6-*H*H), 2.30 (1H, *app.* t, *J* 14.0, 6-H*H*), 2.70-2.77 (1H, m, 5-H), 2.97 (1H, dd, *J* 13.0 and 5.5, 2-*H*H), 3.03 (1H, *app.* t, *J* 13.0, 2-H*H*), 3.13 (1H, dd, *J* 12.0 and 5.5, 1-H), 3.58 (3H, s, $OCH_3$), 5.39 (1H, td, *J* 7.0 and 1.5, 1''-H), 7.35-7.47 (5H, m, Ar*H*); $\delta_C$ (100 MHz, $CDCl_3$) 13.9 (C-5''), 14.0 (C-4'''), 18.8 (C-2), 22.3 (C-4''), 22.4 (C-3'''), 23.9 (C-6), 27.4 (C-3''), 28.3 (C-2'''), 31.5 (C-2''), 33.9 (C-1'''), 36.3 (C-5), 39.3 (C-1), 55.8 ($OCH_3$), 75.7 (C-1''), 84.2 (d, $^2J_{CF}$ 27.5, $COCH_3$), 123.1 (q, $^1J_{CF}$ 289.0, $CF_3$), 126.8 (Ar), 128.9 (Ar), 130.4 (Ar), 132.3 (Ar), 139.7 (C-3), 142.5 (C-8), 143.7 (C-7), 147.8 (C-4), 164.3 (CO), 164.7 (CO), 164.8 (CO), 165.3 (CO), 165.9 (CO); $\delta_F$ (376 MHz, $CDCl_3$) -70.8; $\nu_{max}$/cm$^{-1}$ 2958, 2934, 2873, 1850 (C=O), 1770 (C=O), 1467, 1252, 1187, 1169; *m/z* (ESI) found: 629.1968 $[M+Na]^+$, ($C_{31}H_{33}F_3O_9Na$ requires 629.1969); $[\alpha]_D^{22}$ - 40.8 (*c*. 0.49, $CHCl_3$).

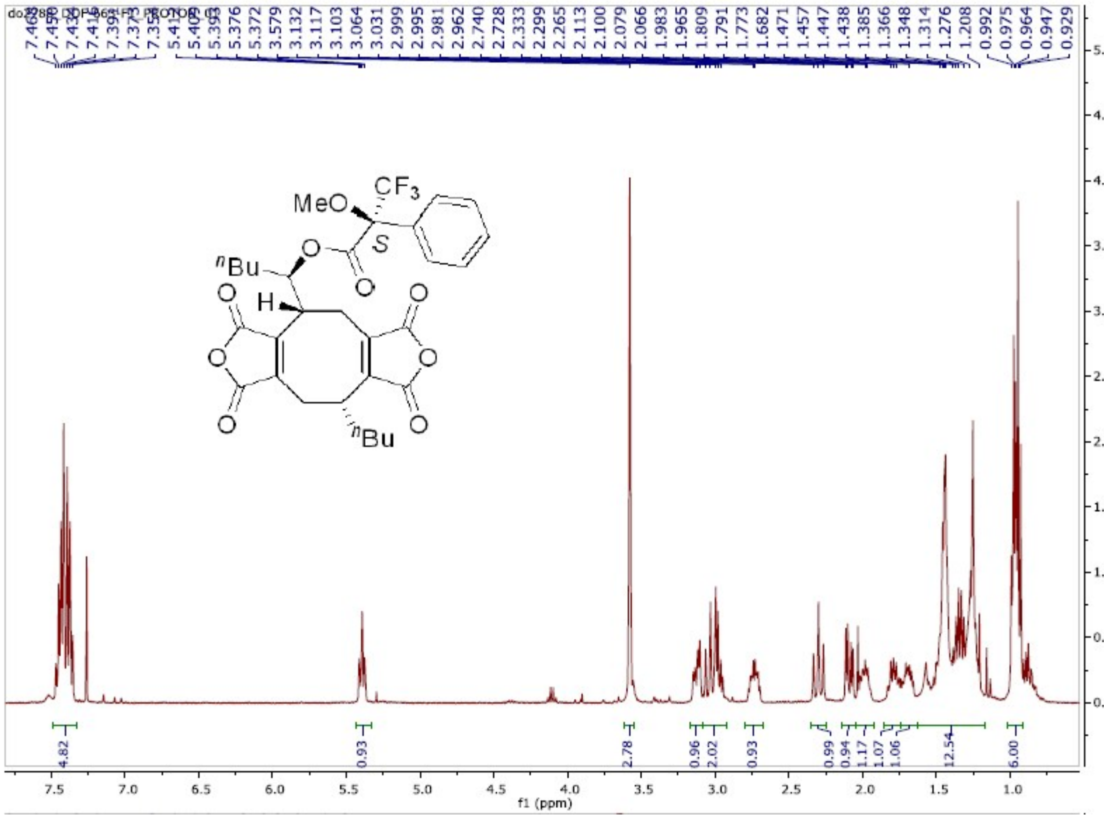

**Figure S79**: $^{1}$H NMR spectrum (100 MHz, $CDCl_3$) of ***S*-MTPA-zopfiellin**

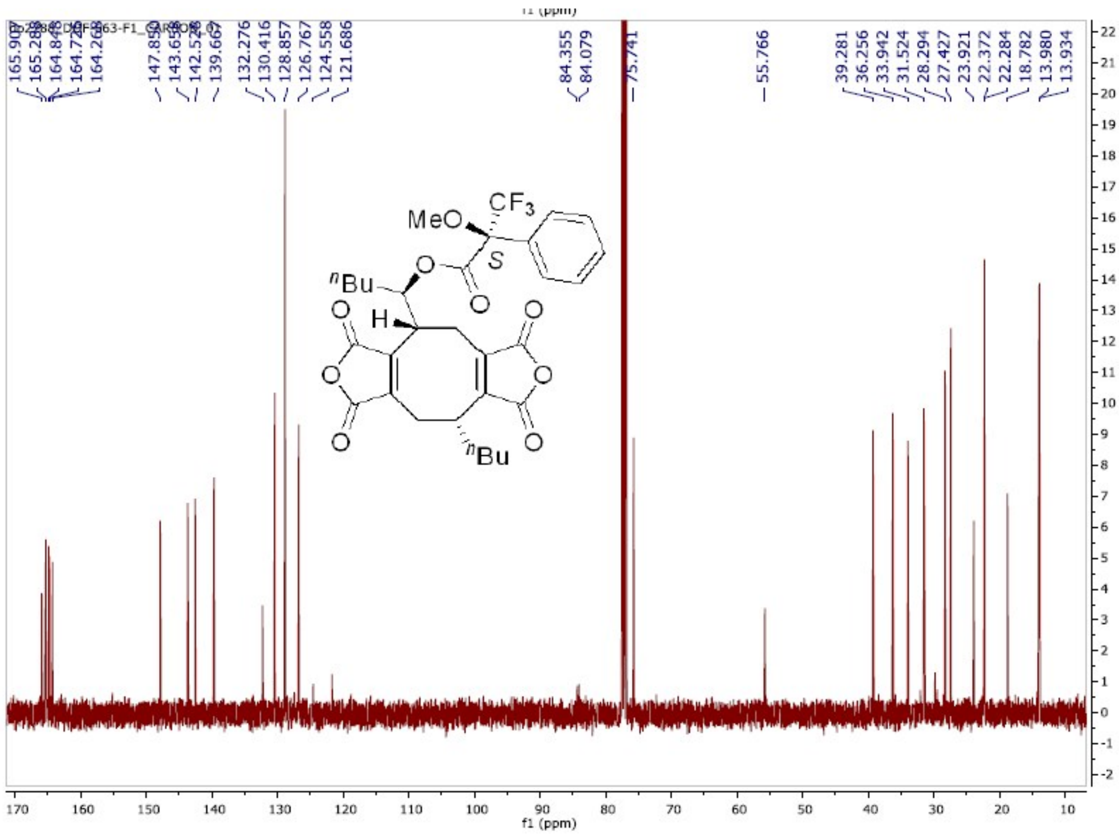

**Figure S80**: $^{13}$C NMR spectrum (100 MHz, $CDCl_3$) of ***S*-MTPA-zopfiellin**

## X-Ray Crystallography

X-ray diffraction experiments on **16** and **18** were carried out at 100(2) K on a Bruker APEX II diffractometer using Mo-$K_{\alpha}$ radiation (λ = 0.71073 Å), while **17** was carried out at 100(2) K Bruker Microstar rotating anode diffractometer using Cu-$K_{\alpha}$ (λ = 1.54178 Å). Intensities were integrated in SAINT[36] from several series of exposures measuring 0.5° in ω or ϕ and absorption corrections based on equivalent reflections were applied using SADABS.[37] Structures **16** and **17** were solved using ShelXS[38], while **18** was solved using ShelXT[39] all of the structures were refined by full matrix least squares against $F^2$ in ShelXL[38, 40] using Olex2[41]. All of the non-hydrogen atoms were refined anisotropically. While all of the hydrogen atoms were located geometrically and refined using a riding model. In the case of **16** the molecule displayed disorder, the occupancies of the fragments was determined by refining them against a free variable with the sum of the two sites set to equal 1, the occupancies were then fixed at the refined values and restraints were used to maintain sensible geometries and thermal parameters. In addition Squeeze within Platon[42, 43] was used to remove disordered solvent from the lattice of **16** that could not be sensibly modelled. Crystal structure and refinement data are given in Table S5. Crystallographic data for compounds **16**, **17** and **18** have been deposited with the Cambridge Crystallographic Data Centre as supplementary publication CCDC 2011262-2011264. Copies of the data can be obtained free of charge on application to CCDC, 12 Union Road, Cambridge CB2 1EZ, UK [fax(+44) 1223 336033, e-mail: deposit@ccdc.cam.ac.uk].

**Table S12**: Crystal data and structure refinement for **16**, **17** and **18**.

| Identification code | **16** | **17** | **18** |
|---|---|---|---|
| Empirical formula | $C_{22}H_{28}O_7$ | $C_{22}H_{28}O_6$ | $C_{28}H_{29}NO_{10}$ |
| Formula weight | 404.44 | 388.44 | 539.52 |
| Temperature/K | 100(2) | 100.01 | 100(2) |
| Crystal system | orthorhombic | orthorhombic | monoclinic |
| Space group | $P2_12_12_1$ | $P2_12_12_1$ | $P2_1$ |
| $a$/Å | 8.0210(2) | 11.617(2) | 11.3164(3) |
| $b$/Å | 10.0682(2) | 12.880(3) | 6.93730(10) |
| $c$/Å | 29.3914(7) | 27.194(5) | 17.3840(4) |
| α/° | 90 | 90 | 90 |
| β/° | 90 | 90 | 102.274(2) |
| γ/° | 90 | 90 | 90 |
| Volume/Å$^3$ | 2373.56(9) | 4069.1(14) | 1333.54(5) |
| Z | 4 | 8 | 2 |
| $\rho_{calc}$g/cm$^3$ | 1.132 | 1.268 | 1.344 |
| μ/mm$^{-1}$ | 0.084 | 0.751 | 0.103 |
| F(000) | 864.0 | 1664.0 | 568.0 |
| Crystal size/mm$^3$ | 0.577 × 0.408 × 0.226 | 0.288 × 0.23 × 0.127 | 0.492 × 0.273 × 0.149 |
| Radiation | MoKα (λ = 0.71073) | CuKα (λ = 1.54178) | MoKα (λ = 0.71073) |
| 2θ range for data collection/° | 4.276 to 55.872 | 6.5 to 133.684 | 3.684 to 55.916 |
| Index ranges | -10 ≤ h ≤ 10, -13 ≤ k ≤ 13, -38 ≤ l ≤ 35 | -13 ≤ h ≤ 11, -13 ≤ k ≤ 15, -32 ≤ l ≤ 27 | -14 ≤ h ≤ 14, -9 ≤ k ≤ 9, -22 ≤ l ≤ 22 |
| Reflections collected | 21576 | 26176 | 24109 |
| Independent reflections | 5685 [$R_{int}$ = 0.0372, $R_{sigma}$ = 0.0385] | 7142 [$R_{int}$ = 0.0532, $R_{sigma}$ = 0.0454] | 6323 [$R_{int}$ = 0.0368, $R_{sigma}$ = 0.0361] |
| Data/restraints/parameters | 5685/105/306 | 7142/0/509 | 6323/1/354 |
| Goodness-of-fit on $F^2$ | 1.059 | 1.048 | 1.031 |
| Final R indexes [I>=2σ (I)] | $R_1$ = 0.0547, $wR_2$ = 0.1346 | $R_1$ = 0.0539, $wR_2$ = 0.1383 | $R_1$ = 0.0339, $wR_2$ = 0.0709 |
| Final R indexes [all data] | $R_1$ = 0.0678, $wR_2$ = 0.1410 | $R_1$ = 0.0541, $wR_2$ = 0.1386 | $R_1$ = 0.0411, $wR_2$ = 0.0739 |
| Largest diff. peak/hole / e Å$^{-3}$ | 0.29/-0.32 | 0.47/-0.35 | 0.23/-0.20 |

## Assignment of zopfiellin stereochemistry

The substituent eclipsing the phenyl group of the MTPA unit experiences an aromatic shielding interaction resulting in an upfield chemical shift for protons affected. From comparing the chemical shifts, it is evident that the phenyl group in (*S*)-MTPA-zopfiellin shields the cyclooctadiene ring protons (6$H_2$ and 5-H) as apparent by their significant upfield shift relative to (*R*)-MTPA (Figure S72). The opposite effect is observed for 2"-*H*H which shifts downfield in (*S*)-MTPA-zopfiellin. This indicates that the 2"-*H*H signal is more shielded in (*R*)-MTPA-zopfiellin than it is in (*S*)-MTPA-zopfiellin.

These results reveal that the phenyl group in (*S*)-MTPA-zopfiellin interacts with the cyclooctadiene ring rather than the alkyl chain. Inputting this information into the *syn*-co-planar conformation for the (*S*)-MTPA derivative gives $R_1$ as the alkyl chain and $R_2$ as the cyclooctadiene ring. The absolute stereochemistry of the secondary alcohol has therefore been assigned as *R*.

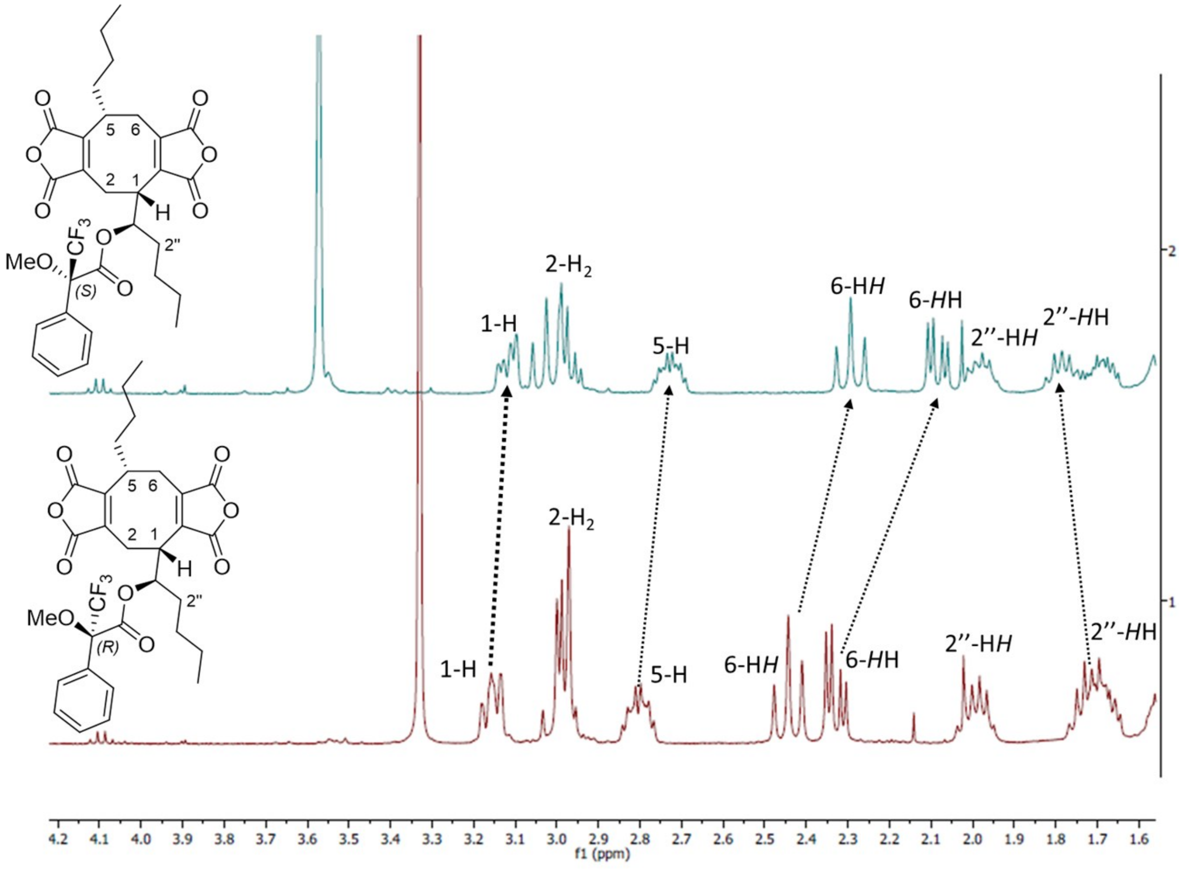


**Figure S81:** Comparison of $^1$H NMR (400 MHz, $CDCl_3$) of (S)-MTPA-zopfiellin (top) and (R)-MTPA-zopfiellin (bottom).

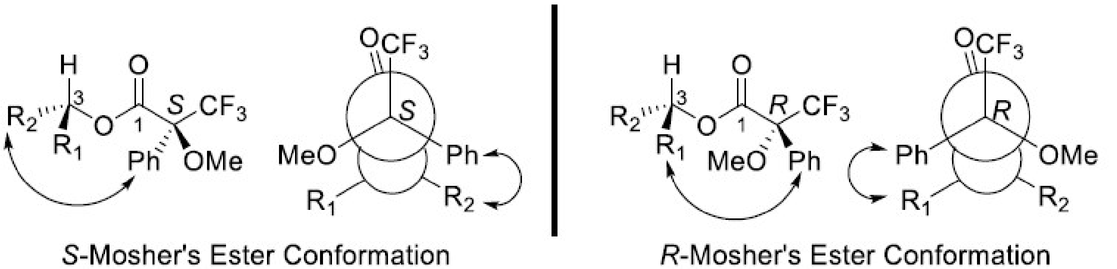


**Figure S82**: Representation of the spectroscopically dominant conformation in Mosher's ester analysis.

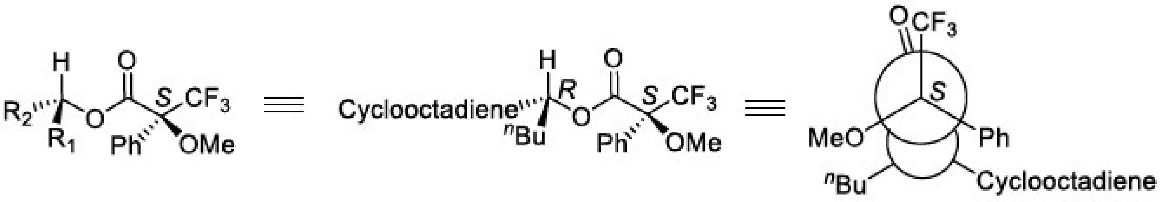


**Figure S83**: Mosher's ester analysis results viewed in the syn-co-planar conformation model.

## References


1. G. G. Silva, B. E. Dutilh, T. D. Matthews, K. Elkins, R. Schmieder, E. A. Dinsdale and R. A. Edwards, *Source Code Biol. Med.*, 2013, **8**, 23.
2. C. Trapnell, L. Pachter and S. L. Salzberg, *Bioinformatics*, 2009, **25**, 1105-1111.
3. B. Tudzynski, M. Mihlan, M. C. Rojas, P. Linnemannstons, P. Gaskin and P. Hedden, *J. Biol. Chem.*, 2003, **278**, 28635-28643.
4. M. D. Lebar, J. W. Cary, R. Majumdar, C. H. Carter-Wientjes, B. M. Mack, Q. Wei, V. Uka, S. De Saeger and J. D. Di Mavungu, *Fungal Genet. Biol.*, 2018, **116**, 14-23.
5. M. F. Grau, R. Entwistle, Y.-M. Chiang, M. Ahuja, C. E. Oakley, T. Akashi, C. C. C. Wang, R. B. Todd and B. R. Oakley, *ACS Chem. Biol.*, 2018, **13**, 3193-3205.
6. E. Geiser, S. K. Przybilla, A. Friedrich, W. Buckel, N. Wierckx, L. M. Blank and M. Bolker, *Microb. Biotechnol.*, 2016, **9**, 116-126.
7. L. Blank, J. Green and J. R. Guest, *Microbiology-Sgm*, 2002, **148**, 133-146.
8. K. Williams, A. J. Szwalbe, N. P. Mulholland, J. L. Vincent, A. M. Bailey, C. L. Willis, T. J. Simpson and R. J. Cox, *Angew. Chem., Int. Ed.*, 2016, **55**, 6784-6788.
9. J. I. Jimenez, J. F. Juarez, J. L. Garcia and E. Diaz, *Environ. Microbiol.*, 2011, **13**, 1718-1732.
10. F. Chardon and C. Damerval, *J. Mol. Evol.*, 2005, **61**, 579-590.
11. G. Yang, M. S. Rose, B. G. Turgeon and O. C. Yoder, *Plant Cell*, 1996, **8**, 2139-2150.
12. D. W. Brown, S.-H. Lee, L.-H. Kim, J.-G. Ryu, S. Lee, Y. Seo, Y. H. Kim, M. Busman, S.-H. Yun, R. H. Proctor and T. Lee, *Mol. Plant. Microbe Interact.*, 2015, **28**, 319-332.
13. L. Eichinger, J. A. Pachebat, G. Glockner, M.-A. Rajandream, R. Sucgang, M. Berriman, J. Song, R. Olsen, K. Szafranski, Q. Xu, B. Tunggal, S. Kummerfeld, M. Madera, B. A. Konfortov, F. Rivero, A. T. Bankier, R. Lehmann, N. Hamlin, R. Davies, P. Gaudet, P. Fey, K. Pilcher, G. Chen, D. Saunders, E. Sodergren, P. Davis, A. Kerhornou, X. Nie, N. Hall, C. Anjard, L. Hemphill, N. Bason, P. Farbrother, B. Desany, E. Just, T. Morio, R. Rost, C. Churcher, J. Cooper, S. Haydock, N. van Driessche, A. Cronin, I. Goodhead, D. Muzny, T. Mourier, A. Pain, M. Lu, D. Harper, R. Lindsay, H. Hauser, K. James, M. Quiles, M. M. Babu, T. Saito, C. Buchrieser, A. Wardroper, M. Felder, M. Thangavelu, D. Johnson, A. Knights, H. Loulseged, K. Mungall, K. Oliver, C. Price, M. A. Quail, H. Urushihara, J. Hernandez, E. Rabbinowitsch, D. Steffen, M. Sanders, J. Ma, Y. Kohara, S. Sharp, M. Simmonds, S. Spiegler, A. Tivey, S. Sugano, B. White, D. Walker, J. Woodward, T. Winckler, Y. Tanaka, G. Shaulsky, M. Schleicher, G. Weinstock, A. Rosenthal, E. C. Cox, R. L. Chisholm, R. Gibbs, W. F. Loomis, M. Platzer, R. R. Kay, J. Williams, P. H. Dear, A. A. Noegel, B. Barrell and A. Kuspa, *Nature*, 2005, **435**, 43-57.
14. Y.-C. Huang, Y.-H. Chen, S.-R. Lo, C.-I. Liu, C.-W. Wang and W.-T. Chang, *Mol. Microbiol.*, 2004, **53**, 81-91.
15. D. K. Holm, L. M. Petersen, A. Klitgaard, P. B. Knudsen, Z. D. Jarczynska, K. F. Nielsen, C. H. Gotfredsen, T. O. Larsen and U. H. Mortensen, *Chem. Biol.*, 2014, **21**, 519-529.
16. K. Katayama, T. Kobayashi, H. Oikawa, M. Honma and A. Ichihara, *Biochim. Biophys. Acta Protein Struct. Mol. Enzymol.*, 1998, **1384**, 387-395.
17. W. Kim, C.-M. Park, J.-J. Park, H. O. Akamatsu, T. L. Peever, M. Xian, D. R. Gang, G. Vandemark and W. Chen, *Mol. Plant. Microbe Interact.*, 2015, **28**, 482-496.
18. P. Chankhamjon, D. Boettger-Schmidt, K. Scherlach, B. Urbansky, G. Lackner, D. Kalb, H.-M. Dahse, D. Hoffmeister and C. Hertweck, *Angew. Chem., Int. Ed.*, 2014, **126**, 13627-13631.
19. K. E. Lebe and R. J. Cox, *Chem. Sci.*, 2019, **10**, 1227-1231.
20. B. Bonsch, V. Belt, C. Bartel, N. Duensing, M. Koziol, C. M. Lazarus, A. M. Bailey, T. J. Simpson and R. J. Cox, *Chem. Commun.*, 2016, **52**, 6777-6780.
21. A. Zajc, M. J. Romão, B. Turk and R. Huber, *J. Mol. Biol.*, 1996, **263**, 269-283.
22. C. A. Cuomo, W. A. Untereiner, L.-J. Ma, M. Grabherr and B. W. Birren, *Genome Announce.*, 2015, **3**, e00021-00015.
23. M. S. Santisteban, T. Kalashnikova and M. M. Smith, *Cell*, 2000, **103**, 411-422.

24. S. Kumar, G. Stecher, M. Li, C. Knyaz and K. Tamura, *Mol. Biol. Evol.*, 2018, **35**, 1547-1549.
25. N. Saitou and M. Nei, *Mol. Biol. Evol.*, 1987, **10**, 471-483.
26. A. Bhattacharya, S. Kourmpetli, D. A. Ward, S. G. Thomas, F. Gong, S. J. Powers, E. Carrera, B. Taylor, F. N. de Caceres Gonzalez and B. Tudzynski, *Plant Physiol.*, 2012, **160**, 837-845.
27. S.-S. Gao, N. Naowarojna, R. Cheng, X. Liu and P. Liu, *Nat. Prod. Rep.*, 2018, **35**, 792-837.
28. R. Fujii, Y. Matsu, A. Minami, S. Nagamine, I. Takeuchi, K. Gomi and H. Oikawa, *Org. Lett.*, 2015, **17**, 5658-5661.
29. M. L. Nielsen, L. Albertsen, G. Lettier, J. B. Nielsen and U. H. Mortensen, *Fungal Genet. Biol.*, 2006, **43**, 54-64.
30. C. Greco, K. de Mattos-Shipley, A. M. Bailey, N. P. Mulholland, J. L. Vincent, C. L. Willis, R. J. Cox and T. J. Simpson, *Chem. Sci.*, 2019, **10**, 2930-2939.
31. K. Arai, S. Shimizu, H. Miyajima and Y. Yamamoto, *Chem. Pharm. Bull. (Tokyo)*, 1989, **37**, 2870-2872.
32. T. Watanabe, T. Yasumoto, M. Murata, M. Tagawa, H. Narushima, T. Furusato, M. Kuwahara, M. Hanaue and T. Seki, E. P. Office, EP0582267A1, 1994.
33. G. M. Strunz, M. Kakushima and M. A. Stillwell, *J. Chem. Soc. Perkin 1*, 1972, **18**, 2280-2283.
34. W. A. Ayer, P.-p. Lu, H. Orszanska and L. Sigler, *J. Nat. Prod.*, 1993, **56**, 1835-1838.
35. W. C. Still, M. Kahn and A. Mitra, *J. Org. Chem.*, 1978, **43**, 2923-2925.
36. Bruker, *SAINT+ v8.38A Integration Engine, Data Reduction Software, Bruker Analytical X-ray Instruments Inc., Madison, WI, USA*, 2015.
37. Bruker, *SADABS 2014/5, Bruker AXS area detector scaling and absorption correction, Bruker Analytical X-ray Instruments Inc., Madison, Wisconsin, USA*, 2014/5.
38. G. M. Sheldrick, *Acta Crystallogr., Sect. A: Found. Adv.*, 2008, **64**, 112-122.
39. G. M. Sheldrick, *Acta Crystallogr., Sect. A: Found. Adv.*, 2015, **71**, 3-8.
40. G. M. Sheldrick, *Acta Crystallogr., Sect. C: Struct. Chem.*, 2015, **71**, 3-8.
41. O. V. Dolomanov, L. J. Bourhis, R. J. Gildea, J. A. K. Howard and H. Puschmann, *J. Appl. Crystallogr.*, 2009, **42**, 339-341.
42. A. L. Spek, *J. Appl. Crystallogr.*, 2003, **36**, 7-13.
43. A. L. Spek, *Acta Crystallogr., Sect. D: Biol. Crystallogr.*, 2009, **65**, 148-155.
